# Enantiomeric β-sheet peptides from Aβ form homochiral pleated β-sheets rather than heterochiral rippled β-sheets†

**DOI:** 10.1039/d2sc02080g

**Published:** 2022-05-31

**Authors:** Xingyue Li, Stephanie E. Rios, James S. Nowick

**Affiliations:** Department of Chemistry, University of California Irvine 4126 Natural Sciences I Irvine CA 92697-2025 USA jsnowick@uci.edu; Department of Pharmaceutical Sciences, University of California Irvine 4126 Natural Sciences I Irvine CA 92697-2025 USA

## Abstract

In 1953, Pauling and Corey postulated “rippled” β-sheets, composed of a mixture of d- and l-peptide strands, as a hypothetical alternative to the now well-established structures of “pleated” β-sheets, which they proposed as a component of all-l-proteins. Growing interest in rippled β-sheets over the past decade has led to the development of mixtures of d- and l-peptides for biomedical applications, and a theory has emerged that mixtures of enantiomeric β-sheet peptides prefer to co-assemble in a heterochiral fashion to form rippled β-sheets. Intrigued by conflicting reports that enantiomeric β-sheet peptides prefer to self-assemble in a homochiral fashion to form pleated β-sheets, we set out address this controversy using two β-sheet peptides derived from Aβ_17–23_ and Aβ_30–36_, peptides 1a and 1b. Each of these peptides self-assembles to form tetramers comprising sandwiches of β-sheet dimers in aqueous solution. Through solution-phase NMR spectroscopy, we characterize the different species formed when peptides 1a and 1b are mixed with their respective d-enantiomers, peptides *ent*-1a and *ent*-1b. ^1^H NMR, DOSY, and ^1^H,^15^N-HSQC experiments reveal that mixing peptides 1a and *ent*-1a results in the predominant formation of homochiral tetramers, with a smaller fraction of a new heterochiral tetramer, and mixing peptides 1b and *ent*-1b does not result in any detectable heterochiral assembly. ^15^N-edited NOESY reveals that the heterochiral tetramer formed by peptides 1a and *ent*-1a is composed of two homochiral dimers. Collectively, these NMR studies of Aβ-derived peptides provide compelling evidence that enantiomeric β-sheet peptides prefer to self-assemble in a homochiral fashion in aqueous solution.

## Abstract 2

In aqueous solution, mixtures of l- and d- macrocyclic β-sheet peptides derived from Aβ self-assemble to form homochiral pleated β-sheets but do not co-assemble to form heterochiral rippled β-sheets.

## Graphical abstract

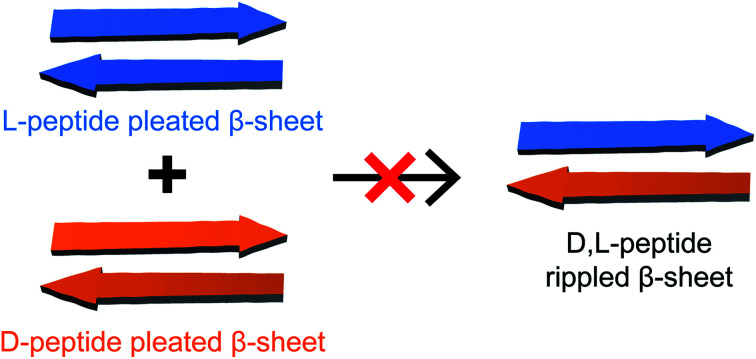

## Introduction

**Fig. 1:**
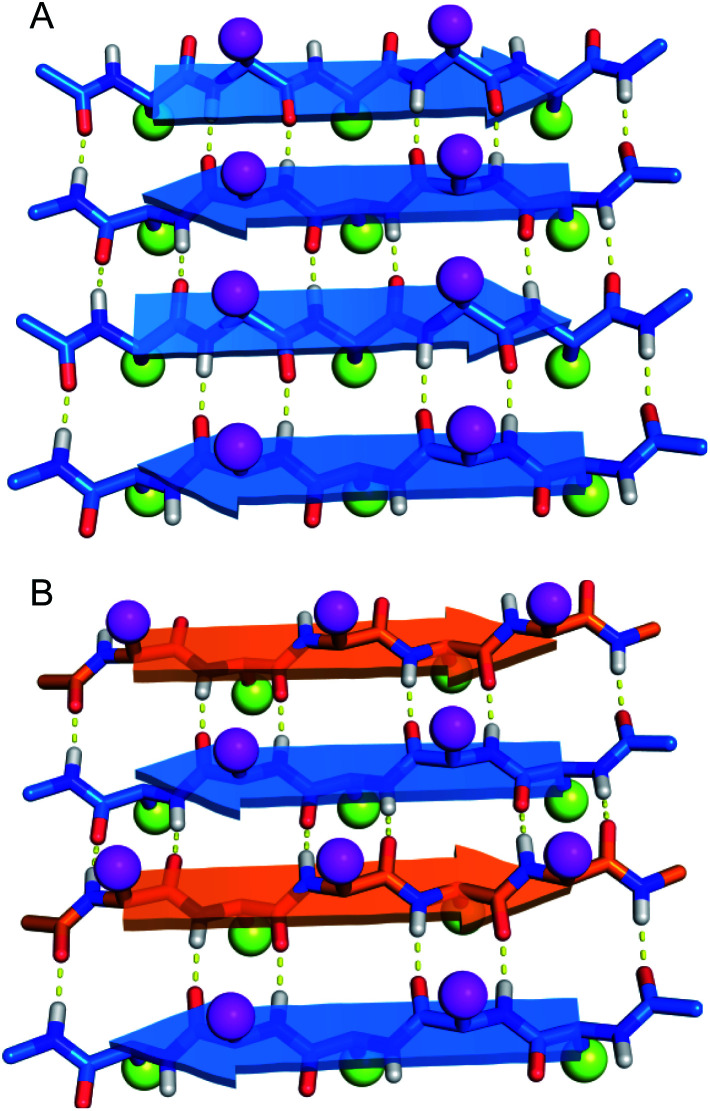
Molecular models of (A) pleated β-sheets formed from l-peptide strands (blue) and (B) rippled β-sheets formed from a mixture of l-peptide and d-peptide strands (orange). Amino acid side chains are depicted as balls, with magenta balls on the top face and yellow balls on the bottom face of the β-sheets.

Do enantiomeric β-sheet peptides prefer to self-assemble in a homochiral fashion or co-assemble in a heterochiral fashion? In the early 1950s, Pauling and Corey introduced the terms “pleated” β-sheets and “rippled” β-sheets to describe two types of β-sheet assembly.^1–3^ In both a pleated β-sheet and a rippled β-sheet, adjacent peptide strands hydrogen bond through edge-to-edge interactions (Fig. 1). Pleated β-sheets are composed of peptide strands of the same chirality (all l-peptide strands or all d-peptide strands), while peptide strands of opposite chirality (l-peptide strands and d-peptide strands) are required to form rippled β-sheets. In a pleated β-sheet, the side chains of sequential residues are oriented up-down-up-down and those of the adjacent peptide strands are also oriented up-down-up-down (Fig. 1A). In a rippled β-sheet, however, the side chains of sequential residues are oriented up-down-up-down and those of the adjacent peptide strands are oriented down-up-down-up (Fig. 1B). Although pleated β-sheets are a near ubiquitous feature of proteins, rippled β-sheets are not found in nature, because ribosomal proteins and peptides are composed only of l-amino acids.

Rippled β-sheets, formed by mixing d- and l-peptides, have recently attracted considerable interest as biomaterials and for other biomedical applications.^4–6^ Schneider and co-workers demonstrated the effects of chirality with the hydrogel-forming peptide MAX1. When MAX1 was mixed with an equimolar amount of its enantiomer, the resulting hydrogel showed four times greater rigidity than that of the enantiopure MAX1 peptide.^7,8^ Nilsson and co-workers demonstrated by isotope-edited IR spectroscopy and FRET studies that mixtures of enantiomeric peptides l-Ac-(FKFE)_2_-NH_2_ and d-Ac-(FKFE)_2_-NH_2_ form rippled β-sheet fibrils. The authors further demonstrated by isothermal titration calorimetry (ITC) that the resulting heterochiral assembly is more thermodynamically favored than the homochiral assembly.^9^ In a subsequent paper, the authors reported that the hydrogel formed by the heterochiral rippled β-sheets is stronger and more resistant to proteolytic degradation than the hydrogel formed by the homochiral l-pleated β-sheets.^10^

The co-assembly of enantiomeric β-sheet peptides is not limited to designed biomaterials, and has also been used to characterize and study fibril formation of Aβ_40_ and Aβ_42_. Nilsson and co-workers demonstrated by isotope-edited IR spectroscopy and solid-state NMR spectroscopy that l- and d-Aβ_16-22_ heptapeptides co-assemble to give rippled β-sheets and showed that the heterochiral assembly is more thermodynamically favorable.^11^ Raskatov and co-workers observed that mixing d-Aβ_42_ with l-Aβ_42_ led to accelerated non-toxic fibril formation and attenuated cytotoxicity by suppressing oligomer formation.^12^ Raskatov subsequently proposed “Aβ chiral inactivation” as a potential therapeutic strategy for Alzheimer's disease.^6,13^ Recently, structures of the rippled β-sheet assembly have been reported. Tycko and Raskatov used solid-state NMR spectroscopy to elucidate ^15^N,^13^C-labeled d,l-Aβ_40_ fibril polymorphs in rippled β-sheets consisting of three different registrations in the hydrogen-bonded antiparallel alignment.^14^ Raskatov and co-workers also reported the X-ray crystallographic structure of a rippled β-sheet formed from a mixture of l- and d-triphenylalanine.^15^ DFT calculations have further supported a model in which heterochiral rippled β-sheets are favored over homochiral pleated β-sheets.^16,17^ From these studies, a theory has emerged in which mixtures of enantiomeric β-sheet peptides are thought to prefer to co-assemble in a heterochiral fashion to form rippled β-sheets, rather than self-assemble in a homochiral fashion to form pleated β-sheets.

In 2004, our laboratory reported that enantiomeric β-sheet pentapeptides strongly prefer to form homochiral pleated β-sheet dimers in CDCl_3_ solution, rather than heterochiral rippled β-sheet dimers, with a selectivity of 3.1–4.2 kcal mol^−1^.^18^ Recently Gellman and co-workers have studied homochiral and heterochiral β-sheet formation in aqueous solution using a β-hairpin model system and have found that peptides containing homochiral peptide strands fold to form β-hairpins, while peptides containing heterochiral peptide strands do not.^19^ Intrigued by the conflicting reports of preferred homochiral and heterochiral β-sheet assembly, we set out to reconcile these findings using a minimal aqueous model system that recapitulates both the edge-to-edge hydrogen-bonding interactions that occur in β-sheet formation and additional face-to-face packing interactions that occur in gel and fibril formation. The model system consists of two well characterized β-sheet peptides derived from Aβ_17–23_ and Aβ_30–36_, peptides 1a and 1b.^20,21^ Peptides 1a and 1b both form tetramers comprising sandwiches of β-sheet dimers. Using NMR spectroscopy, we identify and characterize the different tetramers formed by mixing peptides 1a and 1b with their respective d-enantiomers, *ent*-1a and *ent*-1b. Through these studies, we find that homochiral pairing to form pleated β-sheets is preferred over heterochiral pairing to form rippled β-sheets.

## Results and discussion

### ^1^H NMR spectroscopy shows that mixing peptides 1a and *ent*-1a gives a new assembly

Enantiomerically pure peptide 1a forms a homochiral tetramer in aqueous solution at millimolar concentrations. Peptide 1a is a macrocyclic β-hairpin peptide containing two heptapeptide strands linked by two ^δ^Orn turn units.^22^ The upper strand of peptide 1a is derived from Aβ_17–23_, and the lower strand contains a Hao amino acid flanked by two dipeptides to promote solubility and prevent uncontrolled aggregation.^23^ The tetramer formed by peptide 1a consists of a sandwich of β-sheet dimers. Hydrogen-bonding interactions between the edges of the β-strands stabilize the dimers, and hydrophobic packing of the side chains further stabilizes the tetrameric assembly.
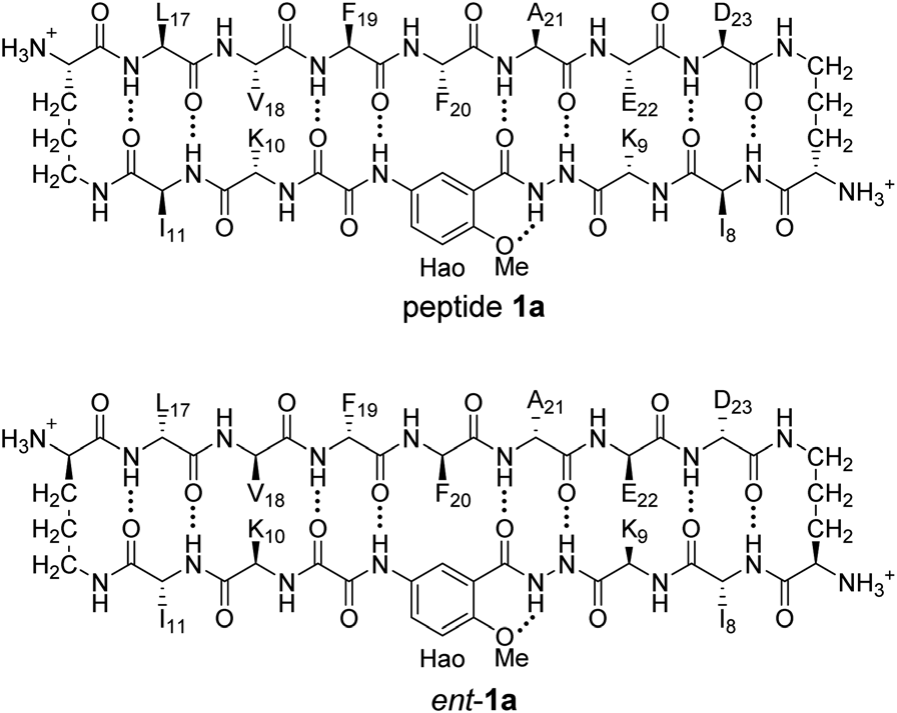


**Fig. 2:**
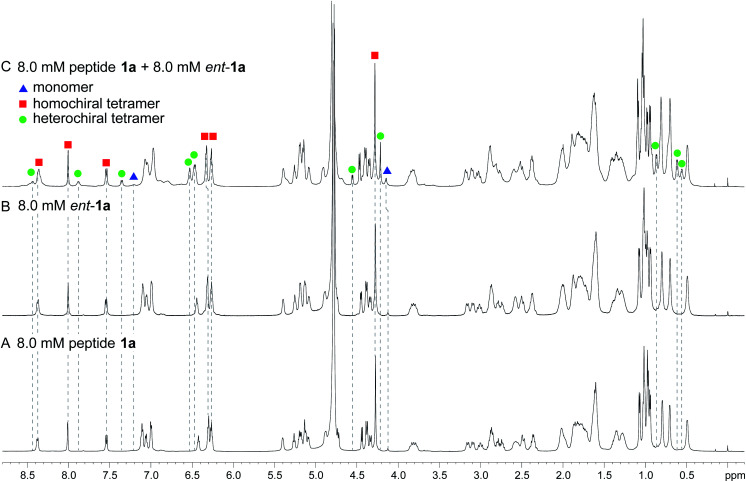
^1^H NMR spectra of (A) 8.0 mM peptide 1a, (B) 8.0 mM peptide *ent*-1a, and (C) 8.0 mM peptide 1a and 8.0 mM peptide *ent*-1a in D_2_O at 600 MHz and 298 K with 0.06 mM DSA as an internal standard.^25^ Dashed lines mark key resonances associated with the monomer, homochiral tetramer, and heterochiral tetramer. These resonances are designated as follows: blue triangle, monomer; red square, homochiral tetramer; green circle, heterochiral tetramer.

The ^1^H NMR spectrum of peptide 1a at 8.0 mM in D_2_O at 298 K displays a predominant set of resonances associated with a homochiral tetramer, and a smaller set of resonances (4%) associated with the monomer. When peptides 1a and *ent*-1a are mixed in equal concentrations (16.0 mM total), new resonances (29%) emerge that were previously unobserved for each enantiopure peptide (Fig. 2). An EXSY experiment at 328 K shows that these new resonances exchange with the homochiral tetramer and monomer and thus correspond to a new heterochiral assembly (Fig. S1–S4†).^24^

### ^1^H,^15^N HSQC and DOSY studies reveal a heterochiral tetramer

HSQC studies using ^15^N isotopic labeling corroborate the formation of the new assembly observed in the 1D ^1^H NMR spectrum. ^1^H,^15^N HSQC experiments give a unique crosspeak for each species containing an ^15^N isotope, readily allowing the identification of the different isotopically labeled species present.^20,21^ We thus prepared an isotopologue of peptide 1a containing an ^15^N label on Phe_20_—peptide 2a—and studied its mixture with peptide *ent*-1a by ^1^H,^15^N HSQC.
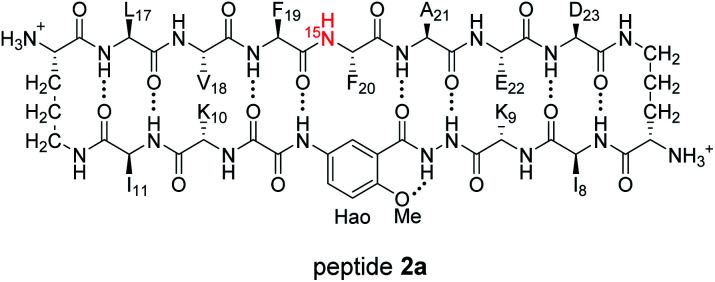


**Fig. 3:**
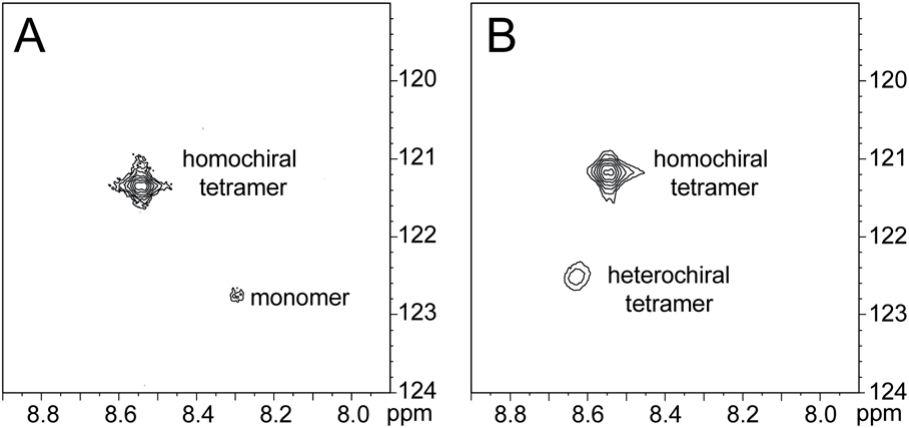
^1^H,^15^N HSQC spectra of (A) 8.0 mM peptide 2a and (B) 8.0 mM peptide 1a and 8.0 mM peptide *ent*-1a in 9 : 1 H_2_O–D_2_O at 500 MHz and 298 K.

The ^1^H,^15^N HSQC spectrum of 8.0 mM peptide 2a in 9 : 1 H_2_O–D_2_O solution shows two crosspeaks—one associated with the homochiral tetramer and the other with the monomer (Fig. 3A).^20^ The tetramer has a crosspeak that appears at 8.56 ppm in the ^1^H dimension and 121.4 ppm in the ^15^N dimension; the monomer has a crosspeak that appears at 8.30 ppm in the ^1^H dimension and 122.8 ppm in the ^15^N dimension. When peptide 2a is mixed with peptide *ent*-1a (16.0 mM total), the crosspeak of the monomer is no longer observed, and a new crosspeak appears at 8.63 ppm in the ^1^H dimension and 122.5 ppm in the ^15^N dimension (Fig. 3B). This crosspeak is not observed in the ^1^H,^15^N HSQC spectrum of the enantiomerically pure peptide 2a and is thus associated with the formation of a heterochiral species. The weaker intensity of this new crosspeak indicates that the homochiral tetramer forms preferentially under the conditions of the experiment.

Diffusion-ordered spectroscopy (DOSY) studies suggest that the new heterochiral species is a tetramer. The DOSY spectrum of 8.0 mM peptide 1a in D_2_O shows a diffusion coefficient of 11.6 ± 0.9 × 10^−11^ m^2^ s^−1^ for the tetramer (Fig. S5†). This value is similar to what we have previously reported for peptide 1a at 8.0 mM and 298 K.^20^ In the DOSY spectrum of the mixture of peptides 1a and *ent*-1a (8.0 mM each), the resonances corresponding to the homochiral tetramer show a diffusion coefficient of 10.7 ± 0.7 × 10^−11^ m^2^ s^−1^, and the resonances corresponding to the heterochiral tetramer show a diffusion coefficient of 10.2 ± 0.7 × 10^−11^ m^2^ s^−1^ (Fig. S6†). The small differences among the diffusion coefficients may reflect transient non-specific interactions among the tetramers at the higher concentration (16.0 mM total) of the mixing experiment leading to a lower diffusion coefficient.^26^

### Supramolecular assembly of the heterochiral tetramer

**Fig. 4:**
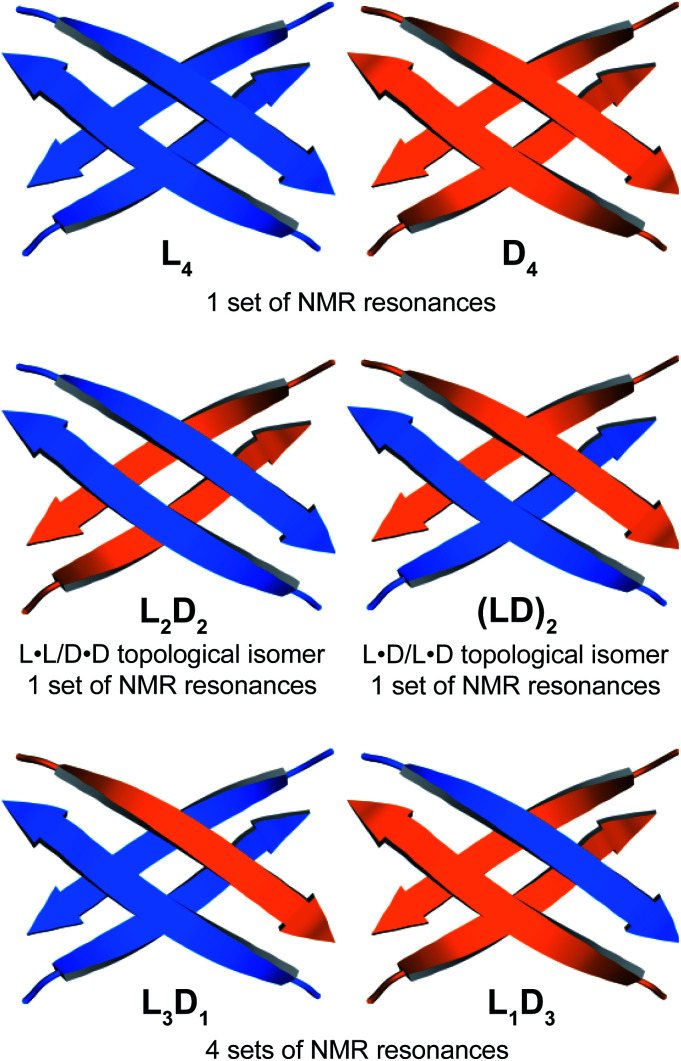
Cartoon illustrations representing the possible combinations of homochiral and heterochiral tetramers formed from a mixture of peptides 1a and *ent*-1a. Macrocyclic peptide 1a is represented by a blue arrow and macrocyclic peptide *ent*-1a is represented by an orange arrow.

Through NOESY studies of peptide 1a, our laboratory previously established that the tetramer formed by peptide 1a consists of sandwiches of β-sheet dimers.^20,21^ The heterochiral tetramer formed by peptides 1a and *ent*-1a can adopt a similar structure, in which two dimers form a sandwich-like tetramer. Peptides 1a and *ent*-1a can come together in two different ways to form heterochiral tetramers in 2 : 2 stoichiometry—either as an L_2_D_2_ topological isomer, in which the sandwich consists of L·L and D·D homochiral dimers, or as an (LD)_2_ topological isomer, in which the sandwich consists of two L·D heterochiral dimers (Fig. 4). The L_2_D_2_ tetramer should give one set of resonances in the ^1^H NMR spectrum, distinct from those of the L_4_ and D_4_ homochiral tetramers, which collectively should give one set of resonances. The (LD)_2_ tetramer should also give one set of resonances in the ^1^H NMR spectrum. Peptides 1a and *ent*-1a could also come together to give heterochiral tetramers in 3 : 1 and 1 : 3 stoichiometry, L_3_D_1_ and L_1_D_3_, which should give four sets of resonances in the ^1^H NMR spectrum. The observation of a single set of new resonances in the ^1^H NMR spectra of the mixture thus indicates the formation of a single heterochiral tetramer with a 2 : 2 stoichiometry as either the L_2_D_2_ or the (LD)_2_ topological isomer.

### ^15^N-edited NOESY studies reveal homochiral dimers within the heterochiral tetramer

**Fig. 5:**
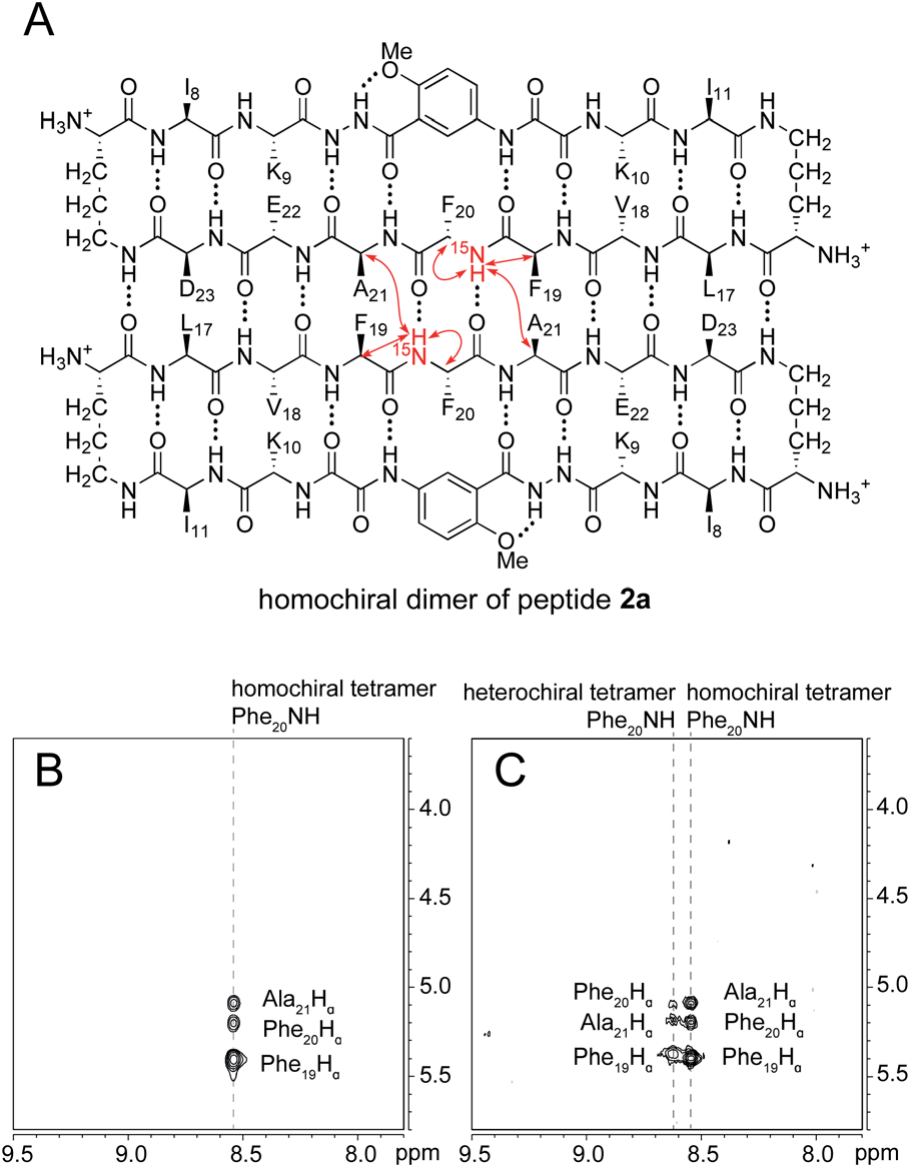
(A) Structure of the homochiral dimer subunit of the tetramer formed from ^15^N-labeled peptide 2a. Red arrows represent NOEs observed in the ^15^N-edited NOESY spectrum. (B) ^15^N-edited NOESY spectrum of 8.0 mM peptide 2a in 9 : 1 H_2_O–D_2_O at 500 MHz and 298 K. (C) ^15^N-edited NOESY spectrum of 8.0 mM peptide 2a and 8.0 mM peptide *ent*-1a in 9 : 1 H_2_O–D_2_O at 500 MHz and 298 K.

The ^15^N-edited NOESY spectrum of ^15^N-labeled enantiomerically pure peptide 2a shows three NOE crosspeaks associated with close contacts in the homochiral tetramer (Fig. 5B). The Phe_20_^15^NH proton of the tetramer shows an interstrand NOE with the Ala_21_ α-proton in its dimerization partner, as well as a strong intrastrand NOE with the Phe_19_ α-proton and a weaker intrastrand NOE with the Phe_20_ α-proton. This pattern of NOEs is characteristic of the proximities observed in β-sheet structure (Fig. 5A).^28,29^

When ^15^N-labeled peptide 2a is mixed with unlabeled peptide *ent*-1a, a new set of weaker NOE crosspeaks associated with the heterochiral tetramer emerges, in addition to the NOE crosspeaks associated with the homochiral tetramer (Fig. 5C). In the set of NOE crosspeaks from the heterochiral tetramer, the Phe_20_^15^NH proton shows an interstrand NOE with the Ala_21_ α-proton in its dimerization partner, as well as a relatively strong intrastrand NOE with the Phe_19_ α-proton and a weaker intrastrand NOE with the Phe_20_ α-proton. Although the observation of a new set of crosspeaks establishes the formation of a heterochiral tetramer, it does not distinguish between the L_2_D_2_ and the (LD)_2_ topological isomers.

**Fig. 6:**
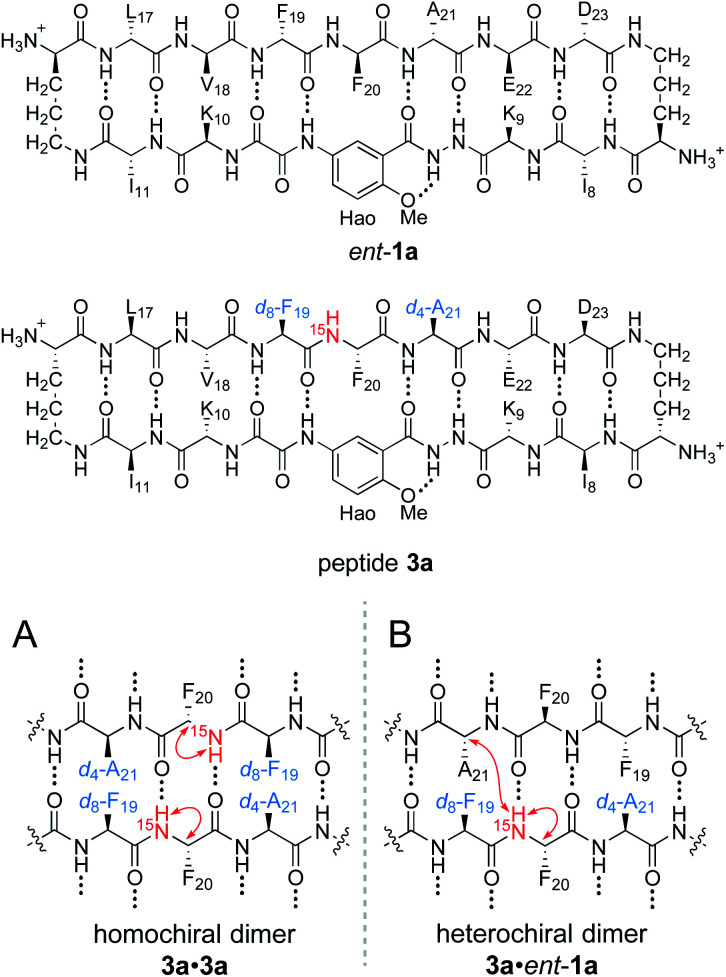
Structures of peptide *ent*-1a and triply labeled peptide 3a. (A) Expected NOEs in the ^15^N-edited NOESY spectrum of a homochiral dimer subunit. (B) Expected NOEs in the ^15^N-edited NOESY spectrum of a heterochiral dimer subunit.

To differentiate between the L_2_D_2_ and the (LD)_2_ topological isomers, we strategically incorporated two deuterated residues (*d*_8_-Phe_19_ and *d*_4_-Ala_21_) into ^15^N-labeled peptide 2a, to create peptide 3a, and we studied its interaction with unlabeled *ent*-1a by ^15^N-edited NOESY experiments. A homochiral dimer in which peptide 3a is paired with itself should not exhibit an interstrand NOE between the ^15^NH proton of Phe_20_ and the α-proton of *d*_4_-Ala_21_, because the α-proton has been replaced with deuterium (Fig. 6A). In contrast, a heterochiral dimer in which peptide 3a is paired with peptide *ent*-1a should exhibit an interstrand NOE between the ^15^NH proton of Phe_20_ in peptide 3a and the α-proton of Ala_21_ in peptide *ent*-1a (Fig. 6B).

**Fig. 7:**
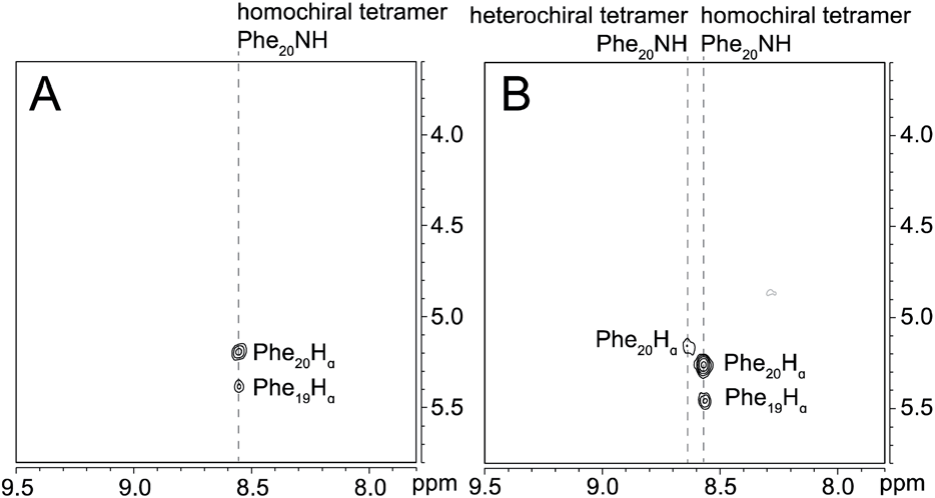
^15^N-Edited NOESY spectra of (A) 8.0 mM triply labeled peptide 3a and (B) 8.0 mM peptide 3a and 8.0 mM unlabeled peptide *ent*-1a in 9 : 1 H_2_O–D_2_O at 500 MHz and 298 K.

^15^N-Edited NOESY studies of the mixture of peptides 3a and *ent*-1a reveal a weak crosspeak associated with a heterochiral tetramer composed of homochiral dimers, in addition to crosspeaks associated with the homochiral tetramer. Enantiomerically pure peptide 3a exhibits the expected intrastrand NOE crosspeak between the ^15^NH proton and the α-proton of Phe_20_ and an unexpected weaker NOE crosspeak to the α-proton of *d*_8_-Phe_19_ (Fig. 7A). This weaker NOE results from incomplete deuterium labeling at the α-position of the *d*_8_-Phe_19_.^30^ In the mixture of peptides 3a and *ent*-1a, a new NOE crosspeak between the ^15^NH proton and the α-proton of Phe_20_ associated with the heterochiral tetramer is observed (Fig. 7B). No additional NOE crosspeaks are observed for the heterochiral tetramer. The presence of only an intrastrand NOE crosspeak in the ^15^N-edited NOESY spectrum of the mixture indicates that peptides 3a and *ent*-1a are not dimerization partners and shows that the heterochiral tetramer is composed of two homochiral dimer subunits.

Collectively the 1D, ^1^H,^15^N HSQC, DOSY, and ^15^N-edited NOESY studies establish that peptides 1a and *ent*-1a preferentially form the L_4_ and D_4_ homochiral tetramers, in addition to a smaller amount of the L_2_D_2_ heterochiral tetramer (Fig. 4). The formation of the L_2_D_2_ heterochiral tetramer rather than the (LD)_2_ heterochiral tetramer demonstrates that even within heterochiral assemblies, enantiomeric β-sheet peptides prefer to self-assemble in a homochiral fashion. Thus, the formation of the L_2_ and D_2_ pleated β-sheets is preferred over the formation of the LD rippled β-sheets.

### ^1^H NMR spectroscopy shows that mixing peptides 1b and *ent*-1b does not result in heterochiral assembly

To further assess the preferences for homochiral or heterochiral assembly using a different β-sheet peptide, we studied the assembly of peptides 1b and *ent*-1b.^20^ Peptide 1b is a homologue of peptide 1a that contains Aβ_30–36_ instead of Aβ_17–23_. We had previously found that peptide 1b also assembles to form a tetramer at millimolar concentrations, albeit with an equilibrium that favors the tetramer less strongly.
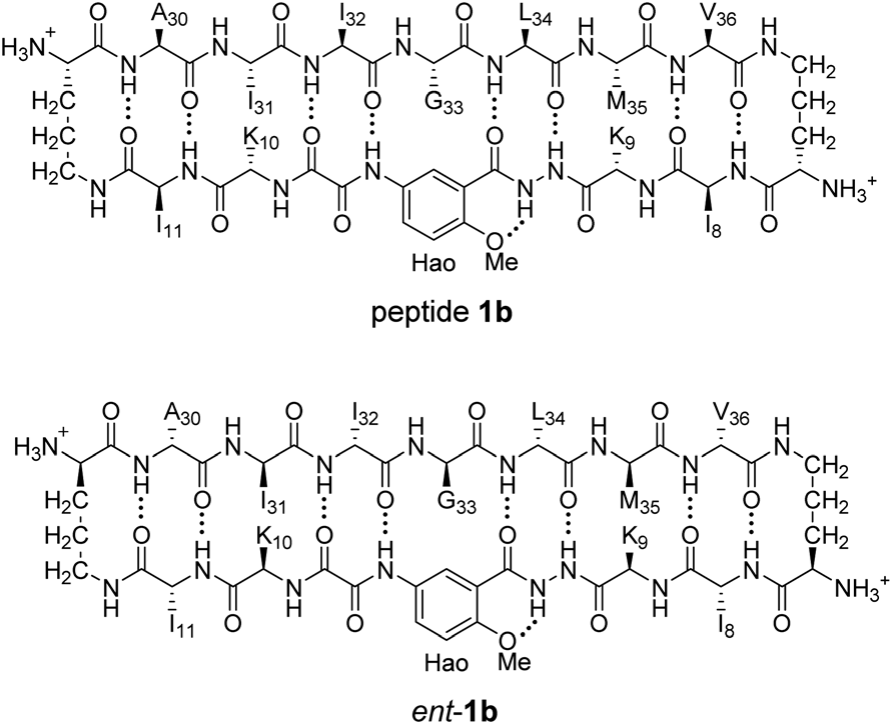


**Fig. 8:**
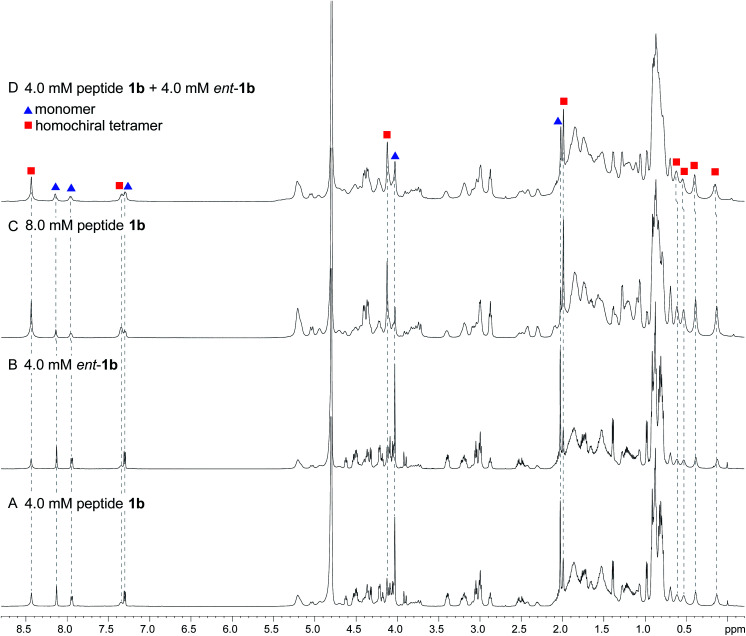
^1^H NMR spectra of (A) 4.0 mM peptide 1b, (B) 4.0 mM peptide *ent*-1b, (C) 8.0 mM peptide 1b, and (D) 4.0 mM peptide 1b and 4.0 mM peptide *ent*-1b in D_2_O at 600 MHz and 298 K with 0.06 mM DSA.^25^ (Spectra were referenced against an external DSA standard.) Dashed lines mark key resonances associated with the monomer and homochiral tetramer. These resonances are labeled as follows: blue triangle, monomer; red square, homochiral tetramer.

When peptide 1b is mixed with peptide *ent*-1b, no evidence of heterochiral tetramer formation is observed. The ^1^H NMR spectrum of peptide 1b at 4.0 mM in D_2_O at 298 K displays sets of resonances associated with both the monomer and the homochiral tetramer (Fig. 8A). The 4.0 mM ^1^H NMR spectrum of peptide *ent*-1b is identical to that of peptide 1b (Fig. 8B). At 8.0 mM, the spectrum of peptide 1b displays a shift in equilibrium toward the tetramer (Fig. 8C). The spectrum broadens slightly, suggesting exchange between the monomer and tetramer on an intermediate timescale (*ca.* 10^−1^ s) or additional non-specific interactions.

When peptides 1b and *ent*-1b are mixed (4.0 mM of each), no new peaks form, and only peaks associated with the monomer and homochiral tetramer are observed (Fig. 8D). As with the 8.0 mM spectrum of peptide 1b, there is slight broadening of the spectrum of the mixture of peptides 1b and *ent*-1b, suggesting exchange on an intermediate timescale or additional non-specific interactions.

The DOSY spectrum of 1.0 mM peptide 1b in D_2_O shows a monomer with a diffusion coefficient of 19.5 ± 0.7 × 10^−11^ m^2^ s^−1^ (Fig. S7†). This value is similar to that which we have previously reported for peptide 1b at 1.0 mM and 298 K.^20^ At 4.0 mM, an additional set of smaller resonances associated with the homochiral tetramer appears (Fig. S8†); the monomer shows a diffusion coefficient of 17.7 ± 0.6 × 10^−11^ m^2^ s^−1^, and the tetramer shows a diffusion coefficient of 13.0 ± 0.4 × 10^−11^ m^2^ s^−1^. The decrease in diffusion coefficient of the monomer, as well as the somewhat higher than expected diffusion coefficient of the tetramer—typically about 0.6× that of the monomer,^26^*ca.* 12 × 10^−11^ m^2^ s^−1^—suggest intermediate exchange between the monomer and the tetramer on the 75 ms time scale of the DOSY experiment.

When the concentration of peptide 1b is doubled to 8.0 mM, the resonances associated with the homochiral tetramer predominate (Fig. S9†); the monomer shows a diffusion coefficient of 15.9 ± 0.7 × 10^−11^ m^2^ s^−1^, and the tetramer shows a diffusion coefficient of 12.5 ± 0.4 × 10^−11^ m^2^ s^−1^. The further decrease in diffusion coefficient of the monomer is consistent with intermediate exchange. The DOSY spectrum of the mixture of peptides 1b and *ent*-1b (4.0 mM of each) shows diffusion coefficients of the monomer and tetramer of 14.4 ± 1.1 × 10^−11^ m^2^ s^−1^ and 10.7 ± 0.8 × 10^−11^ m^2^ s^−1^, respectively (Fig. S10†). The low value of the monomer is consistent with intermediate exchange between the monomer and the tetramer. The value of the tetramer is somewhat lower than expected, suggesting additional transient non-specific interactions among the tetramers.^26,27^ The absence of any additional new peaks in the spectrum of the mixture, not present in the spectra of the enantiomerically pure peptides, provides good evidence that mixing peptides 1b and *ent*-1b does not result in any detectable heterochiral assembly.

^1^H,^15^N HSQC studies corroborate the presence of only monomer and homochiral tetramer in the mixture of peptides 1b and *ent*-1b. To identify and confirm the monomer and homochiral tetramer by ^1^H,^15^N HSQC, we prepared an isotopologue of peptide 1b containing an ^15^N label on Gly_33_—peptide 2b—and studied its mixture with peptide *ent*-1b. We studied increasing concentrations of enantiomerically pure peptide 2b (1.0 mM, 4.0 mM, and 8.0 mM), and compared the crosspeaks to those found in the mixture of peptides 2b and *ent*-1b (8.0 mM total).
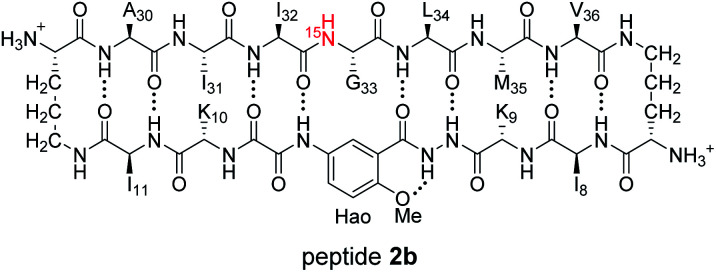


**Fig. 9:**
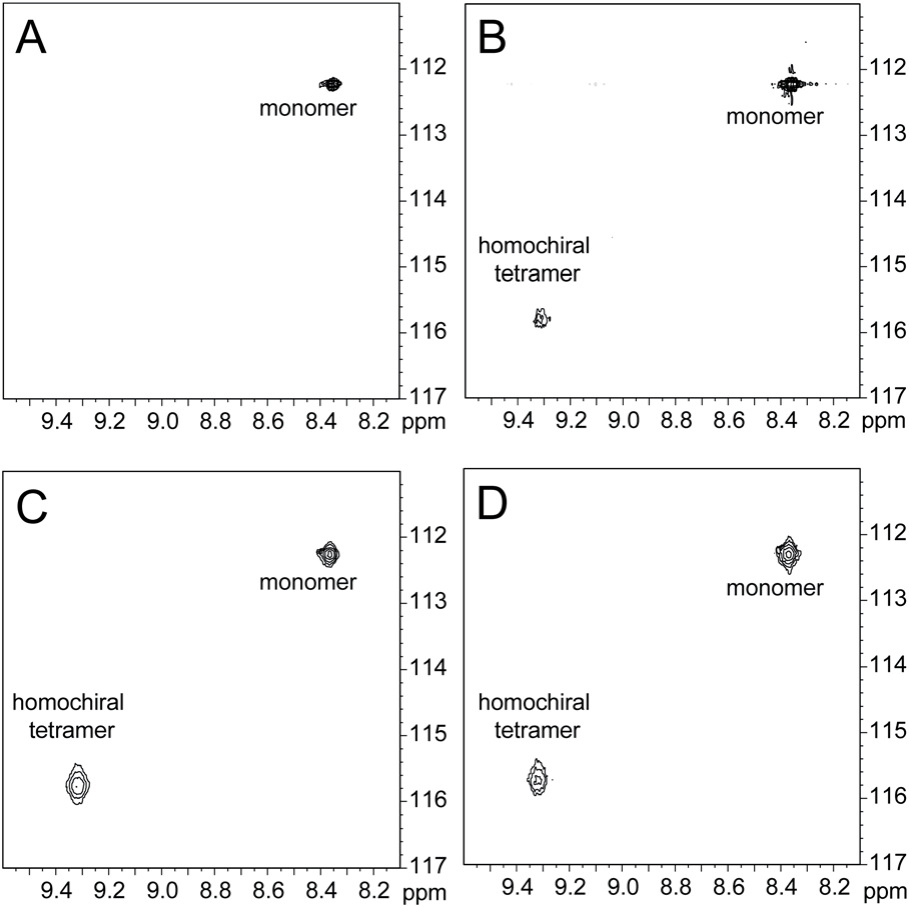
^1^H,^15^N HSQC spectra of (A) 1.0 mM peptide 2b, (B) 4.0 mM peptide 2b, (C) 8.0 mM peptide 2b, and (D) 4.0 mM peptide 2b and 4.0 mM peptide *ent*-1b in 9 : 1 H_2_O–D_2_O at 500 MHz and 298 K.

The ^1^H,^15^N HSQC spectrum of 1.0 mM peptide 2b in 9 : 1 H_2_O–D_2_O solution shows only a crosspeak associated with the monomer, at 8.35 ppm in the ^1^H dimension and 112.2 ppm in the ^15^N dimension (Fig. 9A). At 4.0 mM peptide 2b, the monomer is still present and a crosspeak associated with the homochiral tetramer appears 115.8 ppm in the ^15^N dimension and 9.31 ppm in the ^1^H dimension (Fig. 9B).^20^ When the concentration of peptide 2b is doubled to 8.0 mM, the relative intensity of the tetramer crosspeak increases (Fig. 9C). When peptide 2b is mixed with peptide *ent*-1b (4.0 mM each), crosspeaks associated with the monomer and homochiral tetramer are still present and no new crosspeaks are observed (Fig. 9D). The lack of new crosspeaks further establishes that enantiomeric β-sheet peptides prefer to self-assemble in a homochiral fashion.

## Conclusion

The solution-phase NMR studies of Aβ-derived peptides 1a and 1b and the corresponding enantiomers and isotopologues provide further evidence that enantiomeric β-sheet peptides prefer to self-assemble in a homochiral fashion. These studies recapitulate our laboratory's findings that small β-sheet peptides strongly prefer to form homochiral dimers in chloroform solution,^18^ as well as Gellman's findings in homo- and heterochiral β-hairpin systems.^19^ How then, do we reconcile these findings with the findings of other researchers where heterochiral assembly is preferred?

In the studies of Schneider, Nilsson, Raskatov, and Tycko described in the introduction, heterochiral assembly occurs in the solid or gel state.^5–17^ Heterochiral assembly in the solid state is driven heavily by the packing of molecules, which in addition to edge-to-edge hydrogen bonding, drives the formation of fibrils and crystal lattices. These packing interactions involve not just the side chains within individual β-sheets, but also the packing of β-sheets together. Heterochiral packing is generally preferred over homochiral packing in the crystal state, which leads to denser solids and a preference for racemic crystal formation—a phenomenon known as “Wallach's rule”.^31–33^ Thus, it appears that packing in the solid state may drive the formation of heterochiral mixtures of β-sheet peptides, and in some cases the formation of rippled β-sheets. In the solution phase, where crystal packing forces are absent, rippled β-sheet formation is strongly disfavored. Thus, no evidence of heterochiral pairing to form rippled β-sheets is observed with peptides 1a and 1b and the corresponding enantiomers.

## Data availability

The data supporting this article are available in the ESI.†

## Author contributions

Xingyue Li synthesized the peptides, performed the experiments, analyzed the results, and prepared the manuscript with James S. Nowick. Stephanie E. Rios assisted with the peptide synthesis. James S. Nowick supervised the project and assisted in the experimental design and writing of the manuscript.

## Conflicts of interest

The authors declare no conflicts of interest.

## Supplementary Material

SC-013-D2SC02080G-s001
