# Supplementary material for: Enantiomeric β-sheet peptides from Aβ form homochiral pleated β-sheets rather than heterochiral rippled β-sheets
Source: Chem Sci. 2022 May 31;13(26):7739–46. doi: 10.1039/d2sc02080g (PMC9258340; doi:10.1039/d2sc02080g)
Supplement: SC-013-D2SC02080G-s001 [file SC-013-D2SC02080G-s001.pdf]

Electronic Supplementary Material (ESI) for Chemical Science.
This journal is © The Royal Society of Chemistry 2022

Supporting information for:

Enantiomeric β-Sheet Peptides from Aβ Form Homochiral Pleated β-Sheets Rather than Heterochiral Rippled β-Sheets

Xingyue Li[a], Stephanie E. Rios[a], and James S. Nowick*[a,b]
[a]Department of Chemistry, University of California, Irvine
[b]Department of Pharmaceutical Sciences, University of California, Irvine
Irvine, California 92697-2025, United States

Table of Contents

Supplementary information S3
Figure S1 .... S3
Figure S2 .... S4
Figure S3 .... S5
Figure S4 .... S6
Supplementary Table 1 .... S7
Figure S5 .... S7
Figure S6 .... S8
Supplementary Table 2 .... S9
Figure S7 .... S9
Figure S8 .... S10
Figure S9 .... S11
Figure S10 .... S12
Figure S11 .... S13
Materials and Methods S14
General information S14
Chemicals and Supplies S14
Instrumentation S14
Synthesis of peptides S15
Peptide synthesis procedure. S17
Synthesis of Fmoc-protected amino acids S18
Fmoc-protection procedure. S18
NMR spectroscopy of peptides S20
Sample preparation, data collection, and data processing S20

References S23

Characterization Data S24

NMR characterization of Fmoc-protected amino acids S24

^{1}H NMR, and ^{13}C NMR spectra of Fmoc-d_4-Ala-OH S24

^{1}H NMR, and ^{13}C NMR spectra of Fmoc-d_8-Phe-OH S26

NMR spectroscopic studies of β-hairpin peptides S28

^{1}H NMR, TOCSY, and NOESY spectra of 8.0 mM peptide **1a** S28

^{1}H NMR, TOCSY, and NOESY spectra of peptides **1a** and *ent*-**1a** (8.0 mM each) S34

^{1}H NMR, TOCSY, and NOESY spectra of peptides **3a** and *ent*-**1a** (8.0 mM each) S41

^{1}H NMR, TOCSY, and NOESY spectra of 4.0 mM peptide **1b** S47

^{1}H NMR, TOCSY, and NOESY spectra of peptides **1b** and *ent*-**1b** (4.0 mM each) S53

Analytical HPLC trace and MALDI mass spectrum of peptides S59

Analytical HPLC trace and MALDI mass spectrum of peptide **1a** S59

Analytical HPLC trace and MALDI mass spectrum of peptide *ent*-**1a** S61

Analytical HPLC trace and MALDI mass spectrum of peptide **2a** S63

Analytical HPLC trace and MALDI mass spectrum of peptide **3a** S65

Analytical HPLC trace and MALDI mass spectrum of peptide **1b** S67

Analytical HPLC trace and MALDI mass spectrum of peptide *ent*-**1b** S69

Analytical HPLC trace and MALDI mass spectrum of peptide **2b** S71

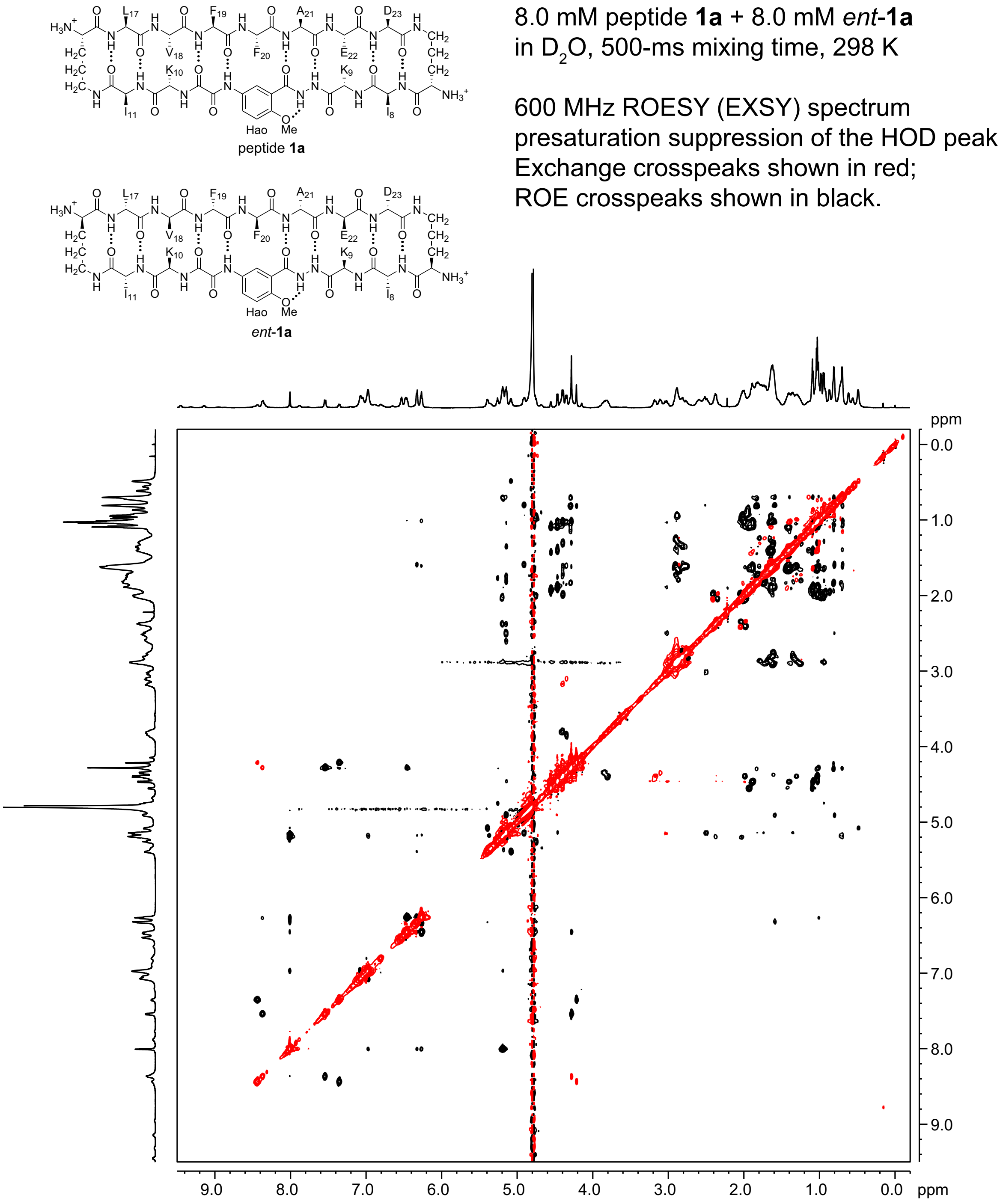

Figure S1. 2D ROESY (EXSY) spectrum of 8.0 mM peptide **1a** and 8.0 mM peptide *ent*-**1a** in D_2O at 600 MHz and 298 K, with 0.06 mM DSA[1]. Residual HOD was suppressed by presaturation of the water peak. A mixing time of 500-ms was used to acquire the spectrum. The two phases of the spectrum are colored with black (ROE crosspeaks) and red (exchange crosspeaks).

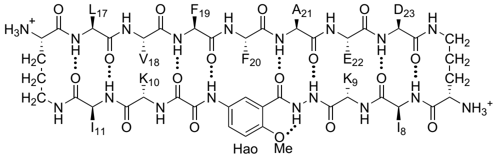

peptide **1a**

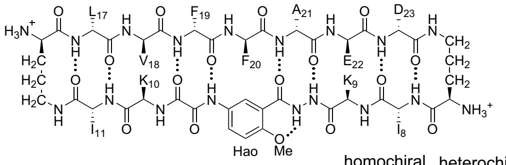

ent-**1a**

8.0 mM peptide **1a** + 8.0 mM *ent*-**1a** in D_2O, 500-ms mixing time, 298 K

600 MHz ROESY (EXSY) spectrum
presaturation suppression of the HOD peak
Exchange crosspeaks shown in red;
ROE crosspeaks shown in black.

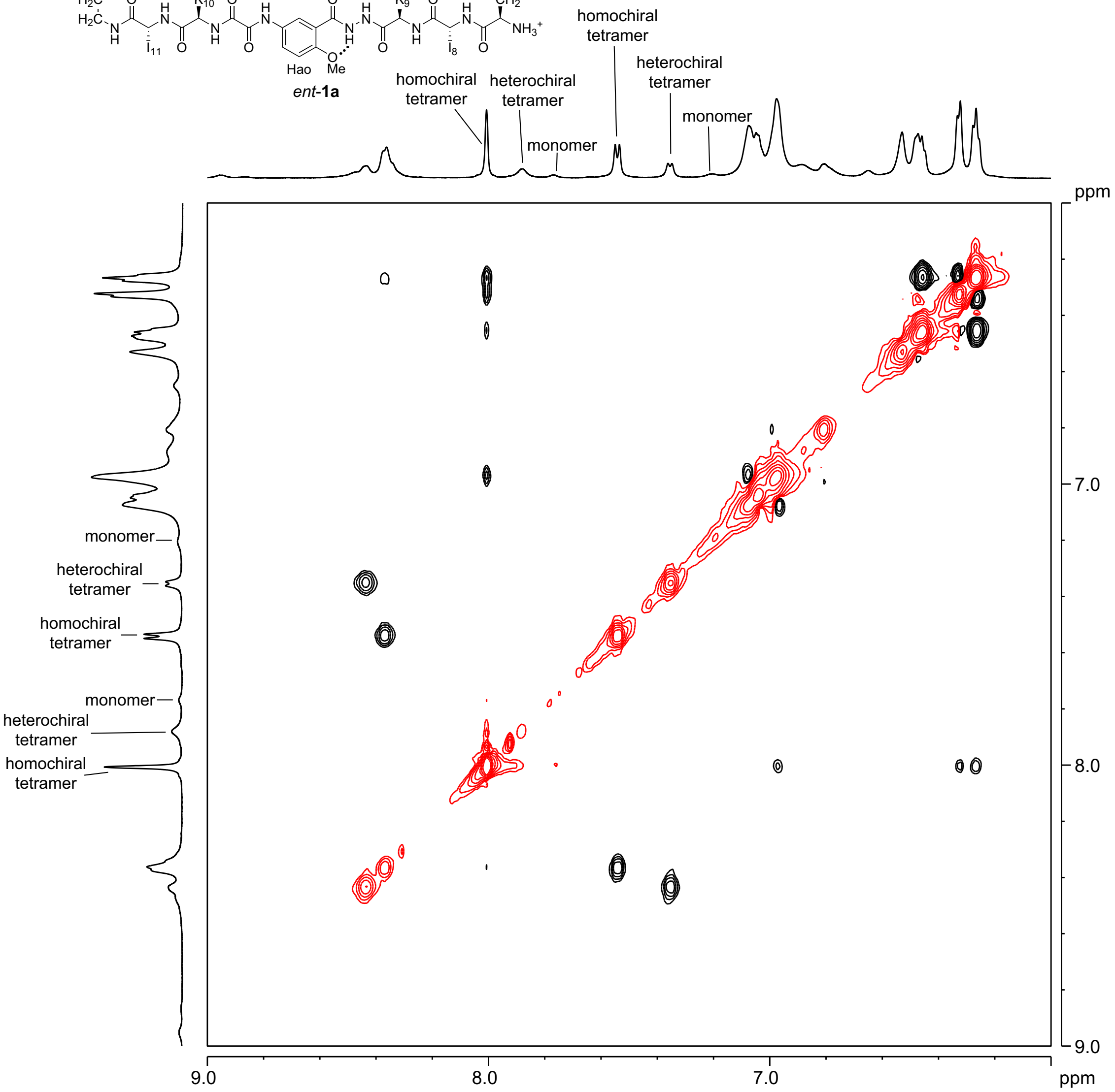

Figure S2. Expansion of Figure S1. No crosspeaks associated with exchange are observed at 298 K.

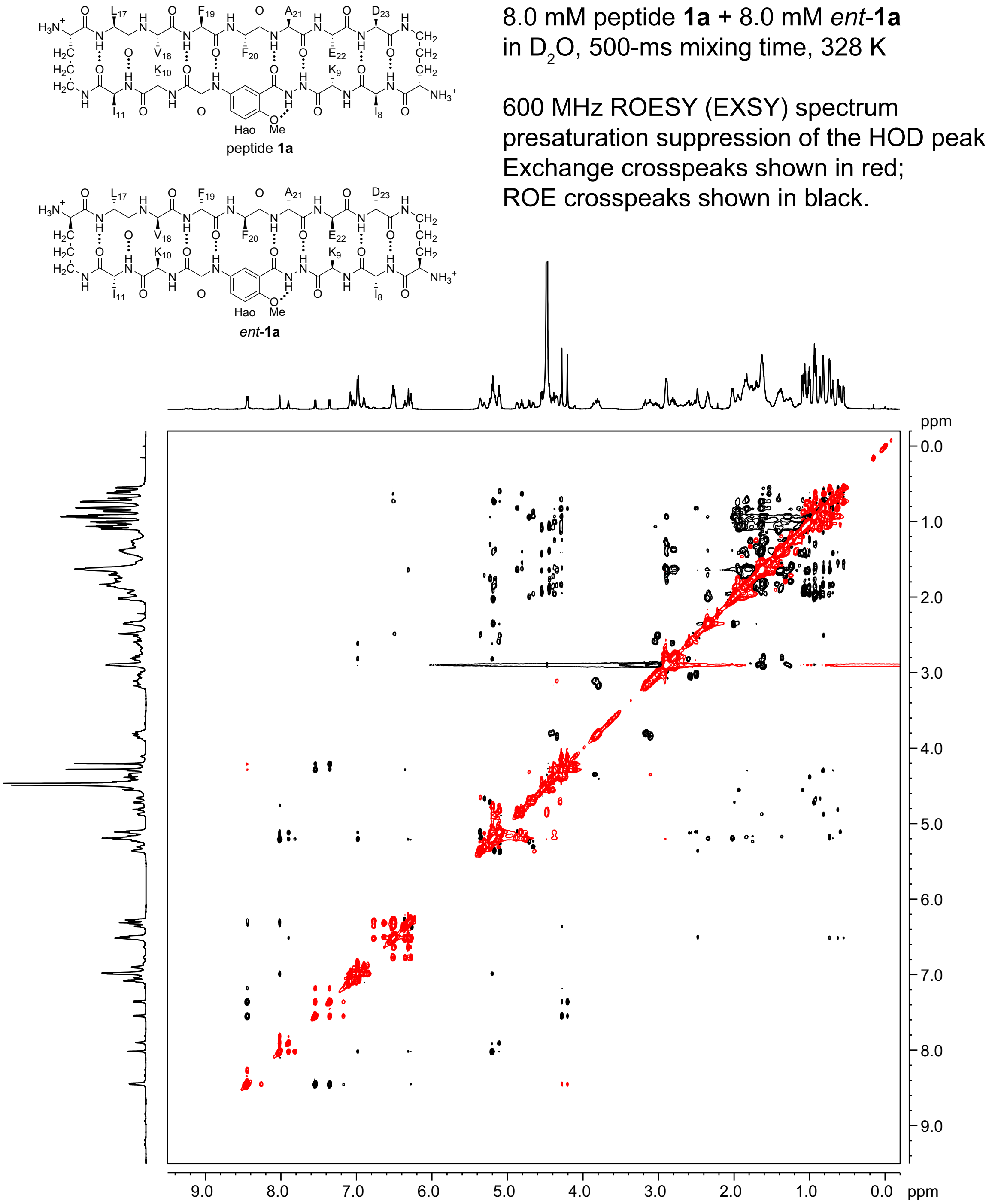

Figure S3. 2D ROESY (EXSY) spectrum of 8.0 mM peptide **1a** and 8.0 mM peptide *ent*-**1a** in D_2O at 600 MHz and 328 K, with 0.06 mM DSA[1]. Residual HOD was suppressed by presaturation of the water peak. A mixing time of 500-ms was used to acquire the spectrum. The two phases of the spectrum are colored with black (ROE crosspeaks) and red (exchange crosspeaks).

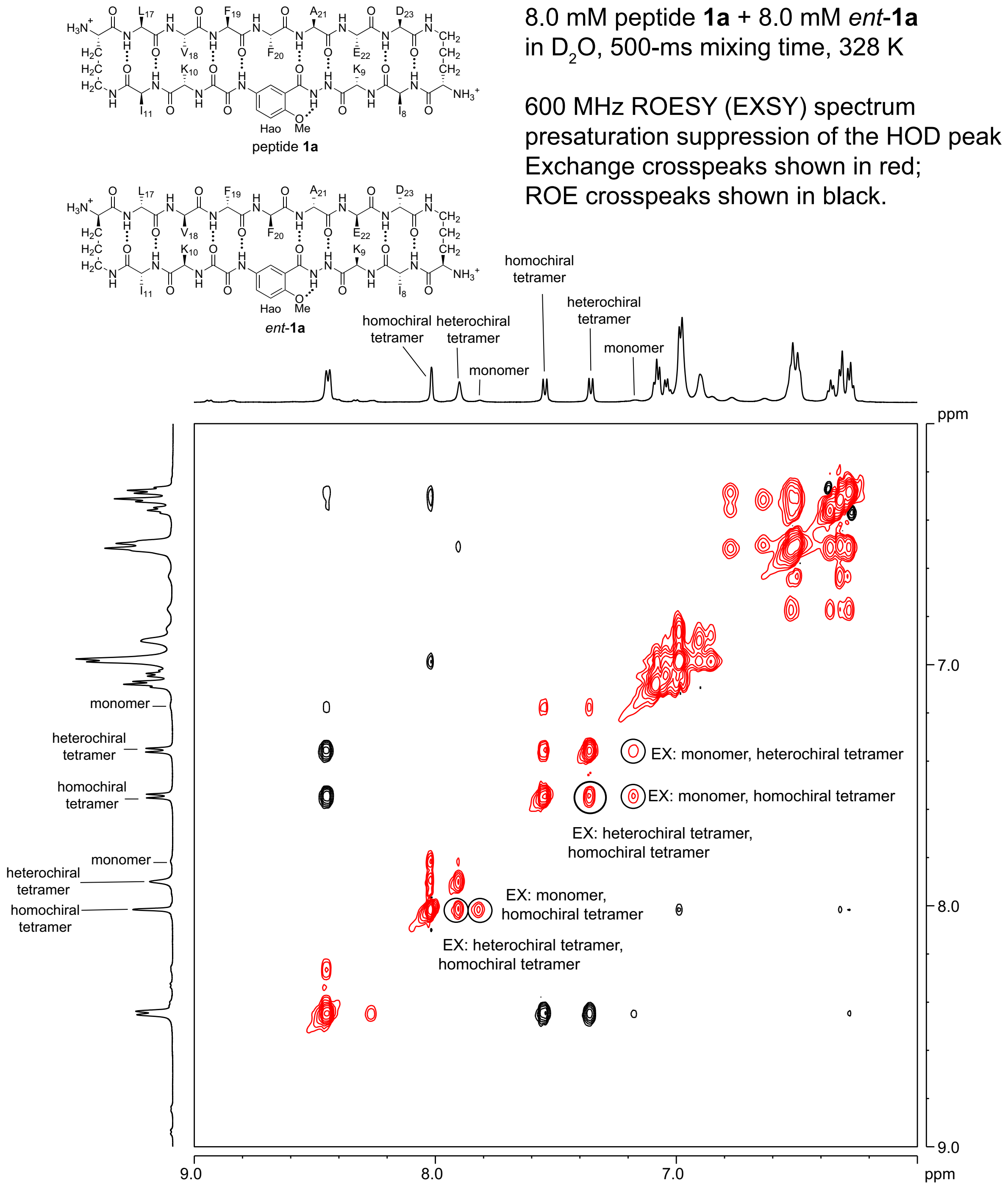

Figure S4. Expansion of Figure S3. Exchange (EX) between different species is shown with labeled representative crosspeaks.

Table S1. Diffusion coefficients (*D*) of peptides 1a and *ent*-1a in D_2O at 298 K

	MW Monomer (Da)	MW Tetramer (Da)	Concentration (mM)	D ($\times 10^{-11}$ m^2/s)	Oligomer State
Peptide **1a**	1767	7068	8.0	11.6 ± 0.9	homochiral tetramer
Peptides **1a** + *ent*-**1a**	1767	7068	8.0 + 8.0	10.7 ± 0.7<br>10.2 ± 0.7	homochiral tetramer<br>heterochiral tetramer

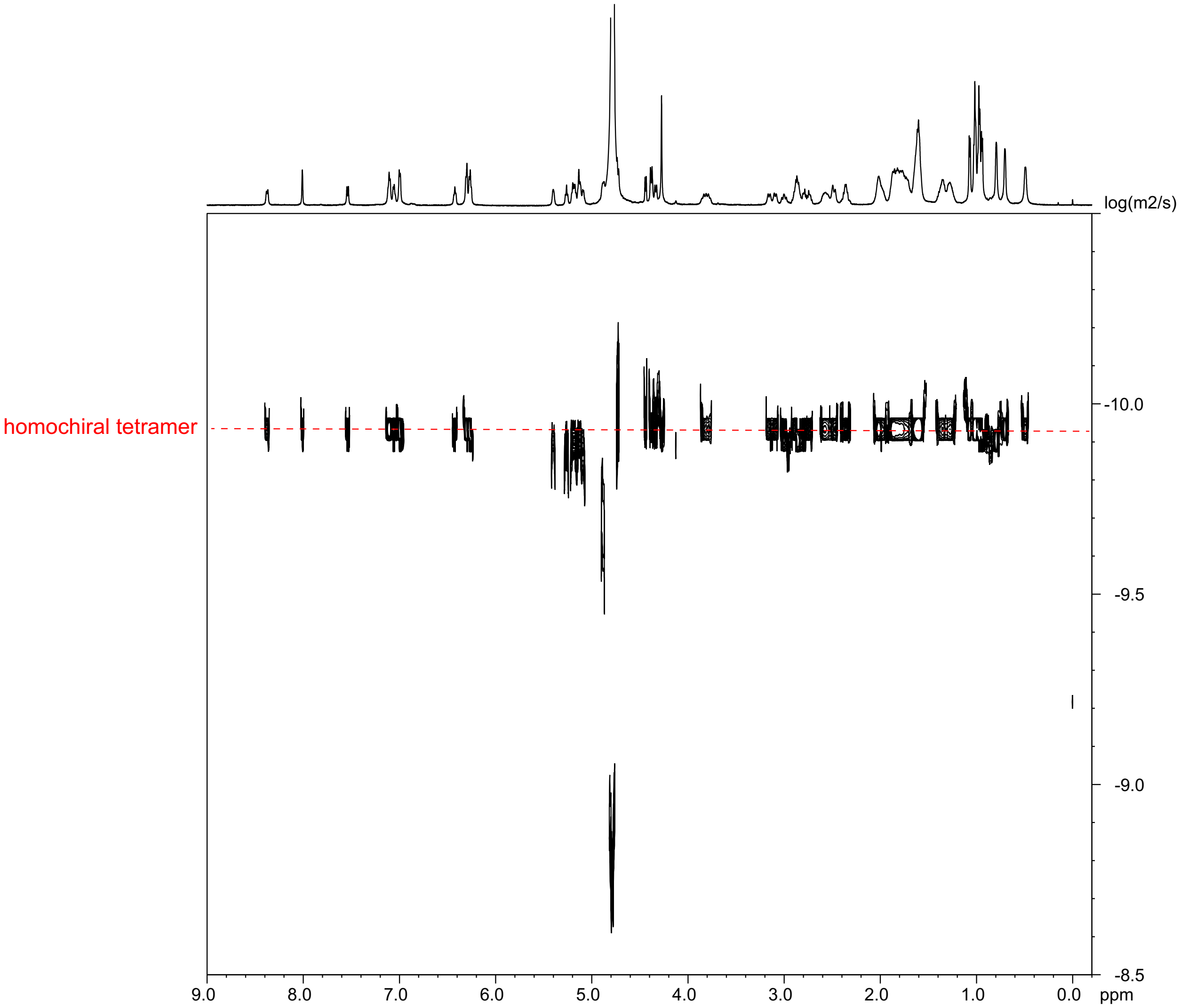

Figure S5. DOSY NMR spectrum of 8.0 mM peptide **1a** in D_2O at 600 MHz and 298 K with 0.06 mM DSA[1]. The homochiral tetramer (red) shows a diffusion coefficient of 11.6 ± 0.9 x 10^{-11} m^2/s. The HOD peak is calibrated to 19.0 x 10^{-10} m^2/s.[2]

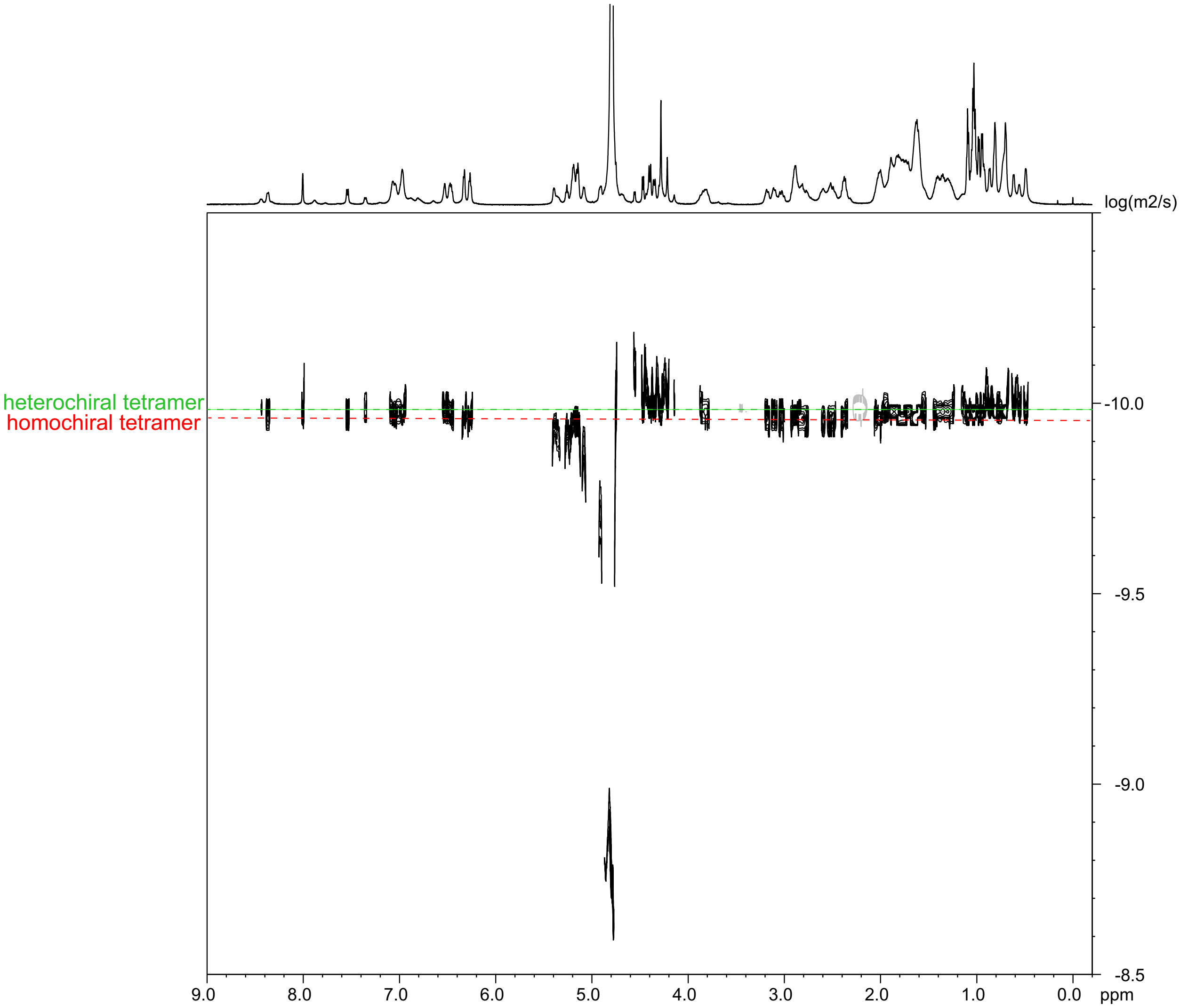

Figure S6. DOSY NMR spectrum of 8.0 mM peptide **1a** and 8.0 mM peptide *ent*-**1a** in D_2O at 600 MHz and 298 K with 0.06 mM DSA[1]. The heterochiral tetramer (green) shows a diffusion coefficient of $10.2 \pm 0.7 \times 10^{-11}$ m^2/s. The homochiral tetramer (red) shows a diffusion coefficient of $10.7 \pm 0.7 \times 10^{-11}$ m^2/s. The HOD peak is calibrated to 19.0×10^{-10} m^2/s.[2]

Table S2. Diffusion coefficients (*D*) of peptides 1b and *ent*-1b in D_2O at 298 K

	MW Monomer (Da)	MW Tetramer (Da)	Concentration (mM)	D ($\times 10^{-11}$ m^2/s)	Oligomer State
Peptide **1b**	1643	6572	1.0	19.5 ± 0.7	monomer
			4.0	17.7 ± 0.6	monomer
				13.0 ± 0.4	homochiral tetramer
			8.0	15.9 ± 0.7	monomer
				12.5 ± 0.4	homochiral tetramer
Peptides **1b** + *ent*-**1b**	1643	6572	4.0 + 4.0	14.4 ± 1.1	monomer
				10.7 ± 0.8	homochiral tetramer

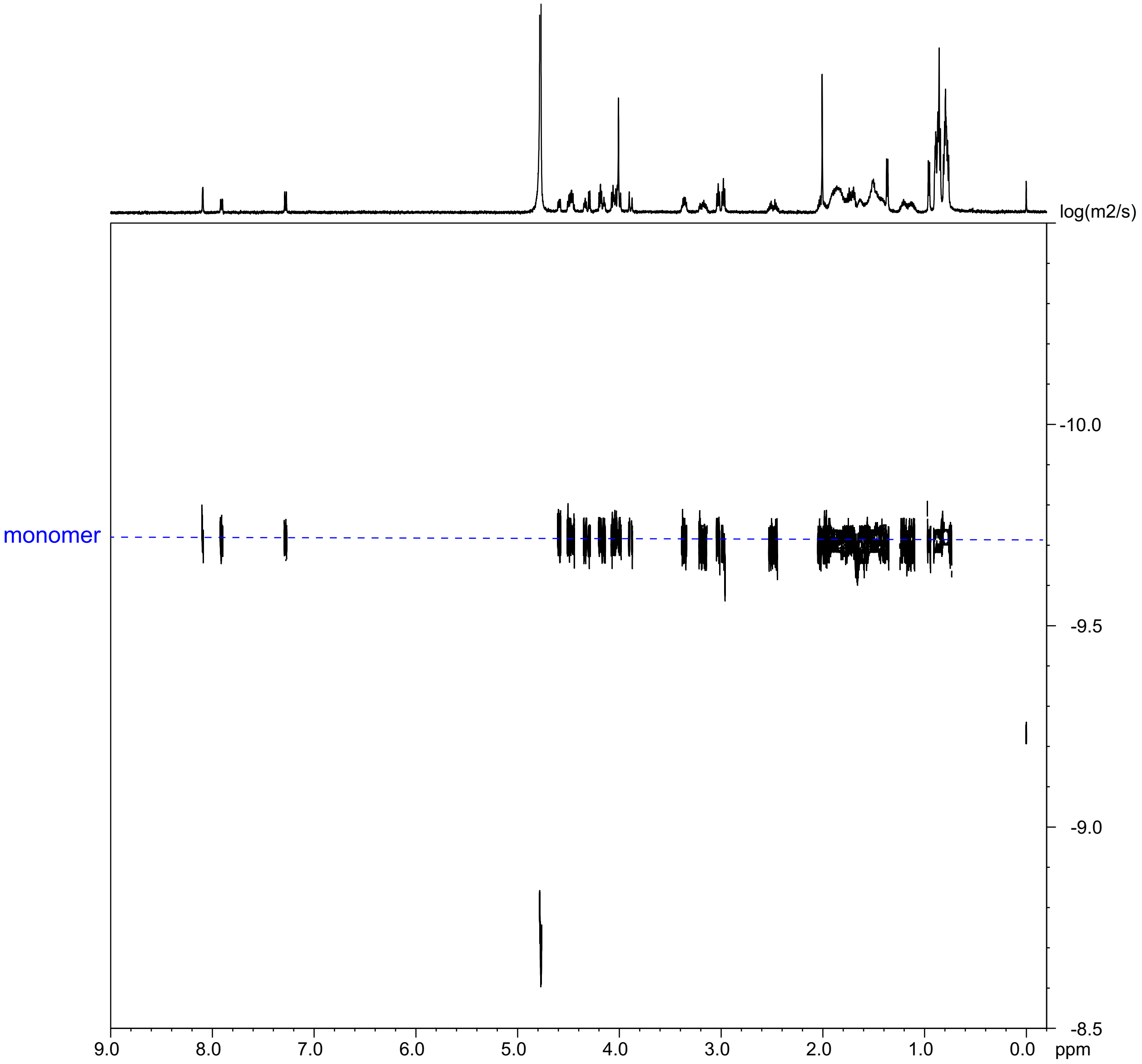

Figure S7. DOSY NMR spectrum of 1.0 mM peptide **1b** in D_2O at 600 MHz and 298 K with 0.06 mM DSA[1]. The monomer (blue) shows a diffusion coefficient of 19.5 ± 0.7 x 10^{-11} m^2/s. The HOD peak is calibrated to 19.0 x 10^{-10} m^2/s.[2]

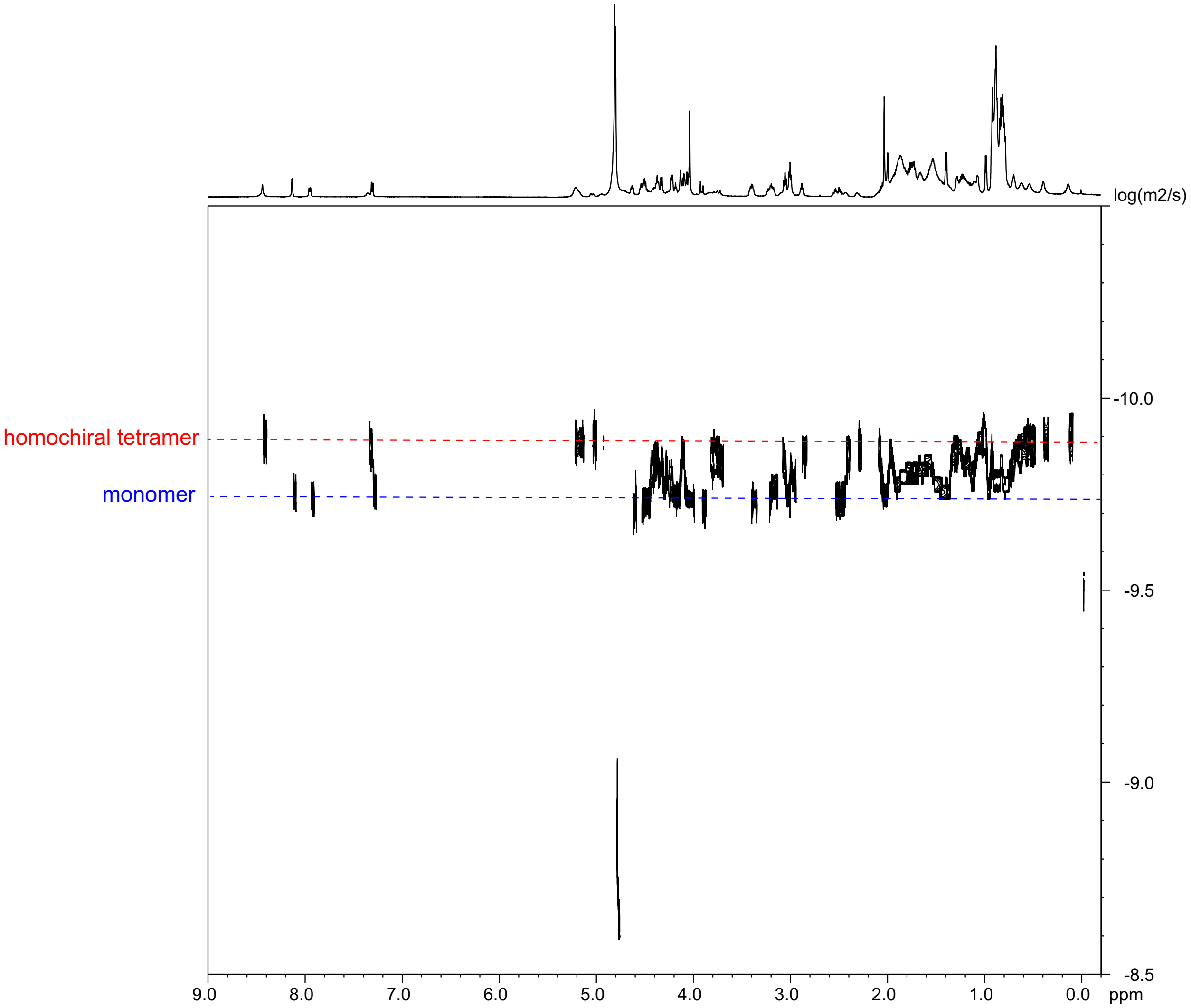

Figure S8. DOSY NMR spectrum of 4.0 mM peptide **1b** in D_2O at 600 MHz and 298 K with 0.06 mM DSA[1]. The homochiral tetramer (red) shows a diffusion coefficient of $13.0 \pm 0.4 \times 10^{-11}$ m^2/s. The monomer (blue) shows a diffusion coefficient of $17.7 \pm 0.6 \times 10^{-11}$ m^2/s. The HOD peak is calibrated to 19.0×10^{-10} m^2/s.[2]

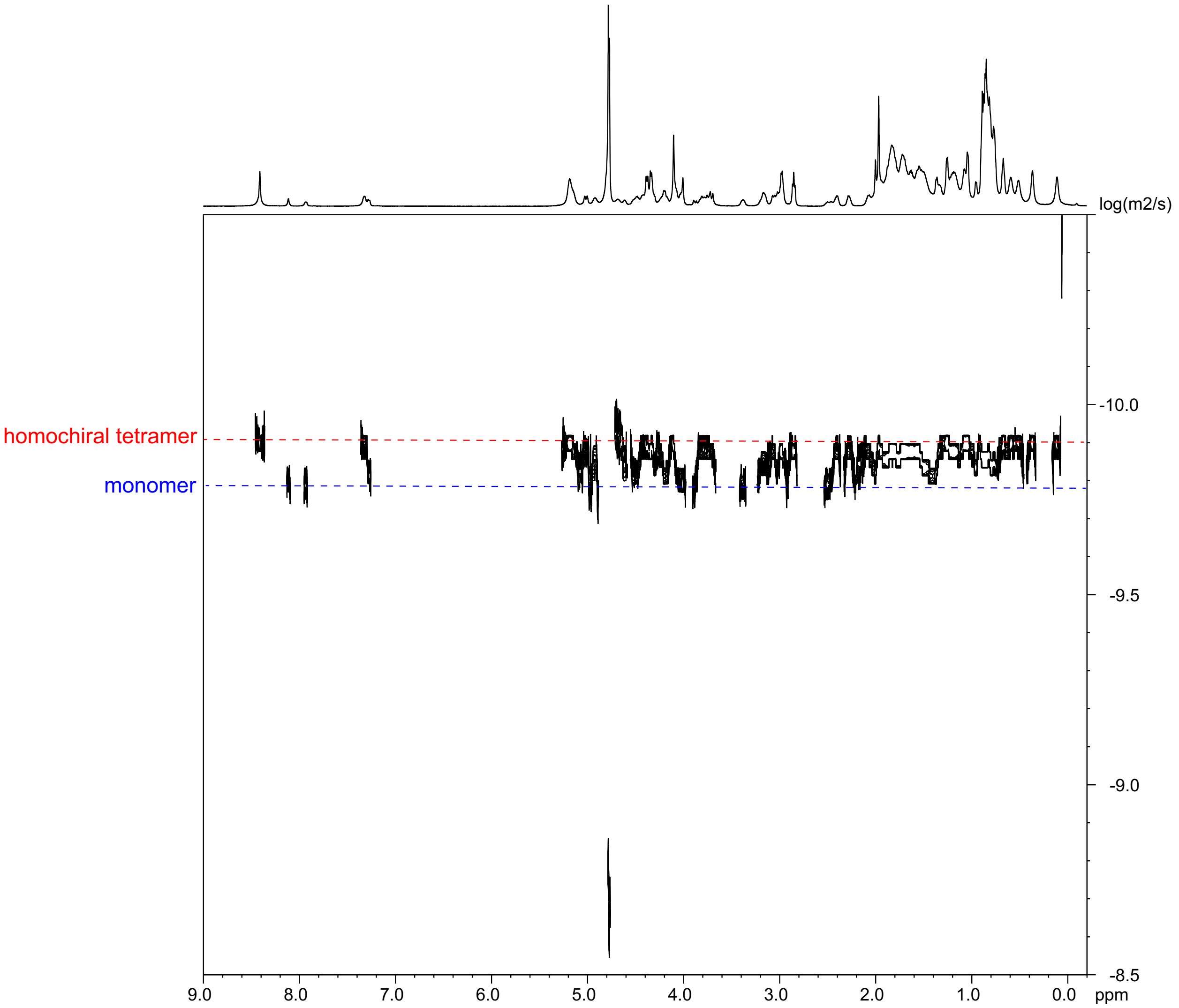

Figure S9. DOSY NMR spectrum of 8.0 mM peptide **1b** in D_2O at 600 MHz and 298 K with 0.06 mM DSA[1]. The homochiral tetramer (red) shows a diffusion coefficient of $12.5 \pm 0.4 \times 10^{-11}$ m^2/s. The monomer (blue) shows a diffusion coefficient of $15.9 \pm 0.7 \times 10^{-11}$ m^2/s. The HOD peak is calibrated to 19.0×10^{-10} m^2/s.[2]

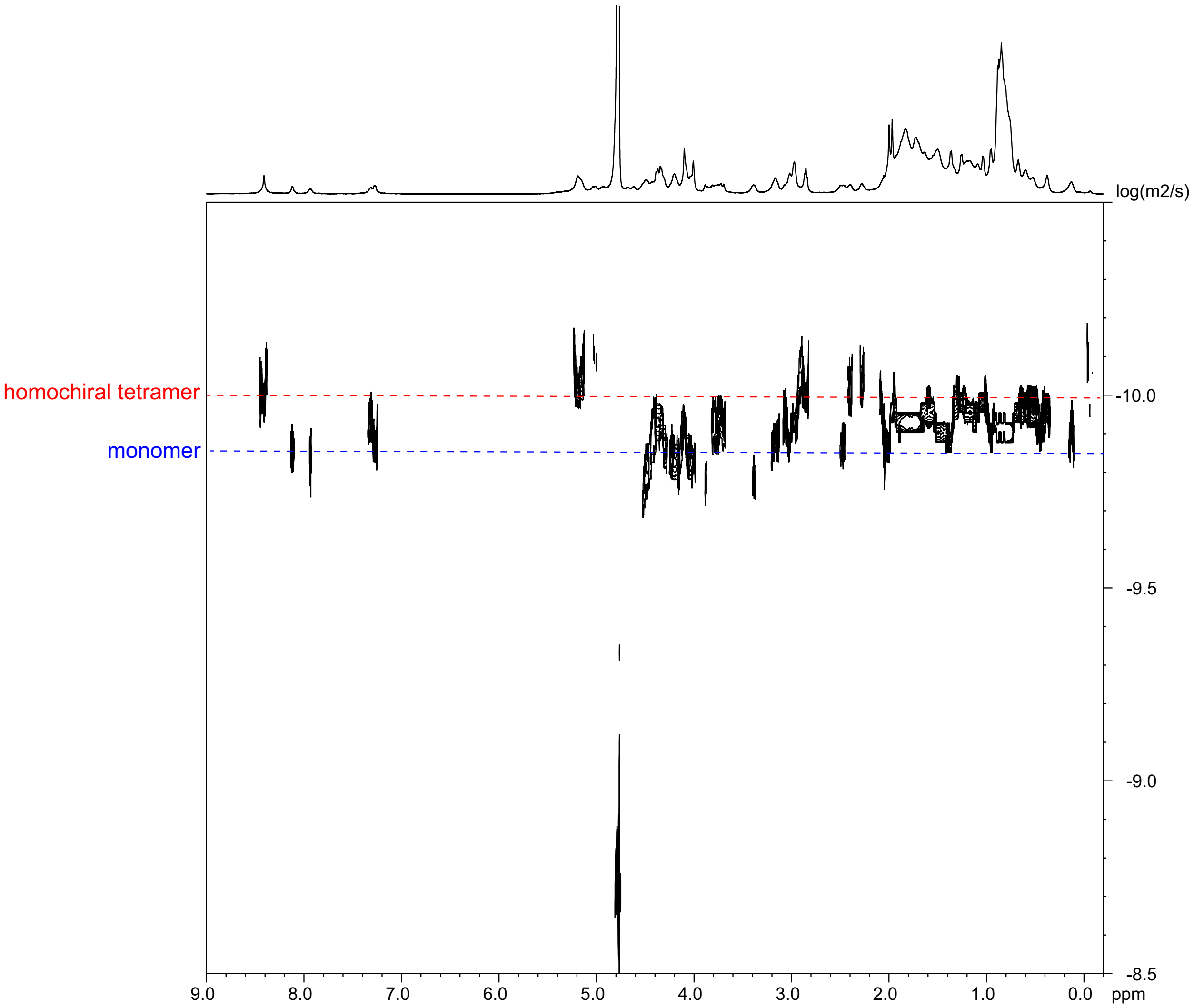

Figure S10. DOSY NMR spectrum of 4.0 mM peptide **1b** and 4.0 mM peptide *ent*-**1b** in D_2O at 600 MHz and 298 K with 0.06 mM DSA[1]. The homochiral tetramer (red) shows a diffusion coefficient of 10.7 ± 0.8 x 10^{-11} m^2/s. The monomer (blue) shows a diffusion coefficient of 14.4 ± 1.1 x 10^{-11} m^2/s. The HOD peak is calibrated to 19.0 x 10^{-10} m^2/s.[2]

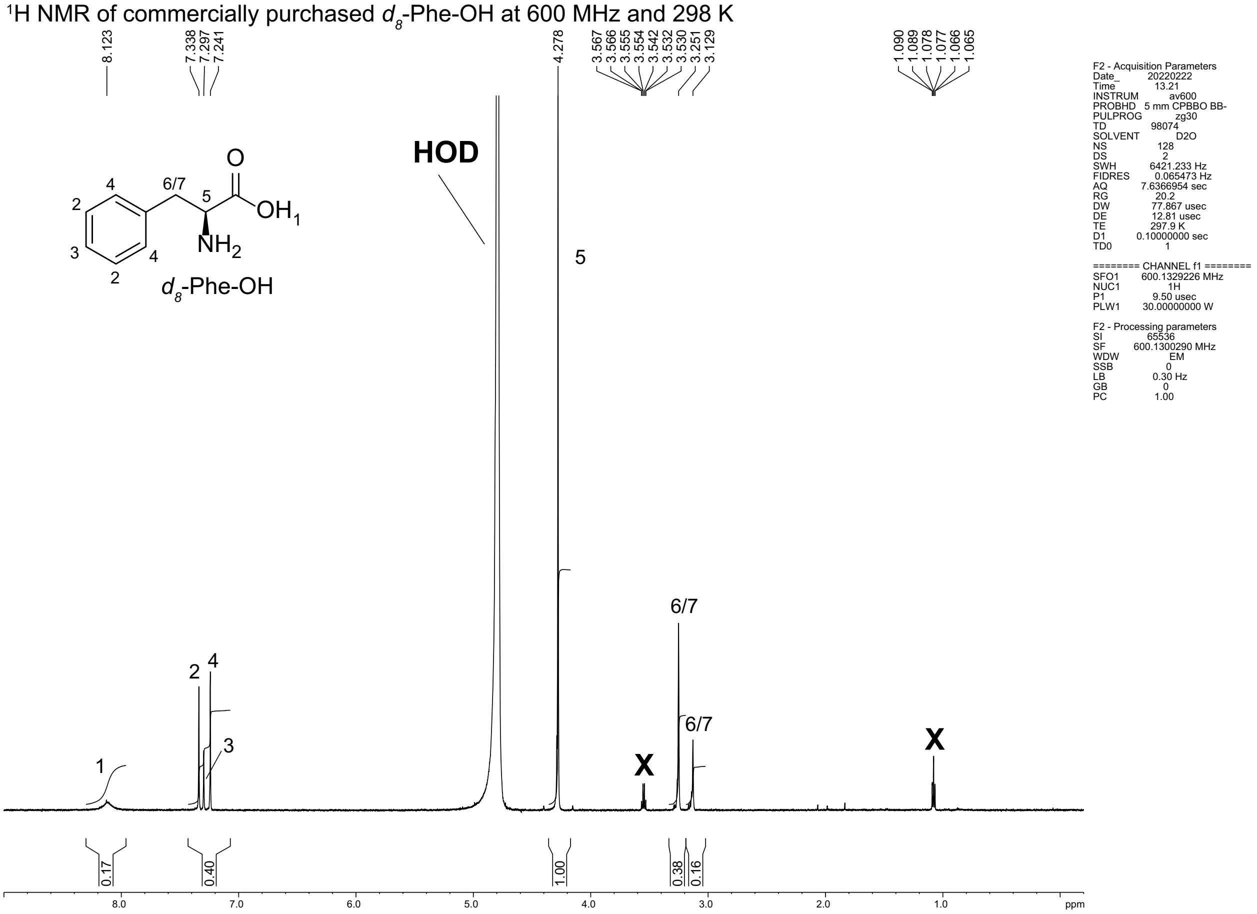

Figure S11. ^{1}H NMR spectrum of deuterated L-phenylalanine (d_8-Phe-OH) used to synthesize peptide **3a**. The d_8-Phe-OH was purchased from Cambridge Isotope Laboratories with a reported isotopic purity of 98%. The spectrum was recorded in D_2O with DCl at 600 MHz and 298 K. Incomplete deuterium labeling at the α-position is disproportionately large. Thus, the relative integration of the α-proton is 2.5X that of the aromatic protons and 2X that of the β-protons. If the ^{1}H isotopic impurity was uniformly distributed, the integration of the α-proton would be 0.2X that of the aromatic protons and 0.5X that of the β-protons.

Materials and Methods[3]

General Information

Chemicals and Supplies. All chemicals were used as received unless otherwise noted. Dry methylene chloride (CH_2Cl_2), was obtained by passing through alumina under argon prior to use. Anhydrous, amine-free *N,N*-dimethylformamide (DMF), 2,4,6-collidine, and piperidine were purchased from Alfa Aesar. HPLC grade acetonitrile (CH_3CN) was purchased from Fisher Scientific and deionized water (18 MΩ), each containing 0.1% trifluoroacetic acid (TFA), were used for analytical and preparative reverse-phase HPLC, as well as for reverse-phase chromatography using a Biotage Isolera One flash chromatography instrument. Commercial reagents were used without purification, unless otherwise stated. Boc-Orn(Fmoc)-OH, HCTU, HATU, and HOAT were purchased from GL Biochem Ltd (Shanghai). 2-Chlorotrityl chloride resin and Fmoc-protected amino acids were purchased from Chem-Impex International. *N,N*-Diisopropylethylamine (DIPEA), *N*-methylmorpholine (NMM), trifluoroacetic acid (TFA), and triisopropylsilane (TIPS) were purchased from Oakwood Chemical. Isotopically labeled glycine (^{15}N, 98% isotopic purity), phenylalanine (^{15}N, 98% isotopic purity), phenylalanine (d_8-Phe, 98% isotopic purity), alanine (d_4-Ala, 98% isotopic purity), and deuterium oxide (99.96% isotopic purity) were purchased from Cambridge Isotope Laboratories. *N*-(9-fluorenylmethoxycarbonyloxy)succinimide (Fmoc-OSu) was purchased from GL Biochem. Fmoc-Hao-OH was synthesized according to previously reported procedures.[4]

Instrumentation. NMR spectra were recorded on a Bruker AVANCE 500 spectrometer equipped with Bruker TCI helium-cooled cryoprobe and a Bruker AVANCE 600 spectrometer equipped with Bruker CBBFO helium-cooled cryoprobe. Spectra were calibrated using residual solvent peaks (HOD: $\delta_H = 4.79$ ppm or DSA[1] $\delta_H = 0.00$ ppm). MALDI-TOF mass spectra were

acquired using AB SCIEX TOF/TOF 5800 System. Analytical reverse-phase HPLC was performed on an Agilent 1260 Infinity II instrument equipped with a Phenomonex bioZen PEPTIDE 2.6 um XB-C18 column (150 x 4.6 mm), eluting with a gradient of acetonitrile and water (each containing 0.1% trifluoracetic acid) from 5–100% over 20 minutes. Peptides were first pre-purified on a Biotage Isolera One flash chromatography instrument with a Biotage® Sfär Bio C18 D - Duo 300 Å 20 µm 25 g column, and then purified by preparative reverse-phase HPLC on a Rainin Dynamax equipped with an Agilent Zorbax 250 x 21.2 mm SB-C18 column. All peptides were prepared and used as the trifluoroacetate salts and were assumed to have one trifluoracetic acid molecule per amine group on each peptide.

Synthesis of Peptides

Peptide synthesis procedure. Macrocyclic peptides **1a**, **1b,** and homologues were synthesized by manual solid-phase peptide synthesis of the corresponding linear peptide on 2-chlorotrityl resin, followed by solution-phase cyclization, deprotection, and purification. A step-by-step procedure is detailed below.

a. Loading the resin. 2-Chlorotrityl chloride resin (300 mg, 1.07 mmol/g) was added to a Bio-RAD Poly-Prep chromatography column (10 mL). Dry CH_2Cl_2 (8 mL) was used to suspend and swell the resin for 30 min with gentle rocking. After the solution was drained from the resin, a separate solution of N^{α}-Boc-N^{δ}-Fmoc-L-ornithine (Boc-Orn(Fmoc)-OH, 0.7 equiv, 100 mg) in 6% (v/v) 2,4,6-collidine in dry CH_2Cl_2 (8 mL) was added and the suspension was gently rocked for 6–14 h. The solution was then drained and a mixture of CH_2Cl_2/MeOH/*N*,*N*-diisopropylethylamine (DIPEA) (17:2:1, 8 mL) was added immediately. The resin was gently rocked for 1 h, to cap the unreacted 2-chlorotrityl chloride resin sites. The resin was then washed

three times with dry CH_2Cl_2 and dried by passing nitrogen through the vessel. This procedure typically yields 0.15–0.20 mmol of loaded resin, as assessed by spectrophotometric analysis.

b. Manual peptide coupling. The loaded resin was suspended in dry DMF and then transferred to a solid-phase peptide synthesis vessel. Each residue was manually coupled using Fmoc-protected amino acid building blocks. The coupling cycle consisted of *i*. Fmoc-deprotection with of 20% (v/v) piperidine in DMF (5 mL) for 5–10 min at room temperature, *ii*. washing with dry DMF (4 x 5 mL), *iii*. coupling of the amino acid (4 equiv) with HCTU (4 equiv) in 20% (v/v) 2,4,6-collidine in dry DMF (5 mL) for 20–30 min, and *iv*. washing with dry DMF (4 x 5 mL). [NOTE: The unnatural amino acid Fmoc-Hao-OH (2.0 equiv) was coupled twice with 2.0 equiv of HCTU per coupling, for 1 h each to achieve complete coupling.[4]] *v*. After the last amino acid was coupled, and its Fmoc protecting group deprotected, the resin was transferred from the solid-phase peptide synthesis vessel to a new BioRad Poly-Prep chromatography column. The resin was washed with CH_2Cl_2 (3 x 5 mL) and dried by passing nitrogen through the column.

c. Cleavage of the linear peptide from chlorotrityl resin. The linear peptide was cleaved from the resin by rocking the resin in a solution of 20% (v/v) 1,1,1,3,3,3-hexafluoroispropanol (HFIP) in CH_2Cl_2 (8 mL) for 1 h. The suspension was filtered, and the filtrate was collected in a 250-mL round-bottomed flask. The resin was washed with additional cleavage solution (8 mL) for 30 min and filtered into the same 250 mL round bottom-bottomed flask. The combined filtrates were concentrated by rotary evaporation and further dried by vacuum pump to afford the crude protected linear peptide, which was cyclized without further purification.

d. Cyclization of the linear peptide. The crude protected linear peptide was dissolved in dry DMF (125 mL). PyBOP (6 equiv) were dissolved in 8 mL of dry DMF in a test tube to which 300 μL of 4-methylmorpholine was added and the solution mixed until homogenous. The solution was

then added to the round-bottom flask containing the dissolved peptide and the mixture was stirred under nitrogen at room temperature for 48 h. The reaction mixture was concentrated by rotary evaporation and further dried by vacuum pump to afford the crude protected cyclized peptide, which was immediately subjected to global deprotection.

e. Global deprotection of the cyclic peptide. For the preparation of peptides **1a,** *ent*-**1a**, **2a,** and **3a**, the protected cyclic peptides were dissolved in TFA:triisopropylsilane (TIPS):H_2O (18:1:1, 10 mL) in a 1000-mL round-bottomed flask equipped with a stir bar. The solution was stirred for 1 h under nitrogen. For the preparation of peptides **1b,** *ent*-**1b**, and **2b** containing a methionine amino acid, the protected cyclic peptides were dissolved in TFA:triisopropylsilane (TIPS):H_2O (18:1:1, 10 mL) with 350 mgs of ammonium iodide (NH_4I) and ca. 5 mL of dimethyl sulfide (DMS) in a 1000-mL round-bottomed flask equipped with a stir bar. The solution was stirred for 1 h under nitrogen.

During the 1 h deprotection, two 50-mL conical tubes containing 40 mL of dry Et_2O each were chilled on ice. After the 1 h deprotection, the peptide solution was split between the two conical tubes of Et_2O. The tubes were then centrifuged at 600x*g* for 10 min, decanted, and washed with fresh Et_2O. This process of decanting and washing was repeated for two more times. The pelleted peptides were dried under nitrogen for 15–20 min. The deprotected cyclic peptide was then purified by reverse-phase HPLC (RP-HPLC).

f. Reverse-phase HPLC purification. The peptide was dissolved in 20% CH_3CN in H_2O (5 mL) and pre-purified on a Biotage Isolera One flash chromatography instrument equipped with a Biotage® Sfär Bio C18 D - Duo 300 Å 20 µm 25 g column. The solution of crude cyclic peptide was injected at 20% CH_3CN and eluted with a gradient of 20–50% CH_3CN. Fractions containing the desired peptide was concentrated by rotary evaporation, diluted in 20% CH_3CN, injected on a

Rainin Dynamax instrument, and eluted over a gradient of 20–50% CH_3CN over 90 min. The collected fractions were analyzed by analytical HPLC and MALDI-TOF, and the pure fractions were concentrated by rotary evaporation and lyophilized. This synthesis typically yielded 40 mgs of peptides **1a** and **1b** isolated as the TFA salt.

Peptides *ent*-**1a**, *ent*-**1b**, **2a**, **2b**, and **3a** were synthesized in the same way as peptides **1a**.and **1b**. Commercially available D-amino acids were used to synthesize peptides *ent*-**1a** and *ent*-**1b**. Fmoc-protected ^{15}N L-phenylalanine replaced Phe_{20} in the preparation of labeled peptide **2a**. Fmoc-protected ^{15}N glycine replaced Gly_{33} in the preparation of labeled peptide **2b**. Fmoc-protected deuterated L-phenylalanine, ^{15}N L-phenylalanine, and deuterated L-alanine replaced Phe_{19}, Phe_{20}, and Ala_{21} in the preparation of labeled peptide **3a**. The syntheses typically yielded 20–40 mgs of each peptide as the TFA salt.

***Fmoc-Protection of ^{15}N-Labeled and Deuterated Amino Acid*[3]**

Fmoc-protected ^{15}N-labeled phenylalanine and Fmoc-protected ^{15}N-labeled glycine were prepared as previously reported.[3] Fmoc-protected deuterated L-phenylalanine and deuterated L-alanine were prepared in a similar fashion, as described below:

Fmoc-d_4-Ala-OH: A 100-mL one-neck round-bottom flask equipped with a magnetic stirring bar was charged with deuterated alanine (0.40 g, 4.3 mmol) and a solution of 1:1 CH_3CN/H_2O (25 mL). Et_3N (610 µL, 4.3 mmol) and Fmoc-OSu (1.32 g, 3.9 mmol) were added, then the reaction mixture was stirred for 10 minutes, and additional Et_3N was added until a clear solution formed (pH ca. 8.9). The mixture was stirred for 1 h. After the 1 h stir, the mixture was poured into a solution of 1.0 M HCl (200 mL) in a 400-mL beaker while stirring vigorously. The Fmoc-d_4-Ala-OH precipitated from the solution and the solid was isolated by filtering the mixture

through a sintered glass filter funnel with a medium frit. The funnel was covered with a piece of filter paper and the solid was dried by aspirating air through the funnel. The solid was suspended in ca. 100 mL of EtOAc to form a turbid solution. The solution was stirred vigorously for 10 min, dried over $MgSO_4$, filtered, and then concentrated under vacuum to give a white solid. The isolated solid was ground into a fine powder to give ca. 1.13 g (83%) of Fmoc-d_4-Ala-OH. A sample was re-dissolved in acetone, concentrated by rotary evaporation, dried under vacuum overnight, and evaluated by NMR spectroscopy. ^{1}H NMR (600 MHz, $CDCl_3$): δ 7.76 (d, J = 7.4 Hz, 2H), 7.59 (t, J = 6.3 Hz, 1.56H), 7.55 (bs, 0.44H), 7.40 (t, J = 7.4 Hz, 2H), 7.31 (t, J = 7.5 Hz, 2H), 6.25–5.46 (bs, 1H), 4.51 (m, 0.51H), 4.42 (m, 1.52H), 4.23 (appar t, J = 6.8 Hz, 1H). ^{13}C NMR (125 MHz, $CDCl_3$) δ 177.4, 156.0, 143.9, 143.8, 141.5, 127.9, 127.2, 125.2, 125.2, 124.9 (minor rotamer), 120.1, 67.7 (minor rotamer), 67.3, 49.1, 47.2, 17.7.

Fmoc-d_8-Phe-OH: A 100-mL one-neck round-bottom flask equipped with a magnetic stirring bar was charged with deuterated phenylalanine (0.49 g, 2.8 mmol) and a solution of 1:1 CH_3CN/H_2O (25 mL). Et_3N (390 µL, 2.8 mmol) and Fmoc-OSu (0.83 g, 2.6 mmol) were added, then the reaction mixture was stirred for 10 minutes, and additional Et_3N was added until a clear solution formed (pH ca. 8.7). The mixture was stirred for 1 h. After the 1 h stir, the mixture was poured into a solution of 1.0 M HCl (200 mL) in a 400-mL beaker while stirring vigorously. The Fmoc-d_8-Phe-OH precipitated from the solution and the solid was isolated by filtering the mixture through a sintered glass filter funnel with a medium frit. The funnel was covered with a piece of filter paper and the solid was dried by aspirating air through the funnel. The solid was suspended in ca. 100 mL of EtOAc to form a turbid solution. The solution was stirred vigorously for 10 min, dried over $MgSO_4$, filtered, and then concentrated under vacuum to give a white solid. The isolated solid was ground into a fine powder to give ca. 0.86 g (77%) of Fmoc-d_8-Phe-OH. A sample was

re-dissolved in acetone, concentrated by rotary evaporation, dried under vacuum overnight, and evaluated by NMR spectroscopy. ^{1}H NMR (600 MHz, DMSO-d_6): δ 12.71 (bs, 1H), 7.88 (d, J = 7.5 Hz, 2H), 7.73 (s, 1H), 7.64 (dd, J = 12.1 Hz, J = 7.4 Hz, 1.78H), 7.50 (dd, J = 13.1 Hz, J = 7.6 Hz, 0.28H), 7.41 (td, J = 7.4 Hz, J = 4.3 Hz, 2H), 7.30 (dt, J = 17.9 Hz, J = 7.5 Hz, 2H), 4.18 (m, 3H). ^{13}C NMR (125 MHz, DMSO-d_6) δ 173.4, 155.9, 143.8, 140.7, 140.7, 137.7, 128.7, 127.8, 127.6, 127.0, 125.9, 125.3, 125.2, 120.1, 65.6, 55.1, 46.6, 35.6.

NMR Spectroscopy of Unlabeled Peptides

Sample Preparation. NMR spectroscopic studies of peptides **1a**, *ent*-**1a**, **1b**, and *ent*-**1b** were performed in D_2O. The solutions were prepared by dissolving a weighed portion of the peptide in the appropriate volume of solvent. The molecular weights of the peptides were calculated as the TFA salts with all amino groups assumed to be protonated (**1a**, M.W. 2224.19; **1b**, M.W. 2100.20). 4,4-Dimethyl-4-silapentane-1-ammonium trifluoroacetate (DSA) was added as an internal standard for referencing chemical shifts.[1] The solutions were allowed to stand for at least 24–60 h to allow complete hydrogen to deuterium exchange of the amide NH protons.

TOCSY, NOESY, and ROESY Data Collection. NMR spectra were recorded on a Bruker 600 MHz spectrometer with a Bruker CBBFO helium-cooled cryoprobe. Presaturation water suppression was applied as needed. TOCSY spectra were recorded with 2048 points in the *f*2 dimension and either 512 or 256 increments in the *f*1 dimension with NS = 8 and a 150-ms spin-lock mixing time. NOESY spectra were recorded with 2048 points in the *f*2 dimension and 512 increments in the *f*1 dimension with NS = 16 and a 200-ms mixing time. EXSY (ROESY) spectra were recorded with 2048 points in the *f*2 dimension and 256 increments in the *f*1 dimension with NS = 16 and a 500-ms mixing time.

TOCSY, NOESY, and ROESY Data Processing. NMR spectra were processed with Bruker TopSpin software. Automatic baseline correction was applied in both dimensions after phasing the spectra. 2D TOCSY, NOESY, and ROESY spectra were Fourier transformed to a final matrix size of 1024 x 1024 real points using a Qsine weighting function and forward linear prediction in the $f1$ dimension.

Diffusion-Ordered Spectroscopy (DOSY) Experiments. DOSY experiments were performed on a Bruker 600 MHz spectrometer equipped with a Bruker CBBFO helium-cooled cryoprobe, with a diffusion delay (Δ) of 75-ms and a diffusion gradient length (δ) of 2.5-ms. Sixteen sets of FIDs were recorded with the gradient strength incremented from 5%–95% using a linear ramp. The combined FIDs were Fourier transformed in Bruker's TopSpin software to give a pseudo-2D spectrum. After phasing and performing baseline correction, each pseudo-2D spectrum was processed with logarithmic scaling on the Y-axis. The Y-axis was calibrated to the diffusion coefficient of the residual HOD peak in D_2O (1.9×10^{-9} m^2/s at 298 K).[2] The diffusion coefficients of the peptides were read and converted from logarithmic values to linear values.

NMR Spectroscopy of ^{15}N-Labeled Peptides

Sample Preparation. NMR spectroscopic studies of peptides **2a**, **3a**, and **2b** were performed in 9:1 H_2O/D_2O. The solutions were prepared by dissolving a weighed portion of the peptide in the appropriate volume of solvent. The molecular weights of the peptides were calculated as the TFA salts with all amino groups assumed to be protonated (**2a**, M.W. 2225.18; **3a**, M.W. 2236.25; **2b** M.W. 2101.19). 4,4-Dimethyl-4-silapentane-1-ammonium trifluoroacetate (DSA) was added as an internal standard for referencing chemical shifts.[1]

$^1H,^{15}N$ HSQC, and $^1H,^{15}N$ NOESY-HSQC (^{15}N-edited NOESY) Data Collection. NMR spectra were recorded on a Bruker AVANCE 500 MHz spectrometer with Bruker TCI helium-cooled cryoprobe. Gradient water suppression was applied as needed. $^1H,^{15}N$ HSQC spectra were generally recorded with 2048 points in the $f2$ dimension and 512 increments in the $f1$ dimension with NS = 16, but the spectrum of 8.0 mM peptide **1a** was taken with 1024 in the $f1$ and with NS = 8. $^1H,^{15}N$ NOESY-HSQC spectra were recorded as follows: Peptide **2a**, 250-ms mixing time, 2048 points in the $f3$ dimension (^1H), 1 increment in the $f2$ dimension (^{15}N), and 512 increments in the $f1$ dimension (^1H). Mixture of peptides **2a** and *ent*-**1a**, 250-ms mixing time, 4096 points in the $f3$ dimension (^1H), 1 increment in the $f2$ dimension (^{15}N), and 1024 increments in the $f1$ dimension (^1H). Peptide **3a**, 250-ms mixing time, 2048 points in the $f3$ dimension (^1H), 1 increment in the $f2$ dimension (^{15}N), and 512 increments in the $f1$ dimension (^1H). Mixture of peptides **3a** and *ent*-**1a**, 250-ms mixing time, 2048 points in the $f3$ dimension (^1H), 1 increment in the $f2$ dimension (^{15}N), and 512 increments in the $f1$ dimension (^1H).

$^1H,^{15}N$ HSQC, and $^1H,^{15}N$ NOESY-HSQC (^{15}N-edited NOESY) Data Processing. NMR spectra were Fourier transformed in Bruker TopSpin software. Automatic baseline correction was applied in both dimensions after phasing the spectra. The $^1H,^{15}N$ HSQC spectra were processed to a final matrix size of 2048 x 512 real points with GB = 0.1 in both f dimensions using a Qsine weighting function, and forward linear prediction in the $f1$ dimension. The $^1H,^{15}N$ NOESY-HSQC spectra were processed to a final matrix size of 4096 x 2048 real points ($f3$, $f1$) with GB = 0.05 in both dimensions, using a Qsine weighting function and forward linear prediction in the $f1$ dimension.

References

1. Nowick, J. S.; Khakshoor, O.; Hashemzadeh, M.; Brower, J. O. *Org. Lett*. **2003**, 5, 3511–3513.

2. Longsworth, L. G. *J. Phys. Chem*. **1960**, *64*, 1914–1917.

3. The general information and instrumentations follow closely to those that our laboratory has previously published. The procedures in this section are adapted from and in some cases taken verbatim from: Truex, N. L.; Wang, Y.; Nowick, J. S. *J. Am. Chem. Soc.* **2016**, *138*, 13882–13890.

4. (a) Nowick, J. S.; Chung, D. M.; Maitra, K.; Maitra, S.; Stigers, K. D.; Sun, Y. *J. Am. Chem. Soc.* **2000**, *122*, 7654–7661. (b) Cheng, P.–N.; Nowick, J. S. *J. Org. Chem*. **2011**, *76*, 3166–3173.

^{1}H NMR of Fmoc-protected d_4-Ala-OH at 600 MHz and 298 K

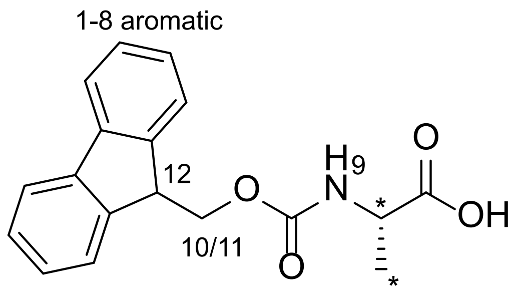

Fmoc-d_4-Ala-OH * = CD group

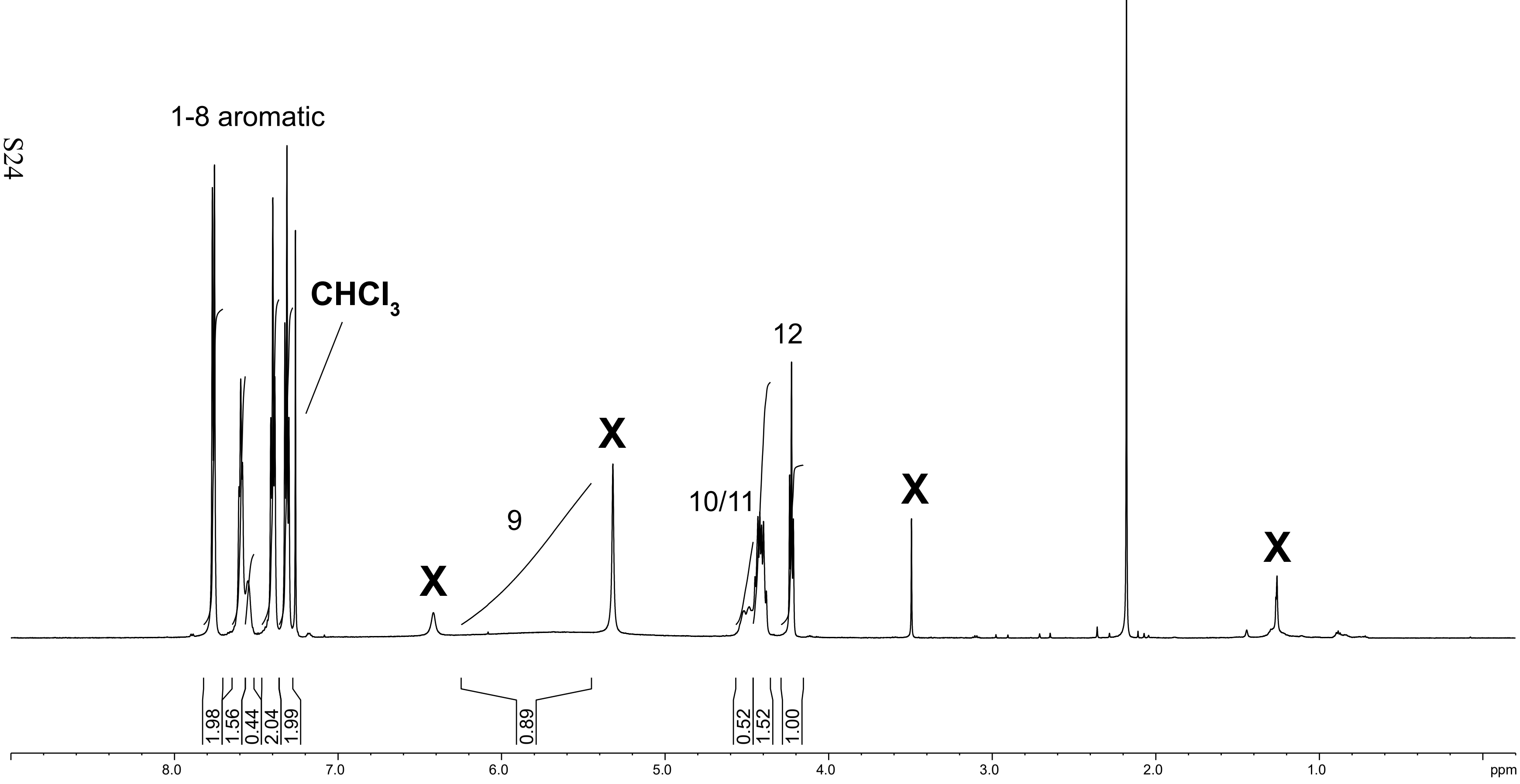

F2 - Acquisition Parameters
Date_ 20220311
Time 14.13
INSTRUM av600
PROBHD 5 mm CPBBO BB-
PULPROG zg30
TD 98074
SOLVENT CDCl3
NS 8
DS 2
SWH 9615.385 Hz
FIDRES 0.098042 Hz
AQ 5.0998478 sec
RG 10
DW 52.000 usec
DE 14.23 usec
TE 298.0 K
D1 0.10000000 sec
TD0 1

======== CHANNEL f1 ========
SFO1 600.1342009 MHz
NUC1 1H
P1 9.50 usec
PLW1 30.00000000 W

F2 - Processing parameters
SI 65536
SF 600.1300352 MHz
WDW EM
SSB 0
LB 1.00 Hz
GB 0
PC 1.00

^{13}C NMR of Fmoc-protected d_4-Ala-OH at 600 MHz and 298 K

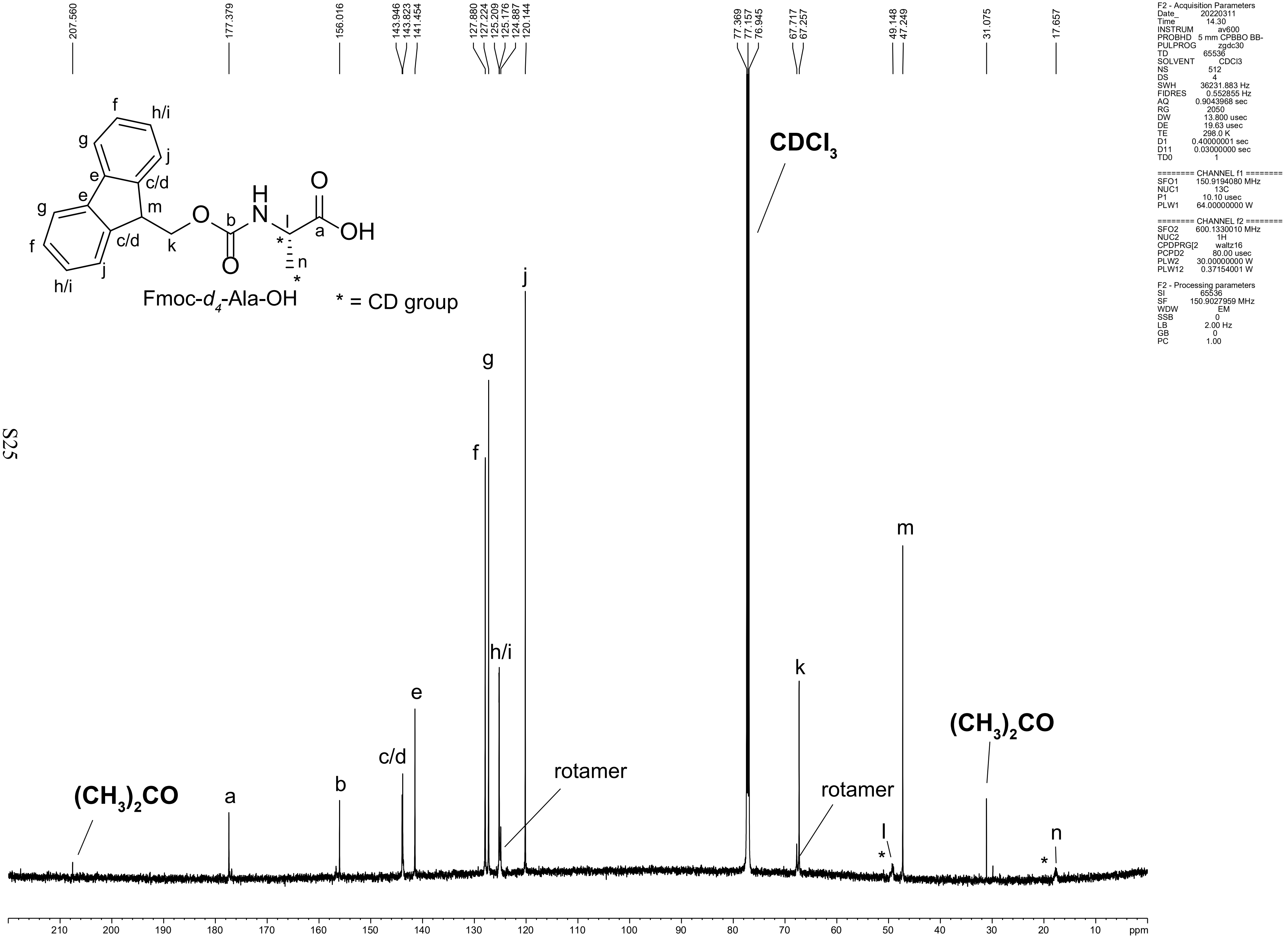

^1H NMR of Fmoc-protected d_8-Phe-OH at 600 MHz and 298 K

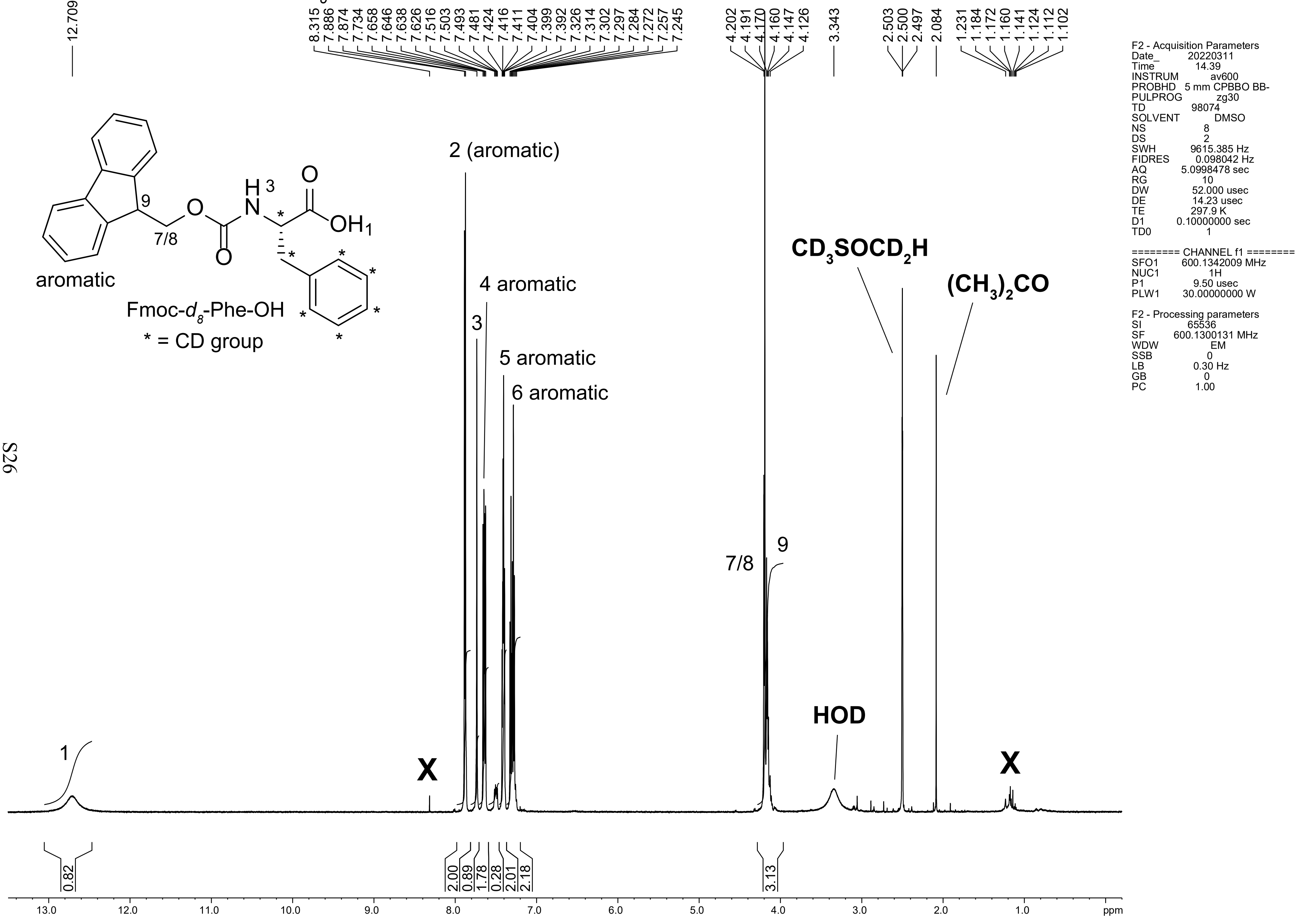

^{13}C NMR of Fmoc-protected d_8-Phe-OH at 600 MHz and 298 K

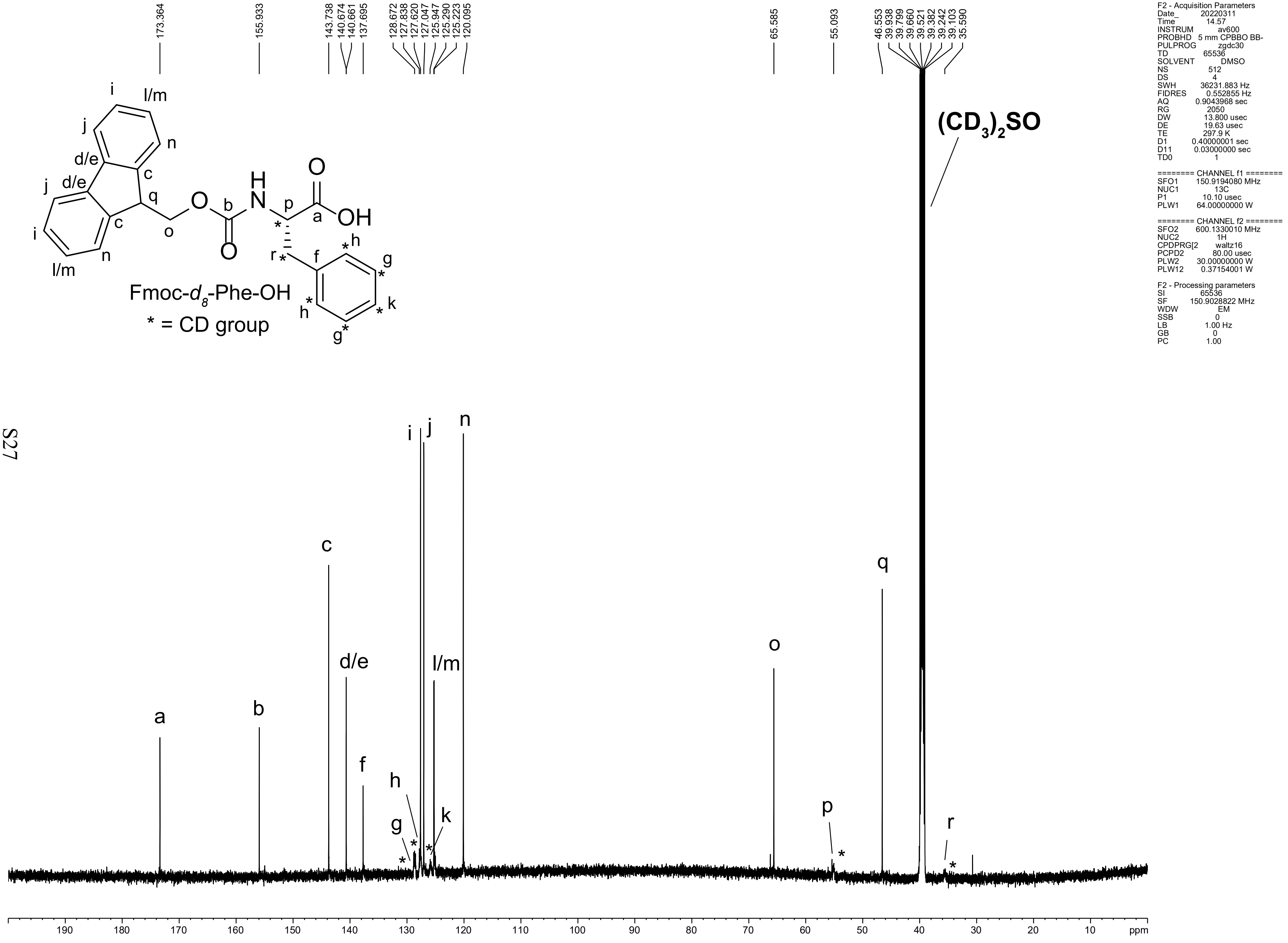

^{1}H NMR of 8.0 mM peptide **1a** in D_2O at 600 MHz and 298 K

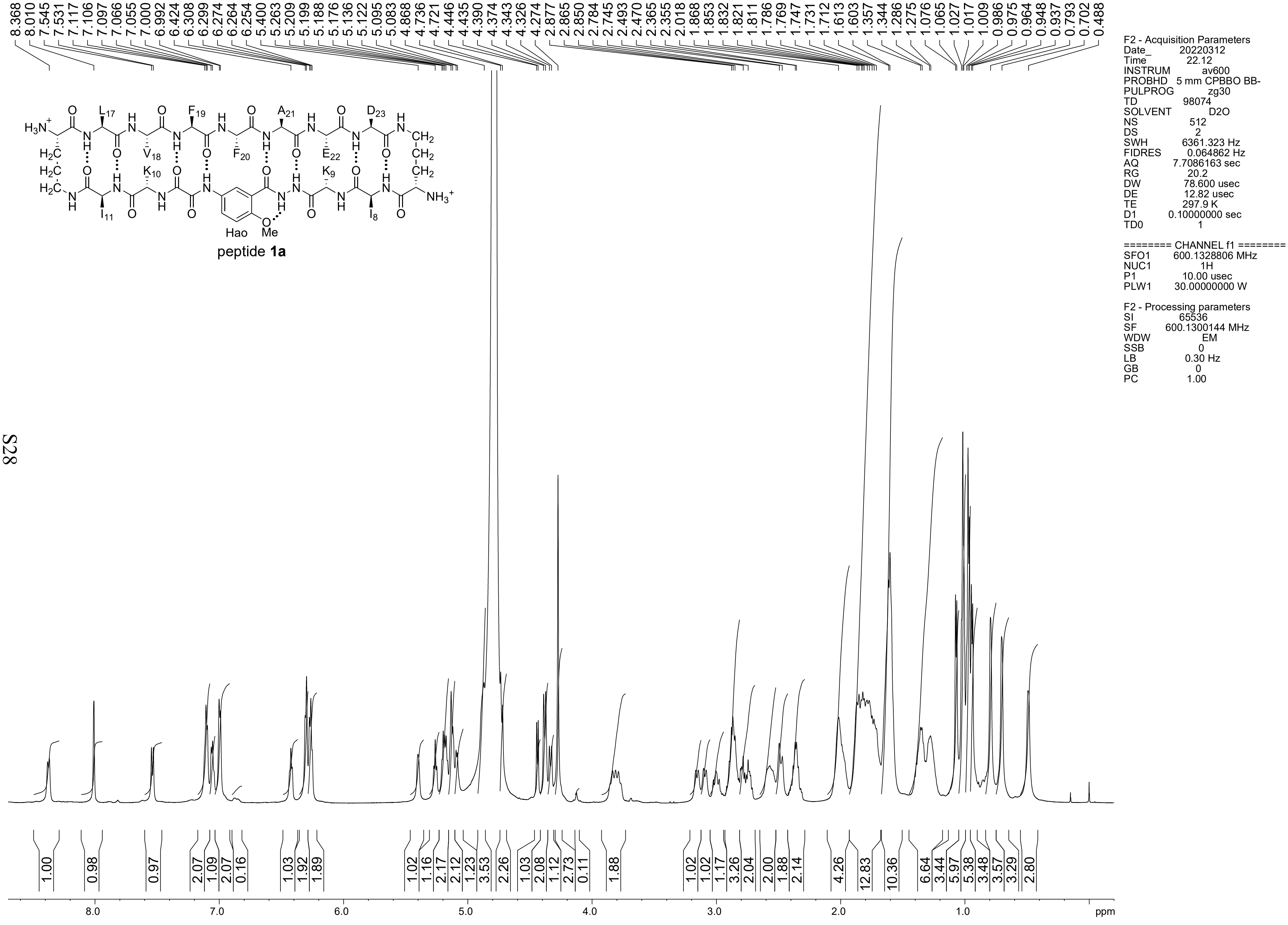

peptide **1a**

^{1}H NMR of 8.0 mM peptide **1a** in D_2O at 600 MHz and 298 K

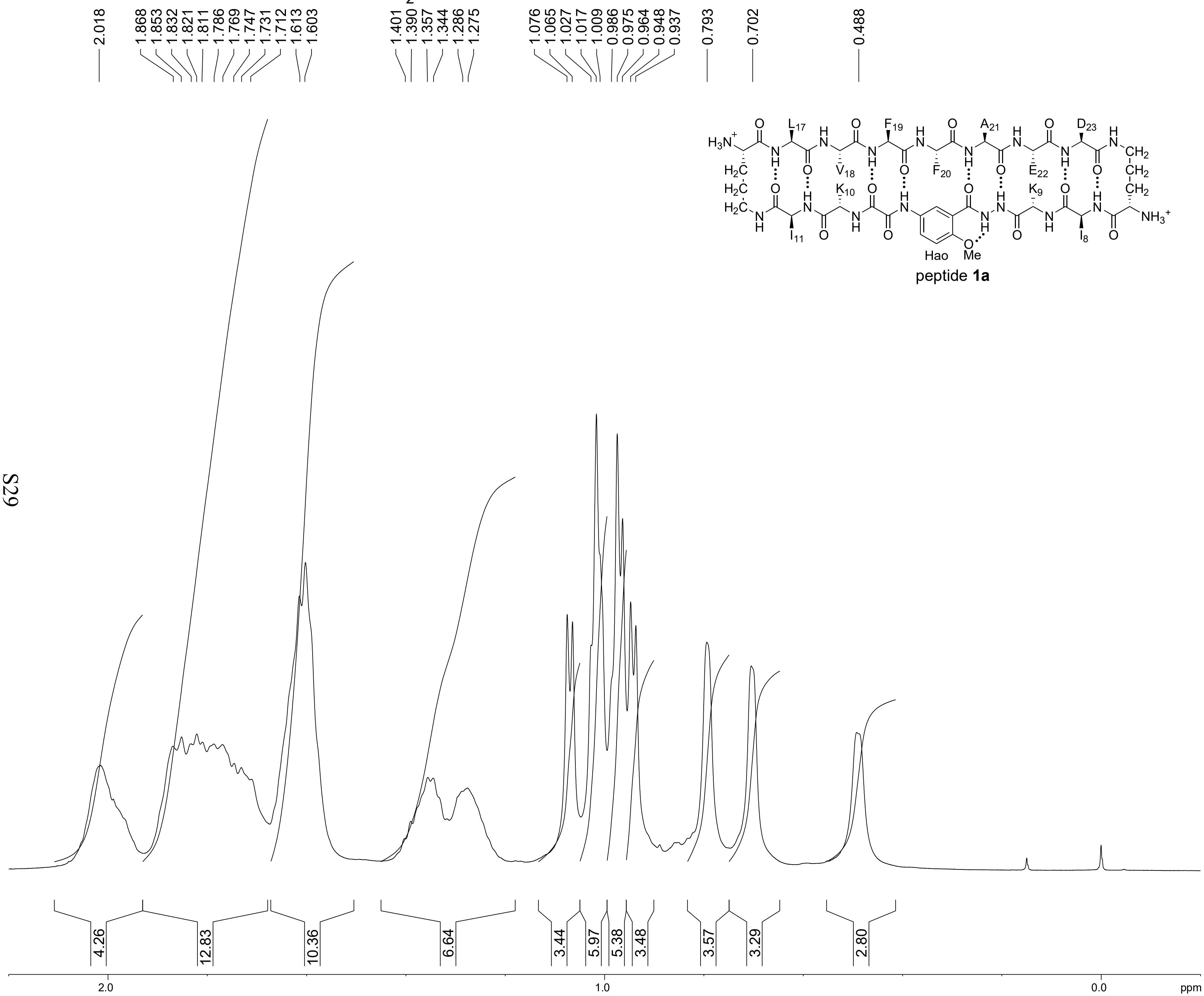

F2 - Acquisition Parameters
Date_ 20220312
Time 22.12
INSTRUM av600
PROBHD 5 mm CPBBO BB-
PULPROG zg30
TD 98074
SOLVENT D2O
NS 512
DS 2
SWH 6361.323 Hz
FIDRES 0.064862 Hz
AQ 7.7086163 sec
RG 20.2
DW 78.600 usec
DE 12.82 usec
TE 297.9 K
D1 0.10000000 sec
TD0 1

======== CHANNEL f1 ========
SFO1 600.1328806 MHz
NUC1 1H
P1 10.00 usec
PLW1 30.00000000 W

F2 - Processing parameters
SI 65536
SF 600.1300144 MHz
WDW EM
SSB 0
LB 0.30 Hz
GB 0
PC 1.00

^{1}H NMR of 8.0 mM peptide **1a** in D_2O at 600 MHz and 298 K

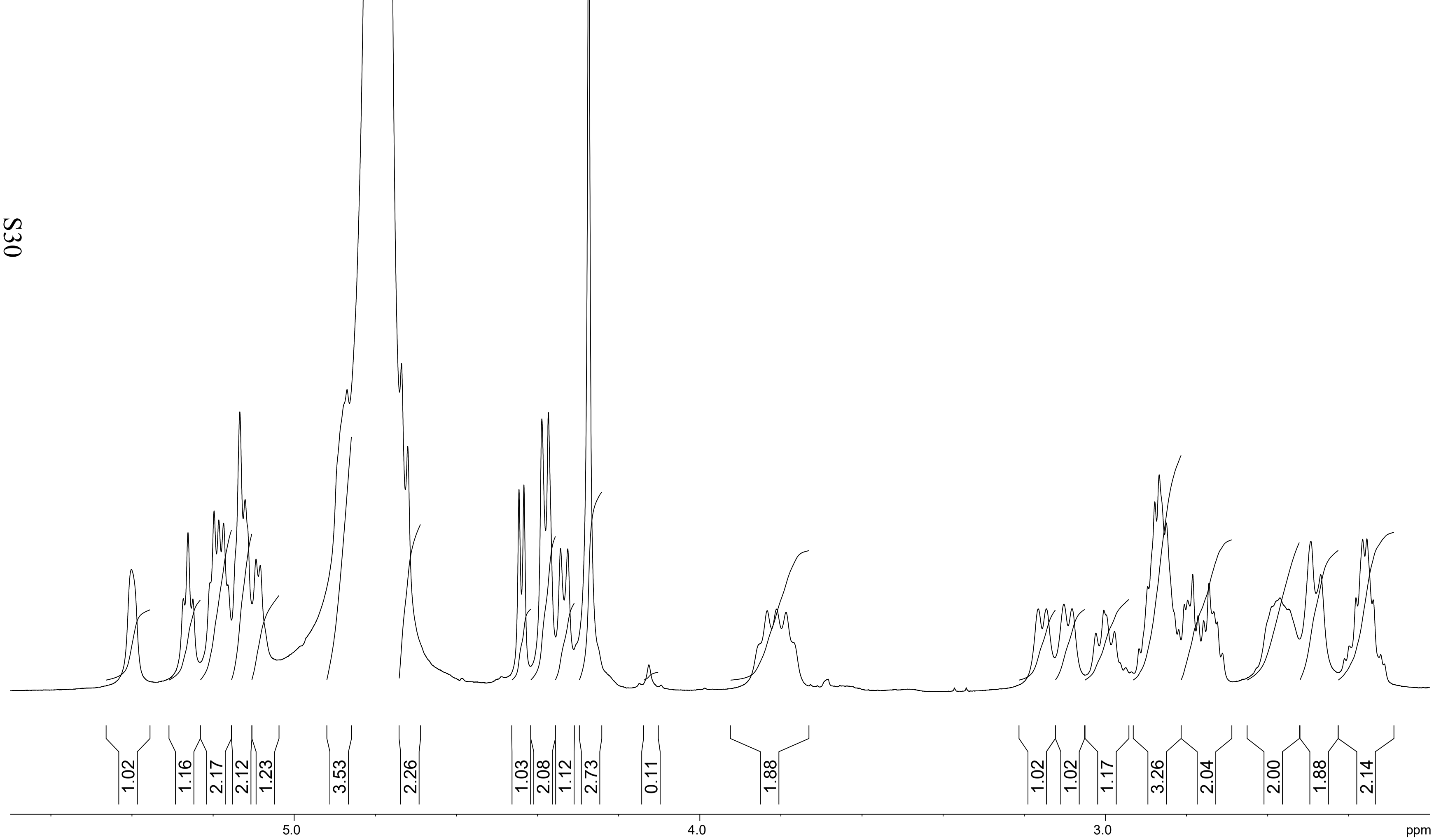

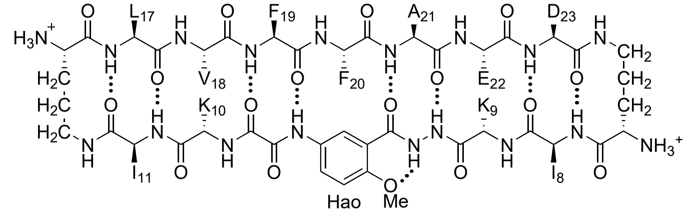

peptide **1a**

F2 - Acquisition Parameters
Date_ 20220312
Time 22.12
INSTRUM av600
PROBHD 5 mm CPBBO BB-
PULPROG zg30
TD 98074
SOLVENT D2O
NS 512
DS 2
SWH 6361.323 Hz
FIDRES 0.064862 Hz
AQ 7.7086163 sec
RG 20.2
DW 78.600 usec
DE 12.82 usec
TE 297.9 K
D1 0.10000000 sec
TD0 1

======== CHANNEL f1 ========
SFO1 600.1328806 MHz
NUC1 1H
P1 10.00 usec
PLW1 30.00000000 W

F2 - Processing parameters
SI 65536
SF 600.1300144 MHz
WDW EM
SSB 0
LB 0.30 Hz
GB 0
PC 1.00

^1H NMR of 8.0 mM peptide **1a** in D_2O at 600 MHz and 298 K

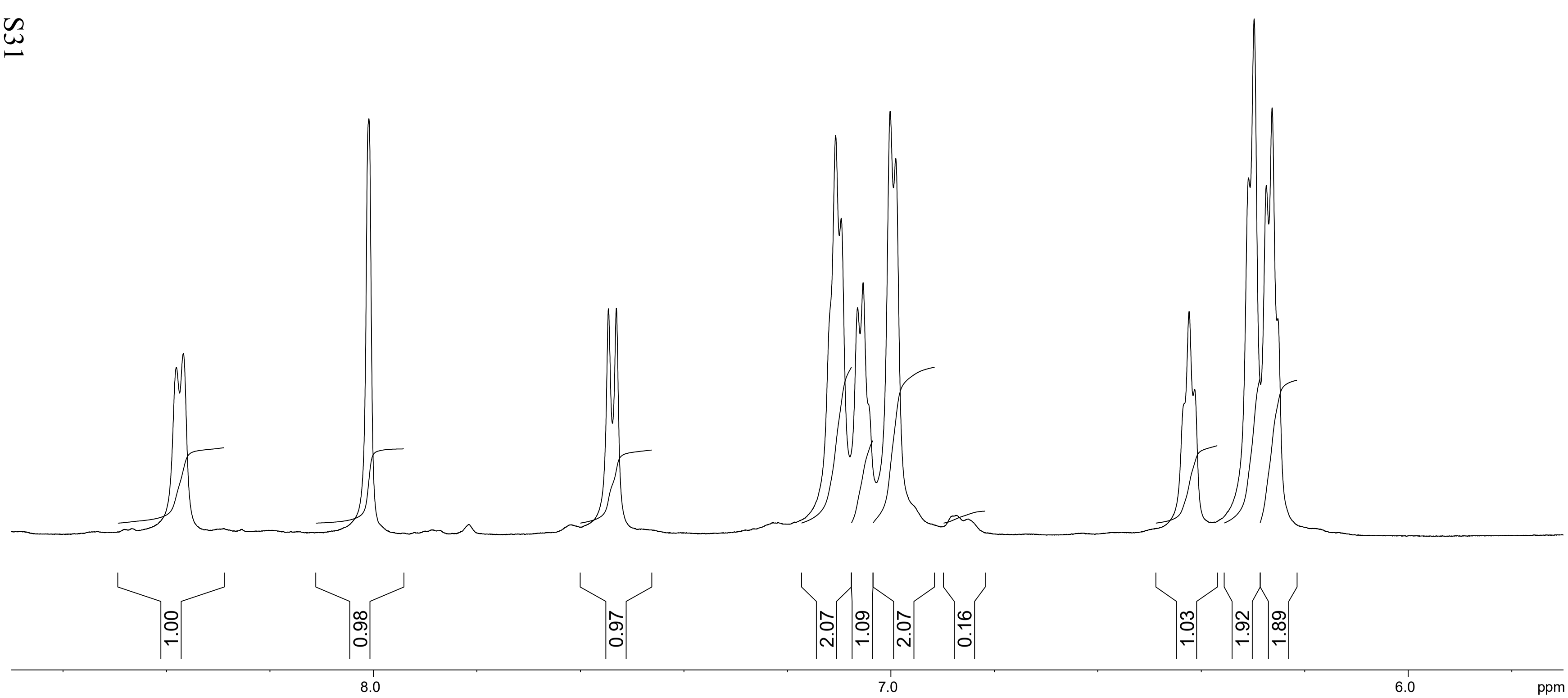

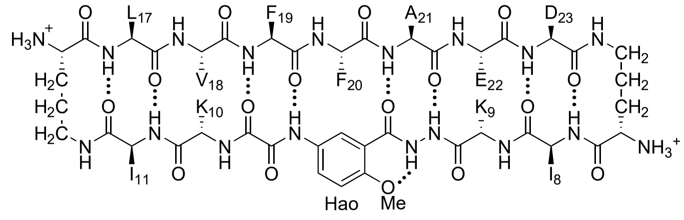

peptide **1a**

F2 - Acquisition Parameters
Date_ 20220312
Time 22.12
INSTRUM av600
PROBHD 5 mm CPBBO BB-
PULPROG zg30
TD 98074
SOLVENT D2O
NS 512
DS 2
SWH 6361.323 Hz
FIDRES 0.064862 Hz
AQ 7.7086163 sec
RG 20.2
DW 78.600 usec
DE 12.82 usec
TE 297.9 K
D1 0.10000000 sec
TD0 1

======== CHANNEL f1 ========
SFO1 600.1328806 MHz
NUC1 1H
P1 10.00 usec
PLW1 30.00000000 W

F2 - Processing parameters
SI 65536
SF 600.1300144 MHz
WDW EM
SSB 0
LB 0.30 Hz
GB 0
PC 1.00

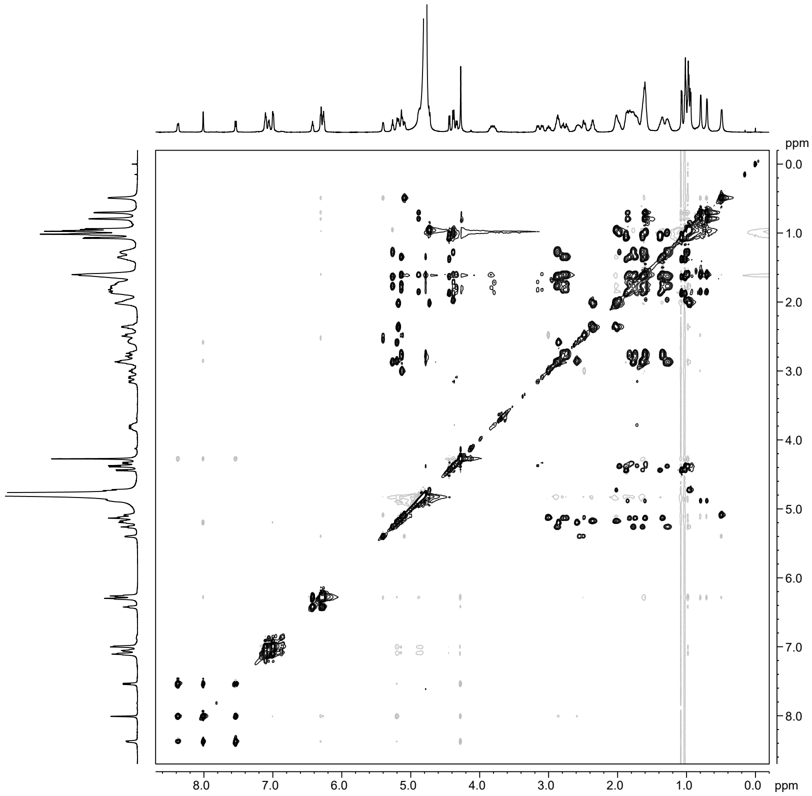

8.0 mM peptide **1a**
in D_2O, 150-ms mixing time, 298 K
600 MHz TOCSY spectrum
presaturation suppression of the HOD peak

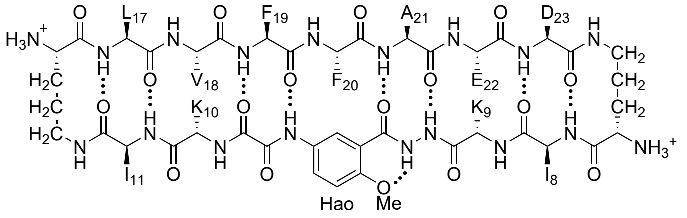

peptide **1a**

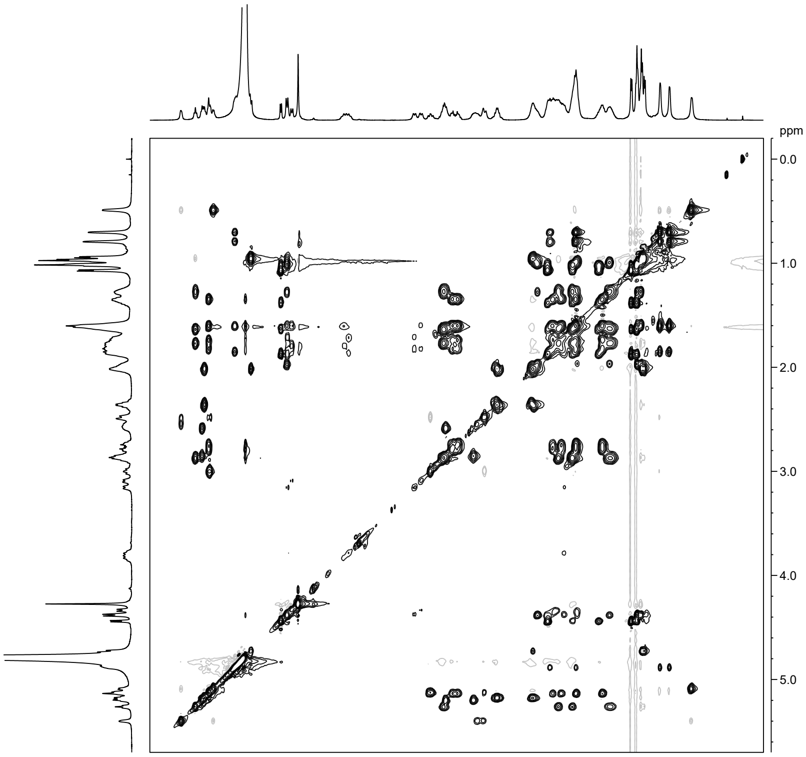

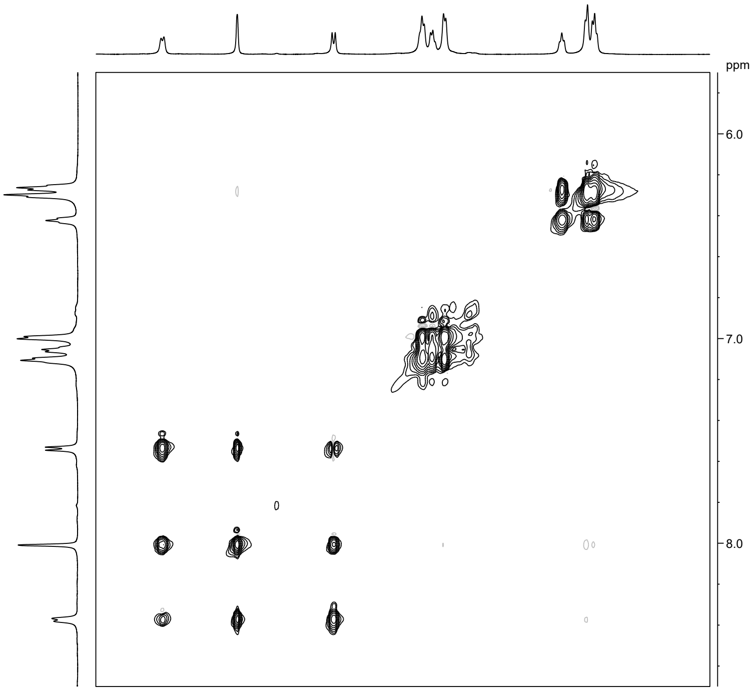

8.0 mM peptide **1a**
in D_2O, 200-ms mixing time, 298 K
600 MHz NOESY spectrum
presaturation suppression of the HOD peak

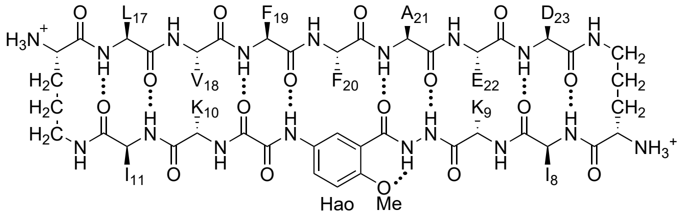

peptide **1a**

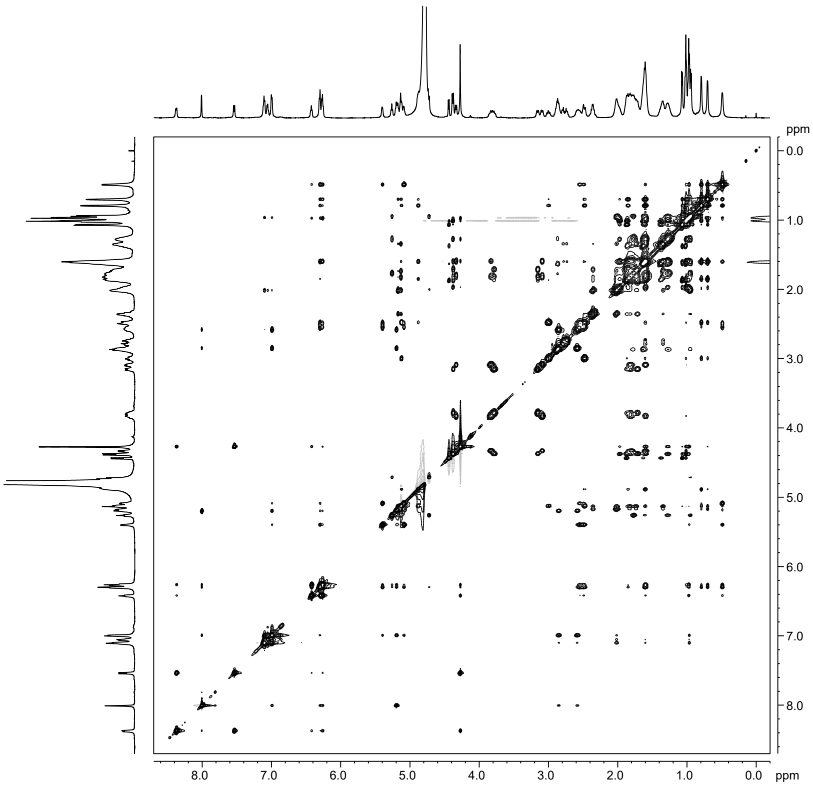

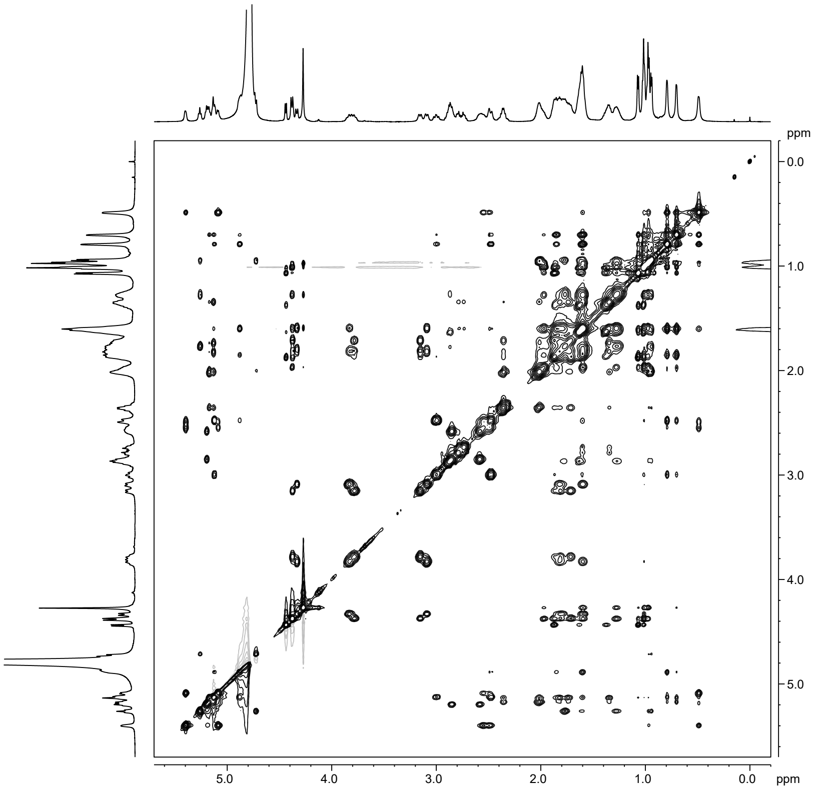

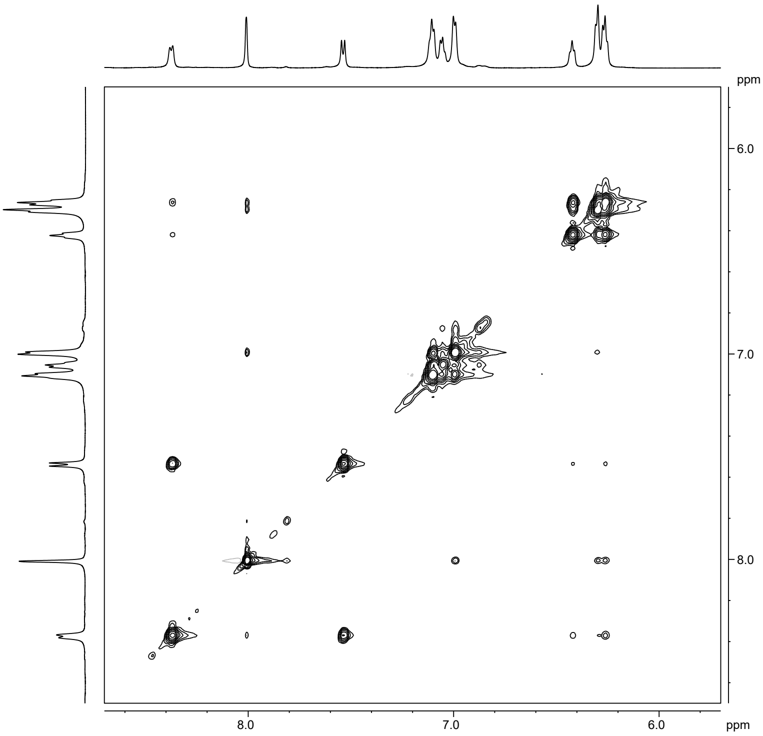

^1H NMR of 8.0 mM peptide **1a** + 8.0 mM peptide *ent*-**1a** in D_2O at 600 MHz and 298 K

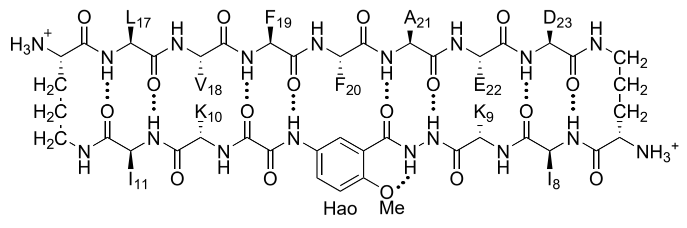

peptide **1a**

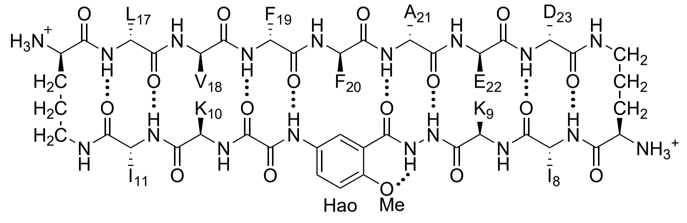

ent-**1a**

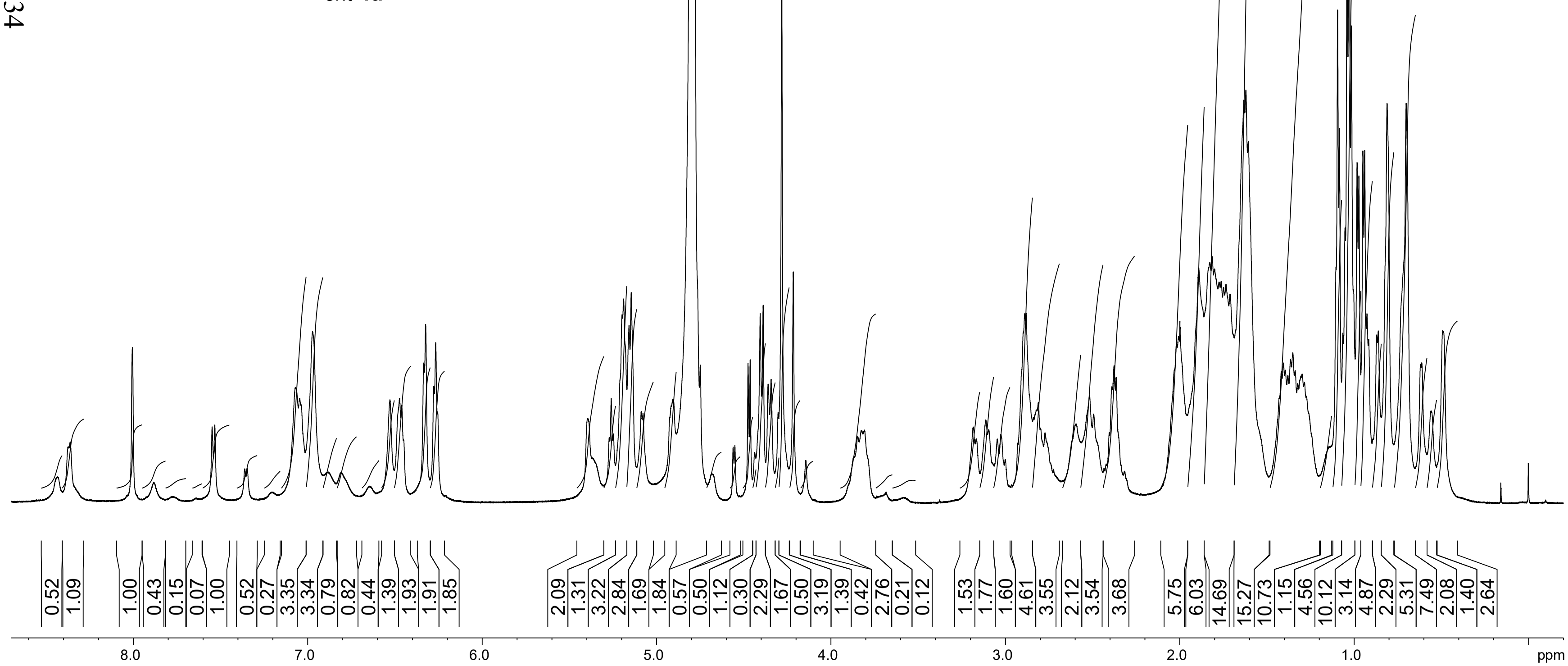

F2 - Acquisition Parameters
Date_ 20211014
Time 23.27
INSTRUM av600
PROBHD 5 mm CPBBO BB-
PULPROG zg30
TD 98074
SOLVENT D2O
NS 512
DS 2
SWH 6602.113 Hz
FIDRES 0.067318 Hz
AQ 7.4274712 sec
RG 10
DW 75.733 usec
DE 12.12 usec
TE 298.0 K
D1 0.10000000 sec
TD0 1

======== CHANNEL f1 ========
SFO1 600.1328835 MHz
NUC1 1H
P1 10.88 usec
PLW1 30.00000000 W

F2 - Processing parameters
SI 65536
SF 600.1300079 MHz
WDW no
SSB 0
LB 0 Hz
GB 0
PC 1.00

^{1}H NMR of 8.0 mM peptide **1a** + 8.0 mM peptide *ent*-**1a** in D_2O at 600 MHz and 298 K

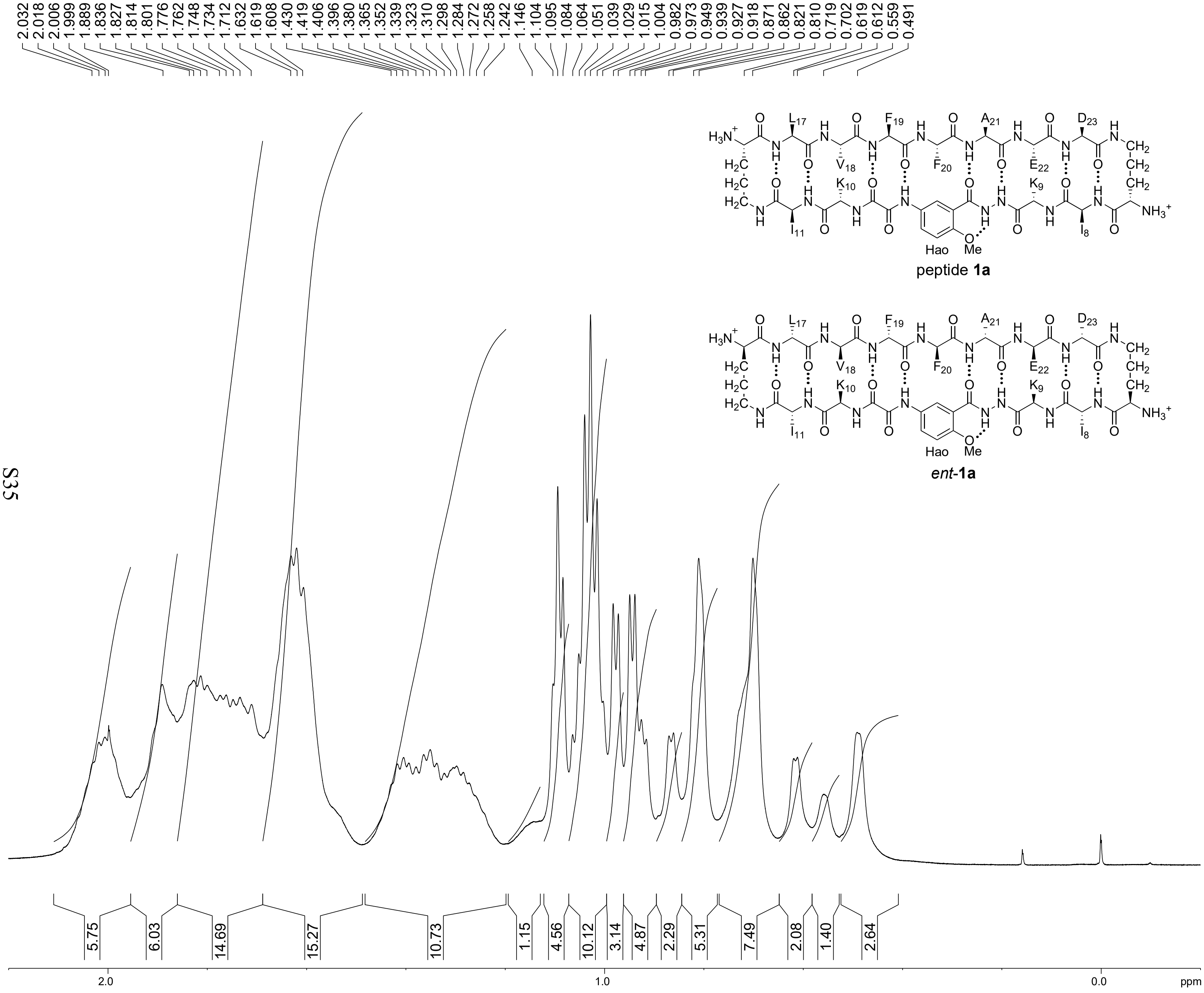

F2 - Acquisition Parameters
Date_ 20211014
Time 23.27
INSTRUM av600
PROBHD 5 mm CPBBO BB-
PULPROG zg30
TD 98074
SOLVENT D2O
NS 512
DS 2
SWH 6602.113 Hz
FIDRES 0.067318 Hz
AQ 7.4274712 sec
RG 10
DW 75.733 usec
DE 12.12 usec
TE 298.0 K
D1 0.10000000 sec
TD0 1

======== CHANNEL f1 ========
SFO1 600.1328835 MHz
NUC1 1H
P1 10.88 usec
PLW1 30.00000000 W

F2 - Processing parameters
SI 65536
SF 600.1300079 MHz
WDW no
SSB 0
LB 0 Hz
GB 0
PC 1.00

^{1}H NMR of 8.0 mM peptide **1a** + 8.0 mM peptide *ent*-**1a** in D_2O at 600 MHz and 298 K

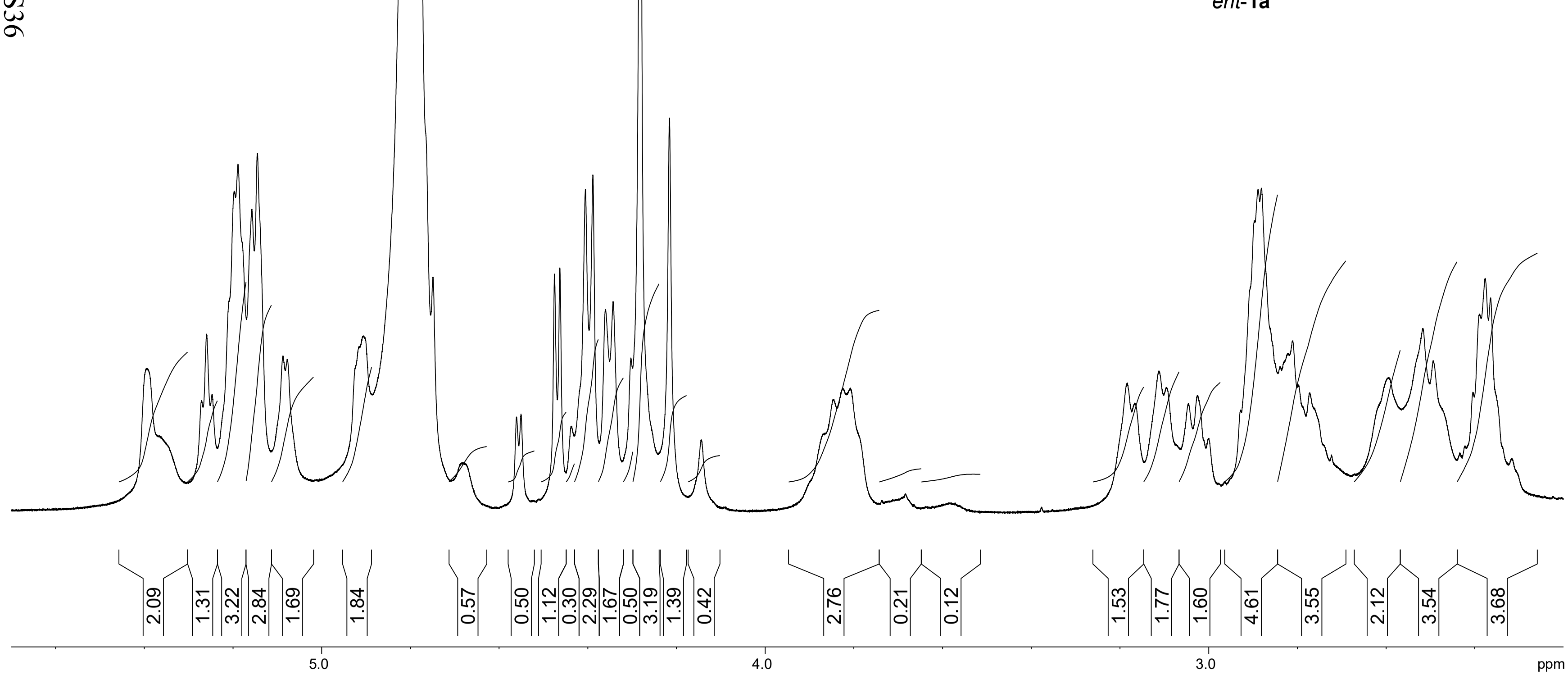

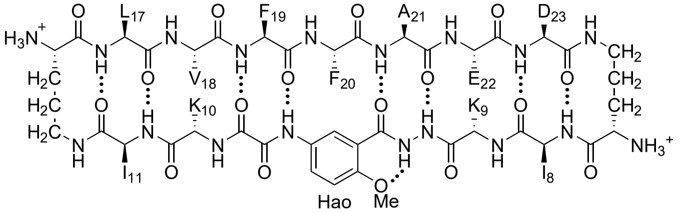

peptide **1a**

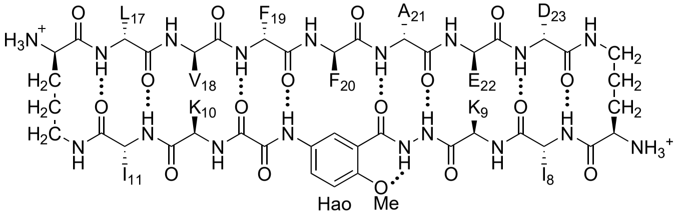

ent-**1a**

F2 - Acquisition Parameters
Date_ 20211014
Time 23.27
INSTRUM av600
PROBHD 5 mm CPBBO BB-
PULPROG zg30
TD 98074
SOLVENT D2O
NS 512
DS 2
SWH 6602.113 Hz
FIDRES 0.067318 Hz
AQ 7.4274712 sec
RG 10
DW 75.733 usec
DE 12.12 usec
TE 298.0 K
D1 0.10000000 sec
TD0 1

======== CHANNEL f1 ========
SFO1 600.1328835 MHz
NUC1 1H
P1 10.88 usec
PLW1 30.00000000 W

F2 - Processing parameters
SI 65536
SF 600.1300079 MHz
WDW no
SSB 0
LB 0 Hz
GB 0
PC 1.00

^{1}H NMR of 8.0 mM peptide **1a** + 8.0 mM peptide *ent*-**1a** in D_2O at 600 MHz and 298 K

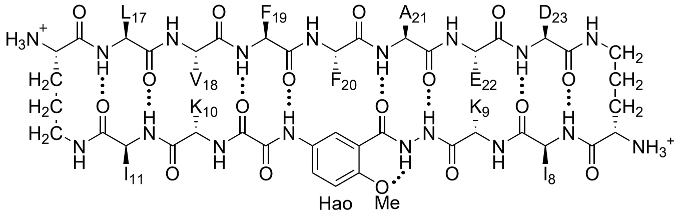

peptide **1a**

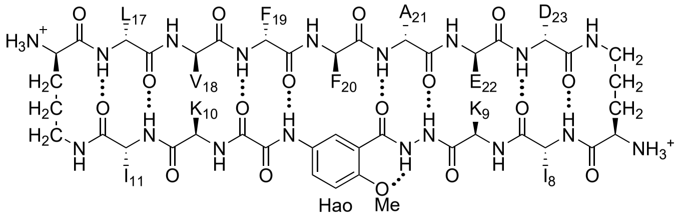

ent-**1a**

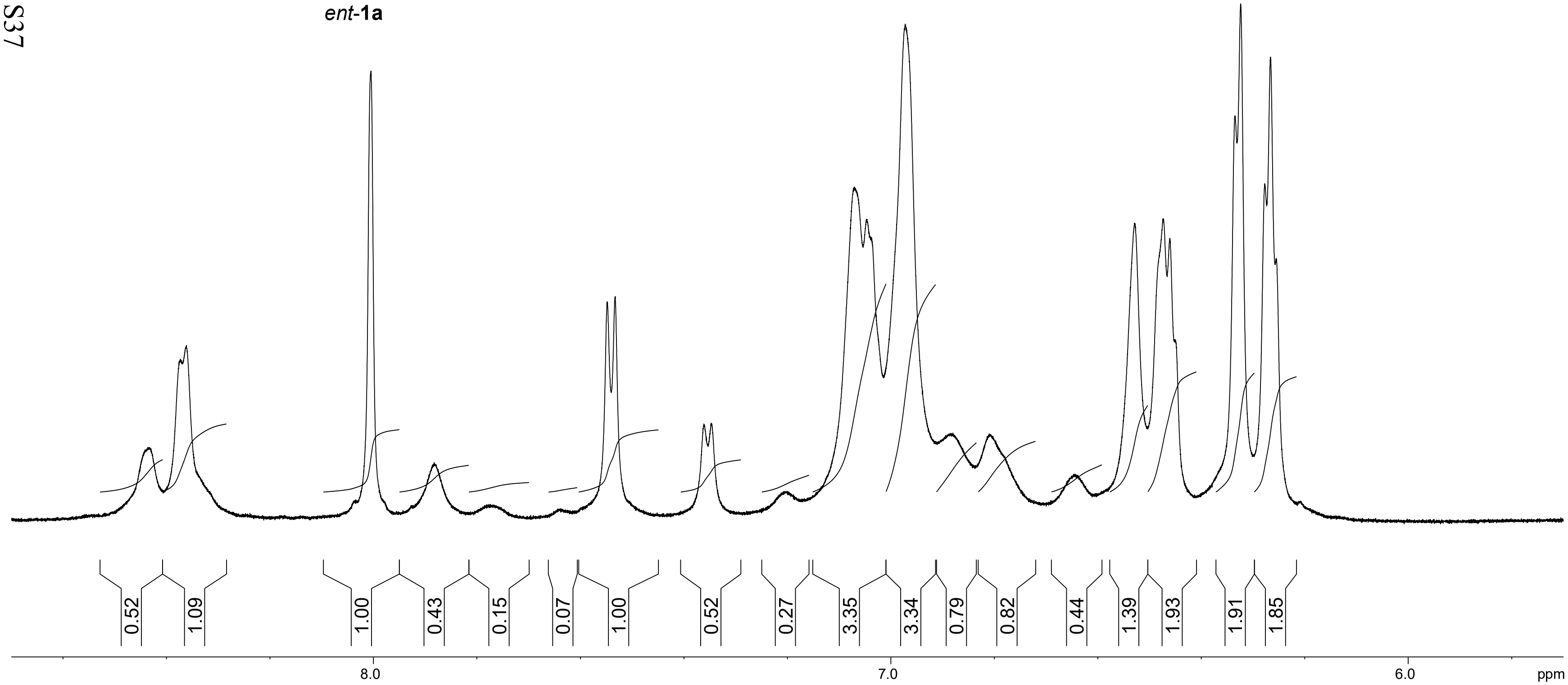

F2 - Acquisition Parameters
Date_ 20211014
Time 23.27
INSTRUM av600
PROBHD 5 mm CPBBO BB-
PULPROG zg30
TD 98074
SOLVENT D2O
NS 512
DS 2
SWH 6602.113 Hz
FIDRES 0.067318 Hz
AQ 7.4274712 sec
RG 10
DW 75.733 usec
DE 12.12 usec
TE 298.0 K
D1 0.10000000 sec
TD0 1

======== CHANNEL f1 ========
SFO1 600.1328835 MHz
NUC1 1H
P1 10.88 usec
PLW1 30.00000000 W

F2 - Processing parameters
SI 65536
SF 600.1300079 MHz
WDW no
SSB 0
LB 0 Hz
GB 0
PC 1.00

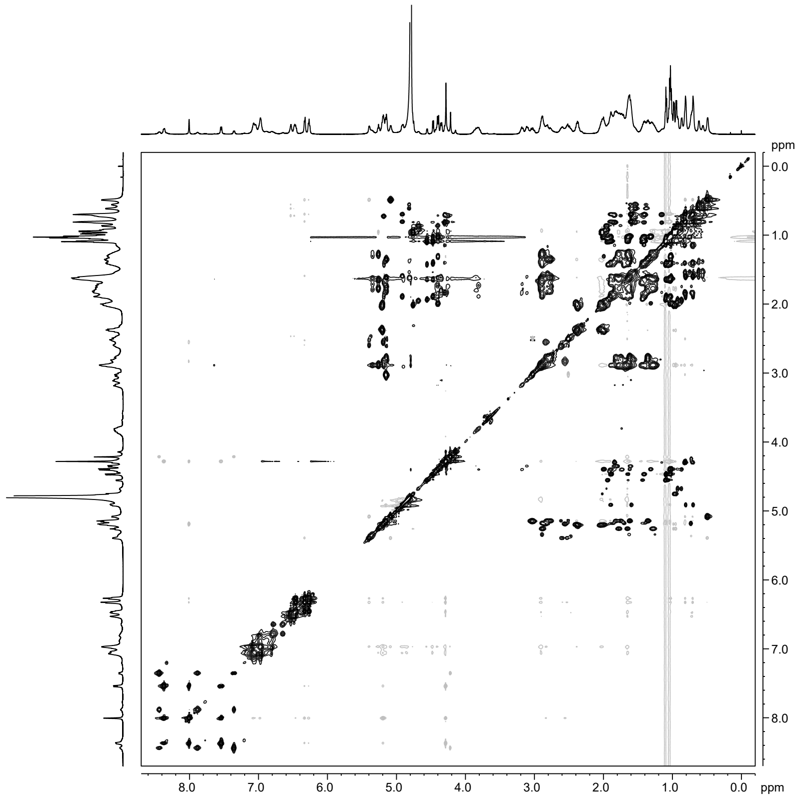

8.0 mM peptide **1a** + 8.0 mM *ent*-**1a**
in D_2O, 150-ms mixing time, 298 K
600 MHz TOCSY spectrum
presaturation suppression of the HOD peak

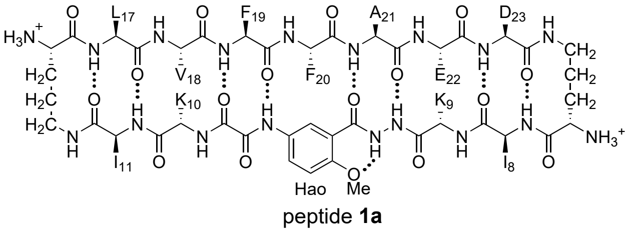

peptide **1a**

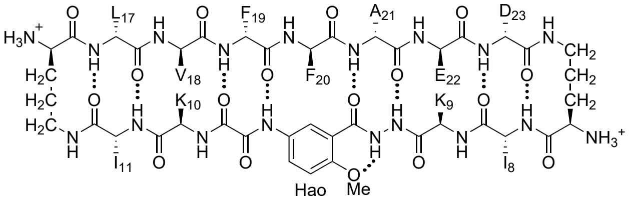

ent-**1a**

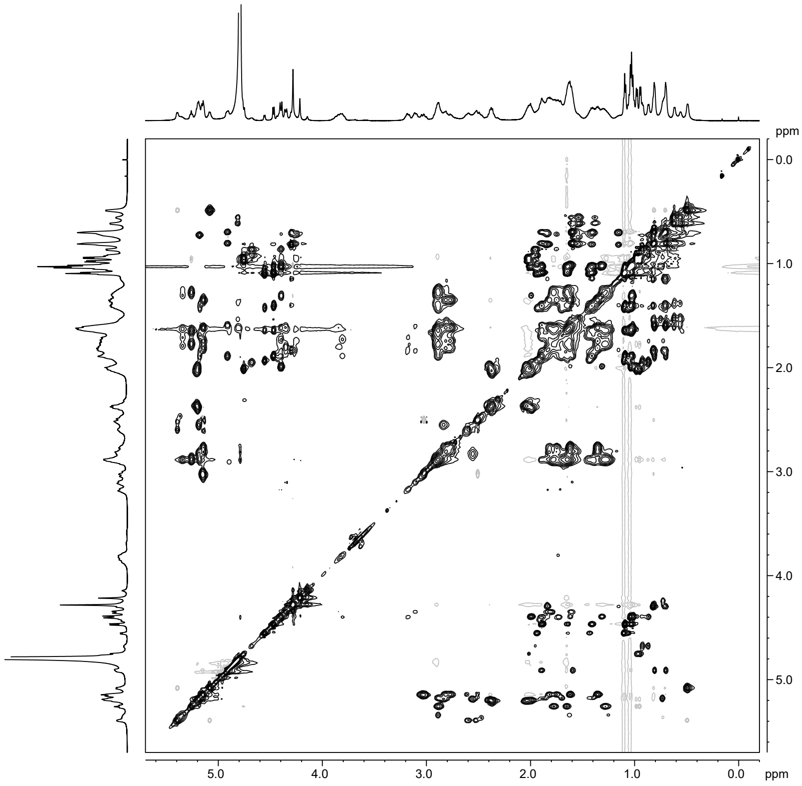

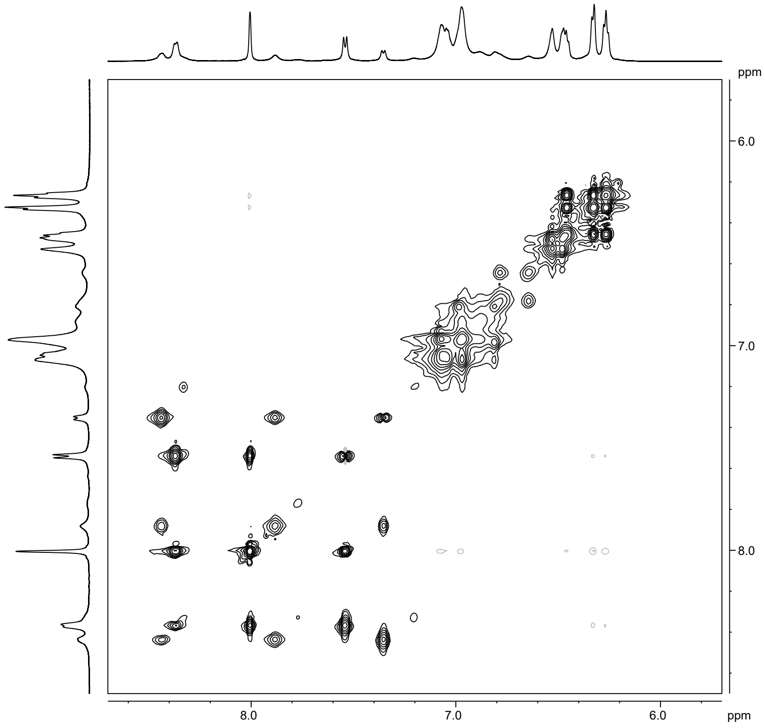

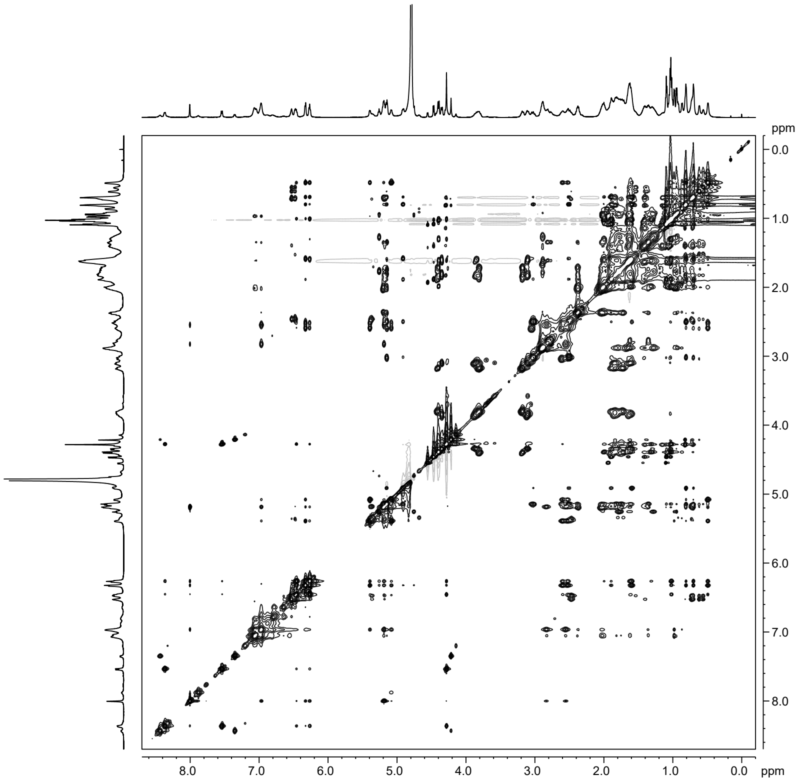

8.0 mM peptide **1a** + 8.0 mM *ent*-**1a**
in D_2O, 200-ms mixing time, 298 K
600 MHz NOESY spectrum
presaturation suppression of the HOD peak

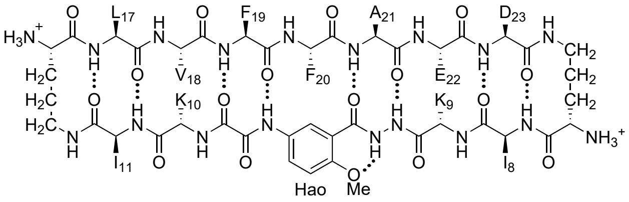

peptide **1a**

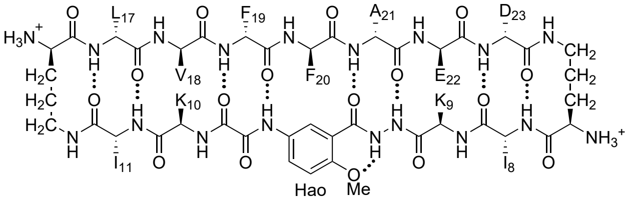

ent-**1a**

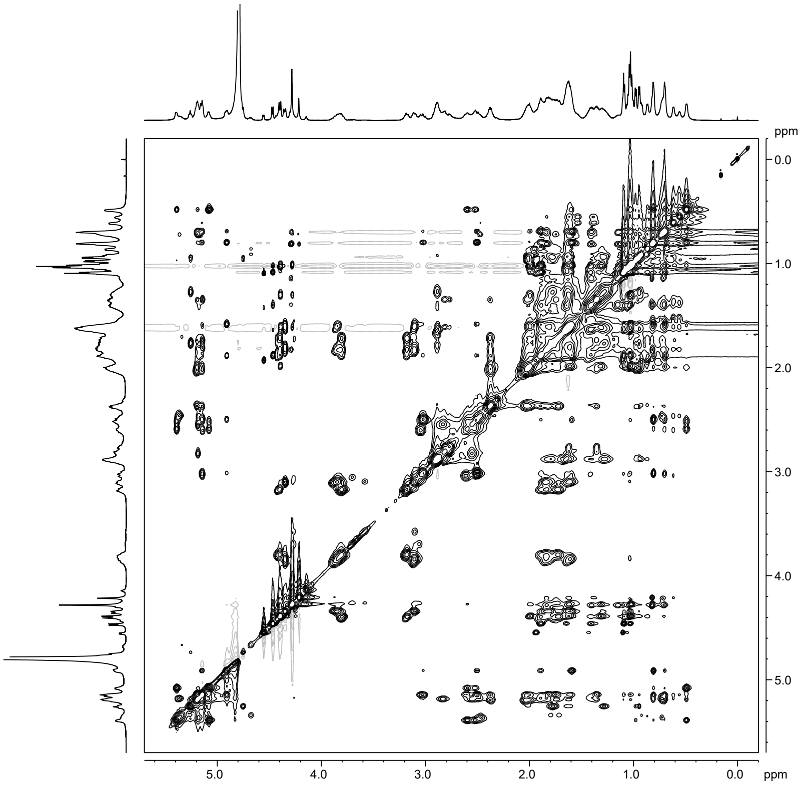

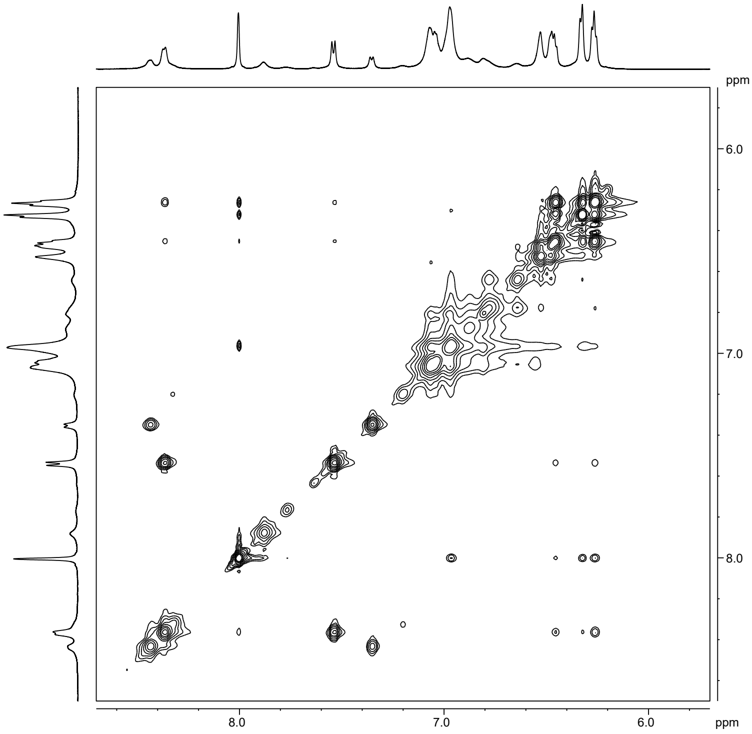

8.0 mM peptide **1a** + 8.0 mM *ent*-**1a**
in D_2O, 200-ms mixing time, 298 K
600 MHz NOESY spectrum
presaturation suppression of the HOD peak

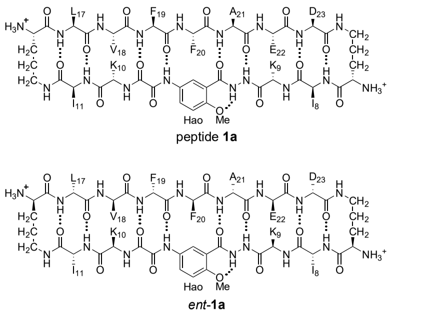

homochiral tetramer associated resonances

heterochiral tetramer associated resonances

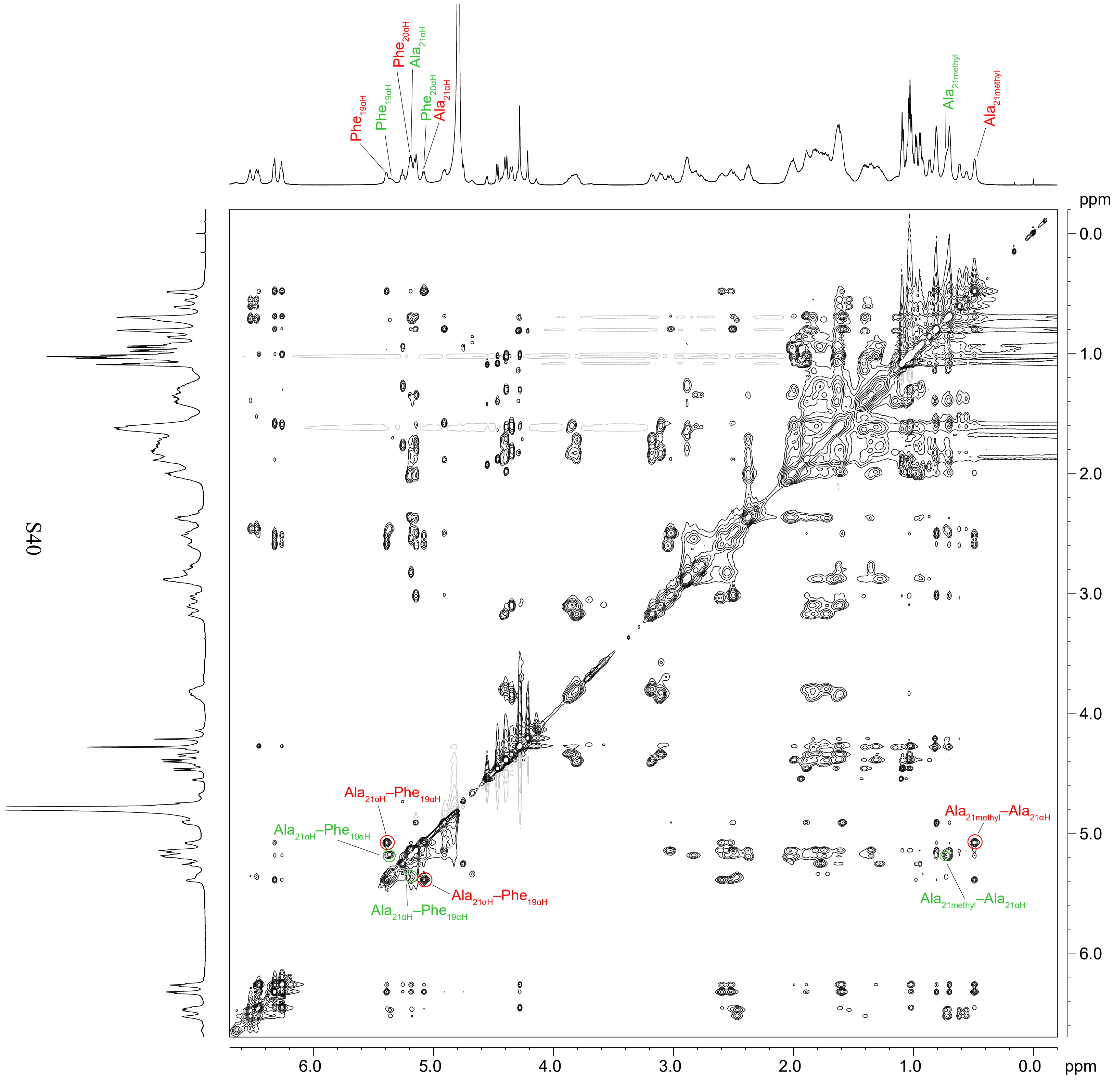

^{1}H NMR of 8.0 mM peptide **3a** + 8.0 mM peptide *ent*-**1a** in D_2O at 600 MHz and 298 K

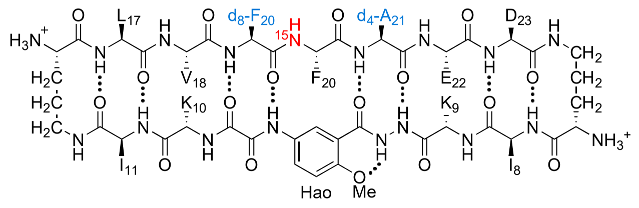

peptide **3a**

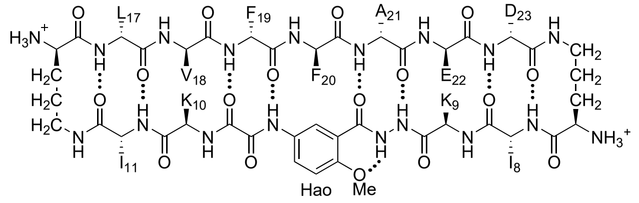

ent-**1a**

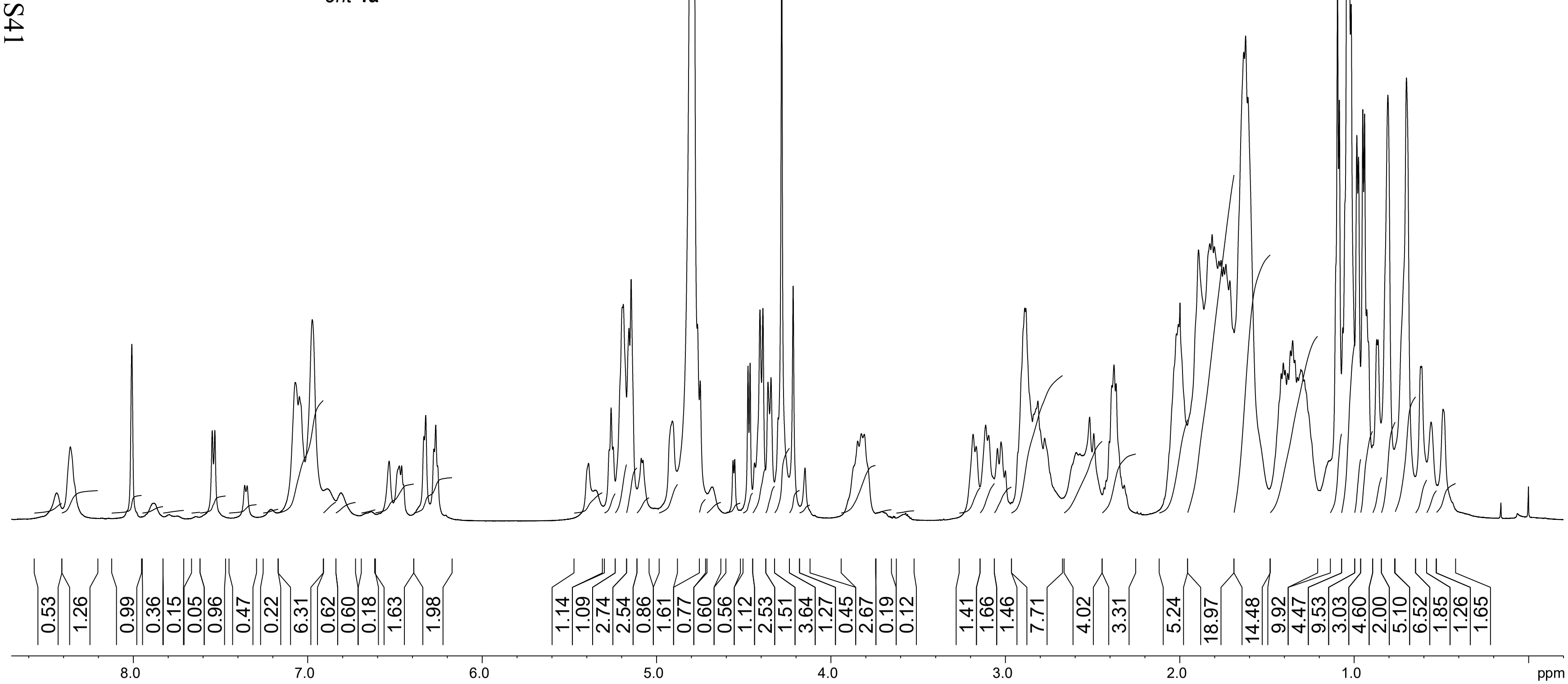

F2 - Acquisition Parameters
Date_ 20211221
Time 23.48
INSTRUM av600
PROBHD 5 mm CPBBO BB-
PULPROG zg30
TD 98074
SOLVENT D2O
NS 512
DS 2
SWH 6493.506 Hz
FIDRES 0.066210 Hz
AQ 7.5516982 sec
RG 10
DW 77.000 usec
DE 12.41 usec
TE 298.0 K
D1 0.10000000 sec
TD0 1

======== CHANNEL f1 ========
SFO1 600.1328840 MHz
NUC1 1H
P1 10.38 usec
PLW1 30.00000000 W

F2 - Processing parameters
SI 65536
SF 600.1300084 MHz
WDW EM
SSB 0
LB 0.30 Hz
GB 0
PC 1.00

^{1}H NMR of 8.0 mM peptide **3a** + 8.0 mM peptide *ent*-**1a** in D_2O at 600 MHz and 298 K

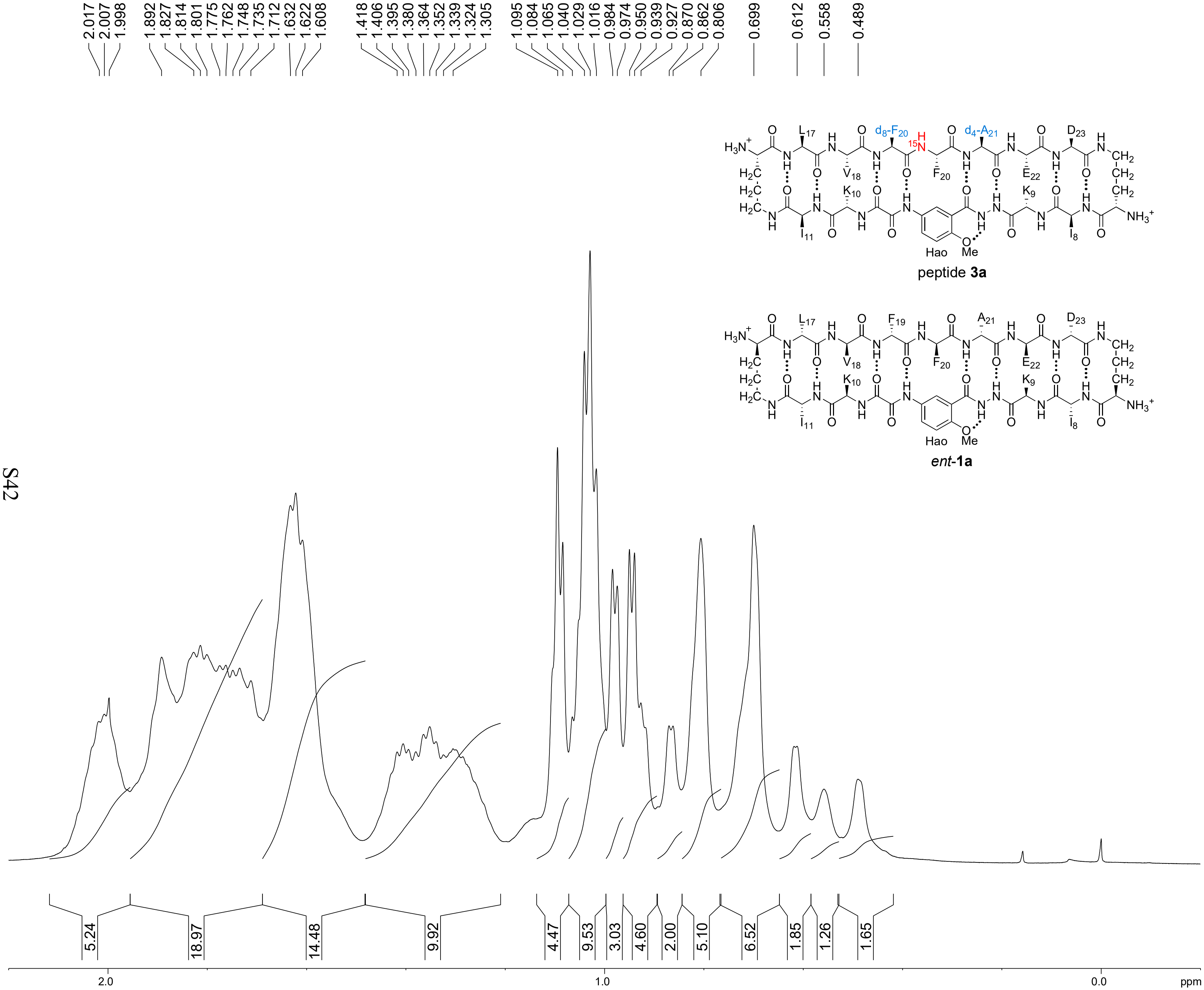

F2 - Acquisition Parameters
Date_ 20211221
Time 23.48
INSTRUM av600
PROBHD 5 mm CPBBO BB-
PULPROG zg30
TD 98074
SOLVENT D2O
NS 512
DS 2
SWH 6493.506 Hz
FIDRES 0.066210 Hz
AQ 7.5516982 sec
RG 10
DW 77.000 usec
DE 12.41 usec
TE 298.0 K
D1 0.10000000 sec
TD0 1

======== CHANNEL f1 ========
SFO1 600.1328840 MHz
NUC1 1H
P1 10.38 usec
PLW1 30.00000000 W

F2 - Processing parameters
SI 65536
SF 600.1300084 MHz
WDW EM
SSB 0
LB 0.30 Hz
GB 0
PC 1.00

^{1}H NMR of 8.0 mM peptide **3a** + 8.0 mM peptide *ent*-**1a** in D_2O at 600 MHz and 298 K

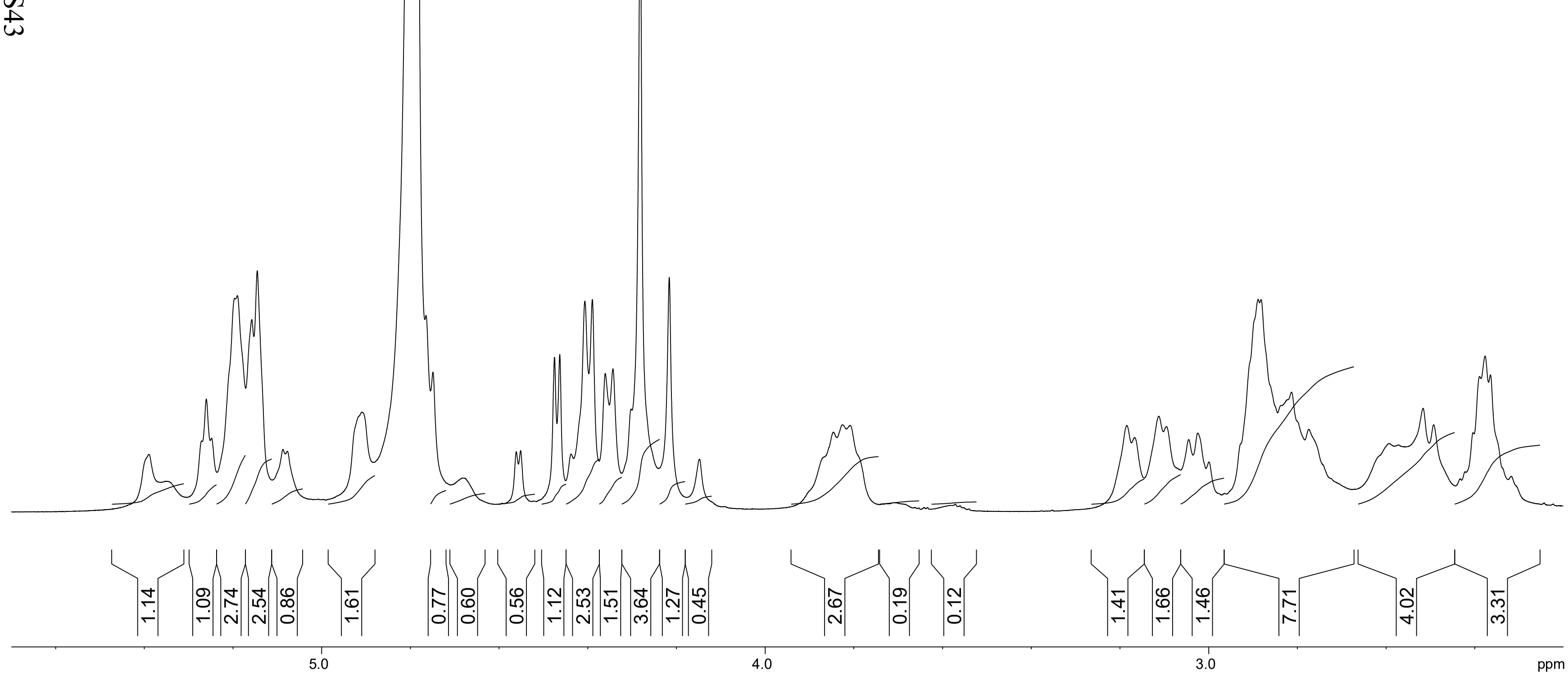

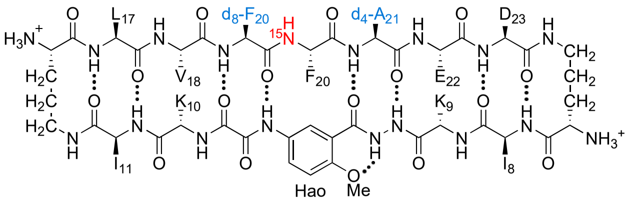

peptide **3a**

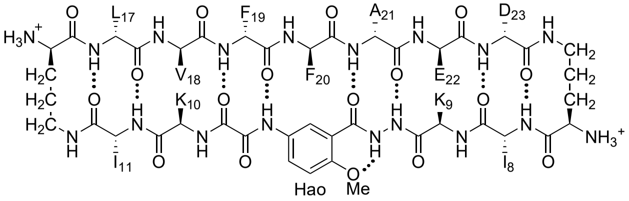

ent-**1a**

F2 - Acquisition Parameters
Date_ 20211221
Time 23.48
INSTRUM av600
PROBHD 5 mm CPBBO BB-
PULPROG zg30
TD 98074
SOLVENT D2O
NS 512
DS 2
SWH 6493.506 Hz
FIDRES 0.066210 Hz
AQ 7.5516982 sec
RG 10
DW 77.000 usec
DE 12.41 usec
TE 298.0 K
D1 0.10000000 sec
TD0 1

======== CHANNEL f1 ========
SFO1 600.1328840 MHz
NUC1 1H
P1 10.38 usec
PLW1 30.00000000 W

F2 - Processing parameters
SI 65536
SF 600.1300084 MHz
WDW EM
SSB 0
LB 0.30 Hz
GB 0
PC 1.00

^{1}H NMR of 8.0 mM peptide **3a** + 8.0 mM peptide *ent*-**1a** in D_2O at 600 MHz and 298 K

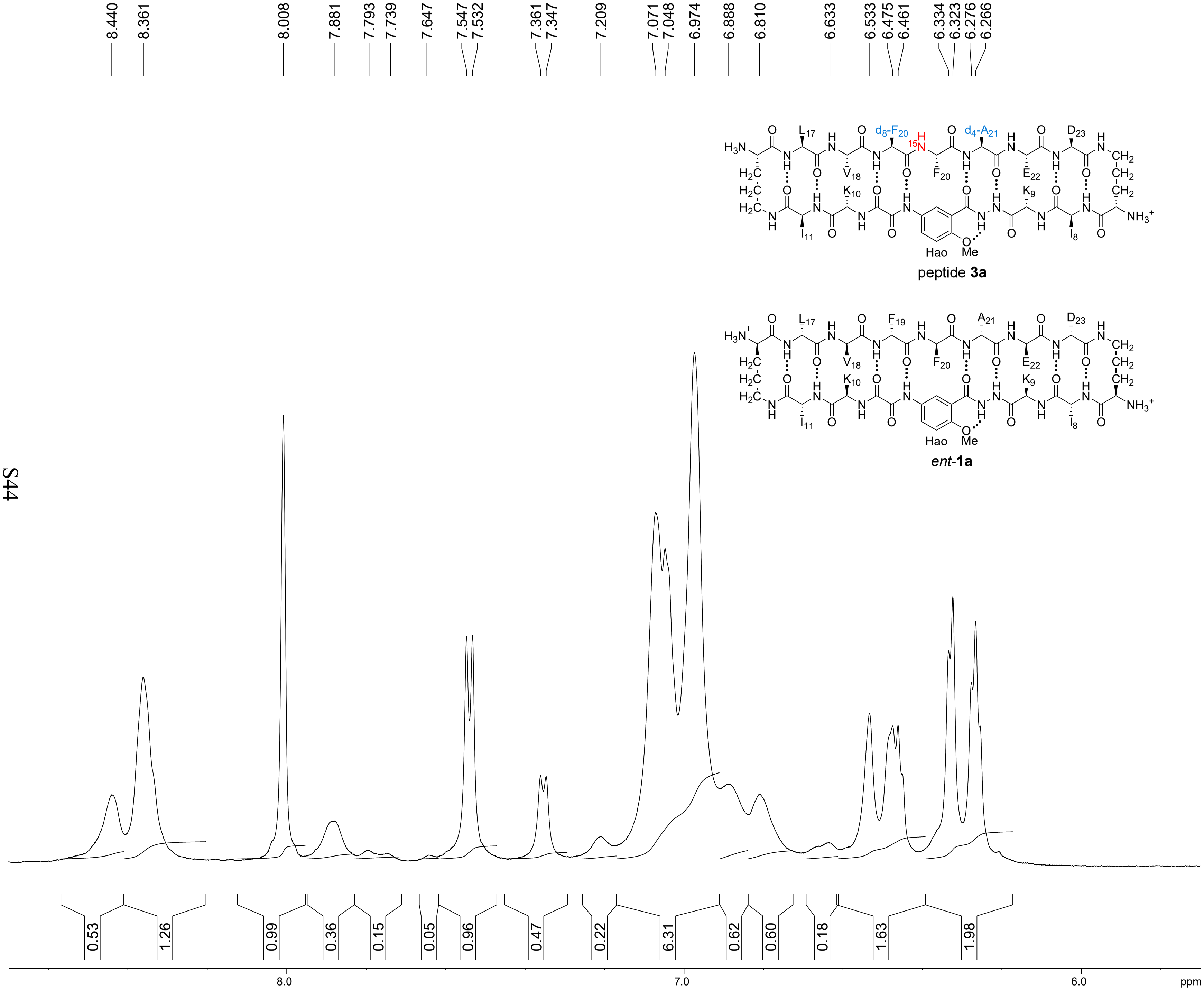

F2 - Acquisition Parameters
Date_ 20211221
Time 23.48
INSTRUM av600
PROBHD 5 mm CPBBO BB-
PULPROG zg30
TD 98074
SOLVENT D2O
NS 512
DS 2
SWH 6493.506 Hz
FIDRES 0.066210 Hz
AQ 7.5516982 sec
RG 10
DW 77.000 usec
DE 12.41 usec
TE 298.0 K
D1 0.10000000 sec
TD0 1

======== CHANNEL f1 ========
SFO1 600.1328840 MHz
NUC1 1H
P1 10.38 usec
PLW1 30.00000000 W

F2 - Processing parameters
SI 65536
SF 600.1300084 MHz
WDW EM
SSB 0
LB 0.30 Hz
GB 0
PC 1.00

8.0 mM peptide **3a** + 8.0 mM *ent*-**1a**
in D_2O, 150-ms mixing time, 298 K
600 MHz TOCSY spectrum
presaturation suppression of the HOD peak

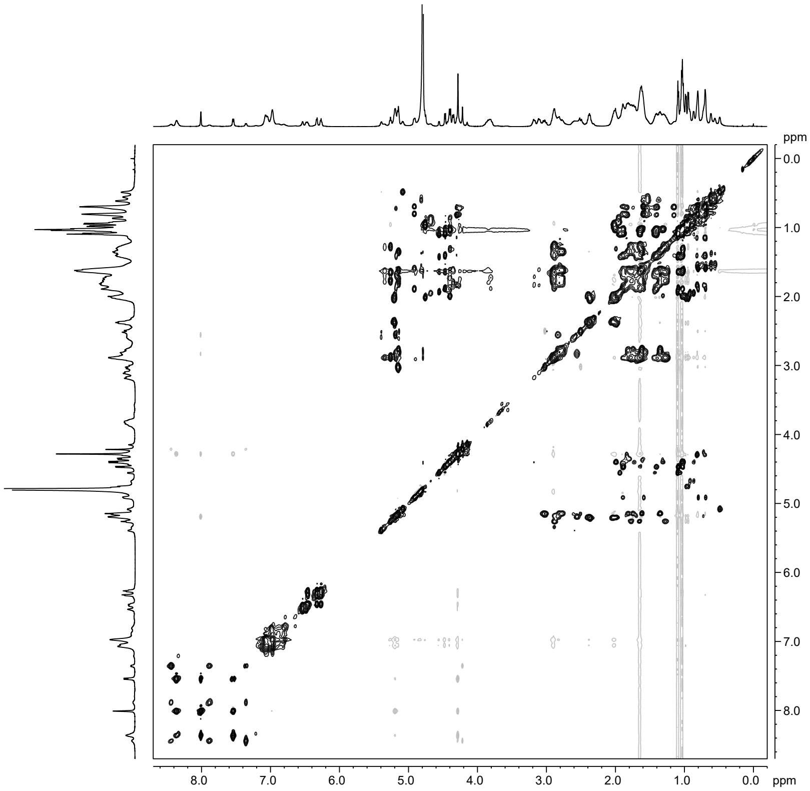

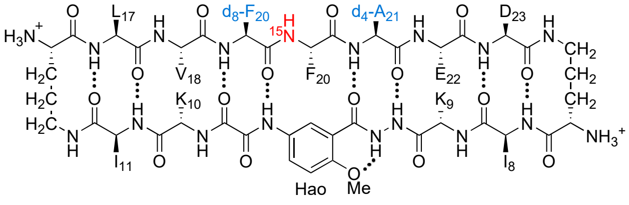

peptide **3a**

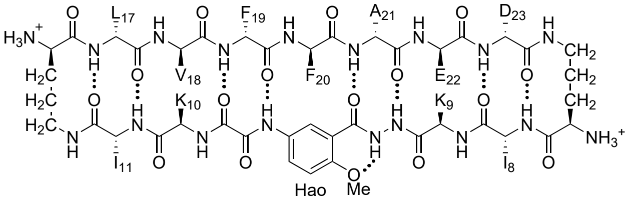

ent-**1a**

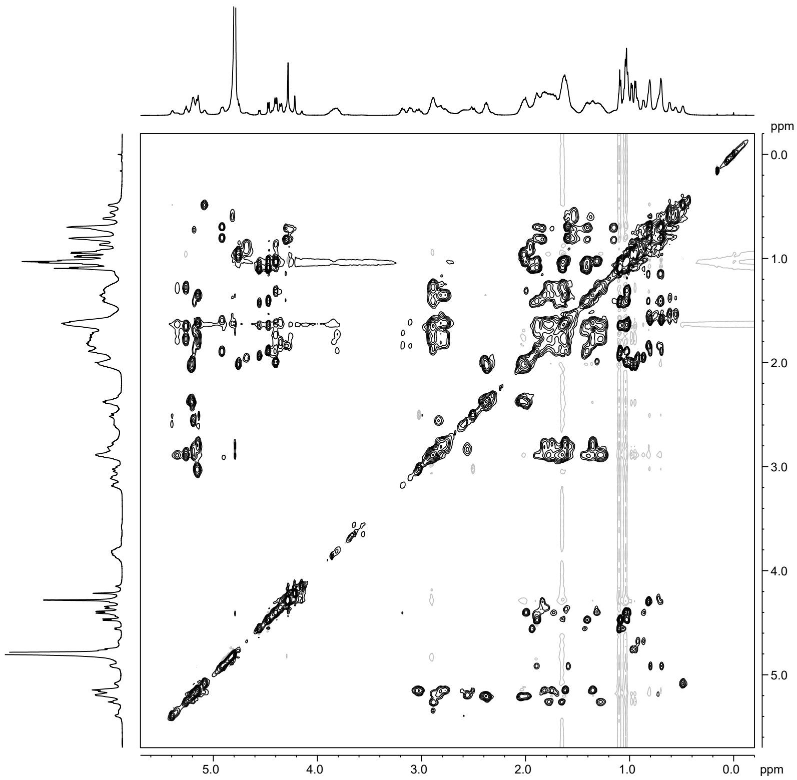

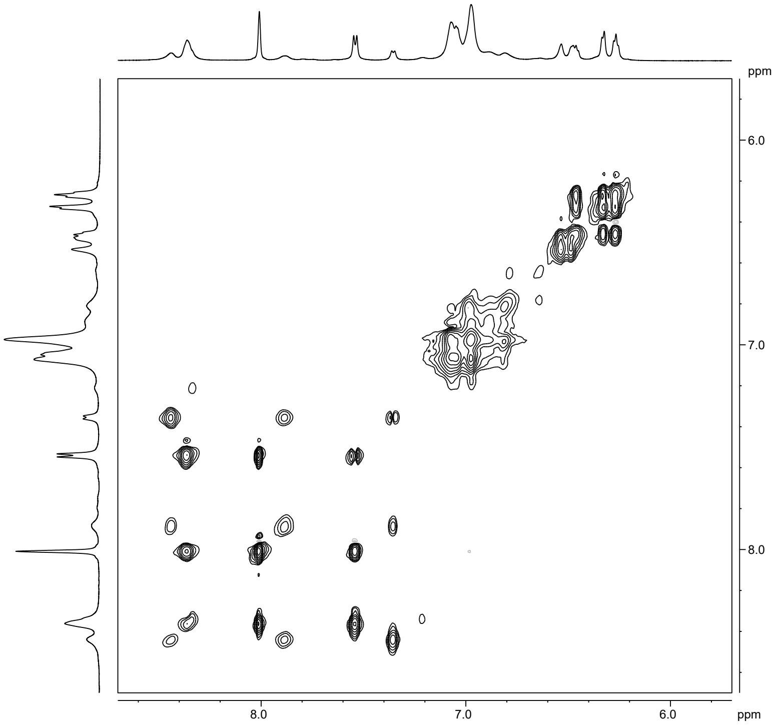

8.0 mM peptide **3a** + 8.0 mM *ent*-**1a**
in D_2O, 200-ms mixing time, 298 K
600 MHz NOESY spectrum
presaturation suppression of the HOD peak

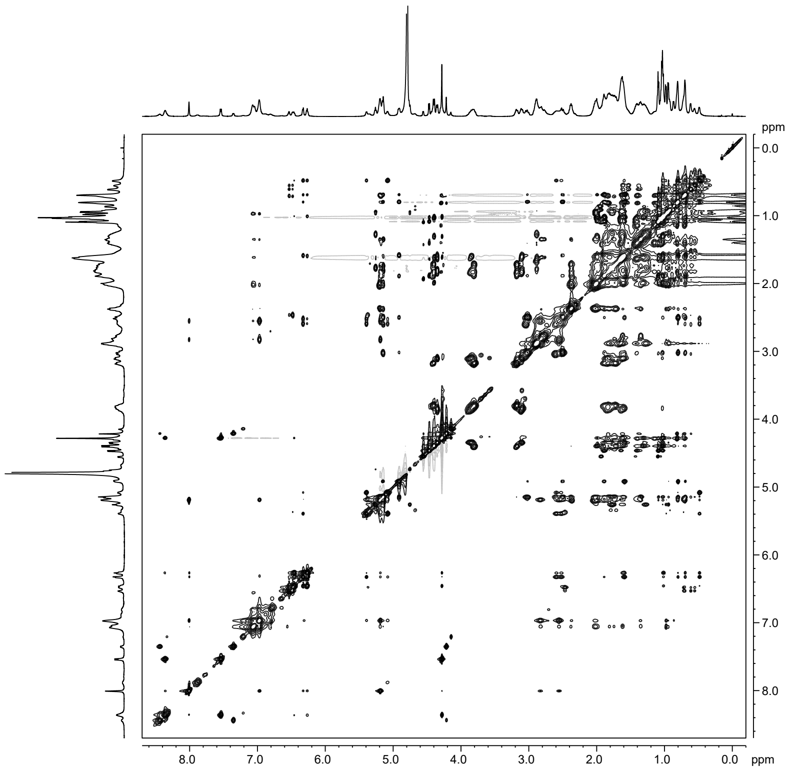

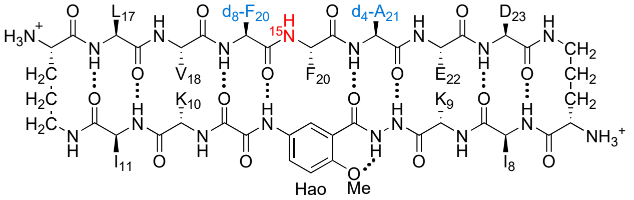

peptide **3a**

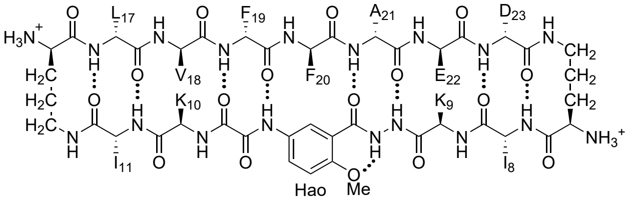

ent-**1a**

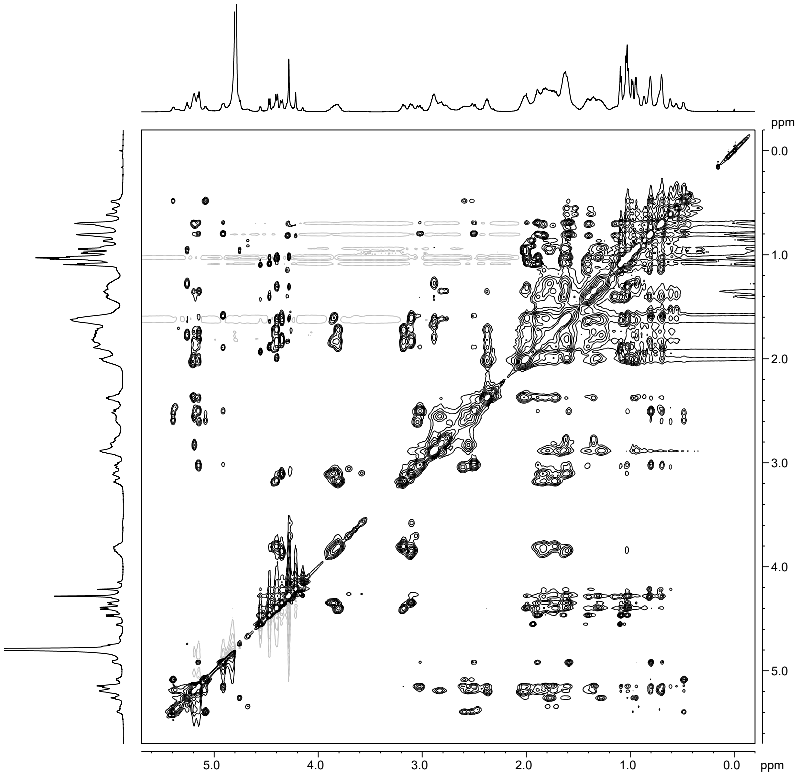

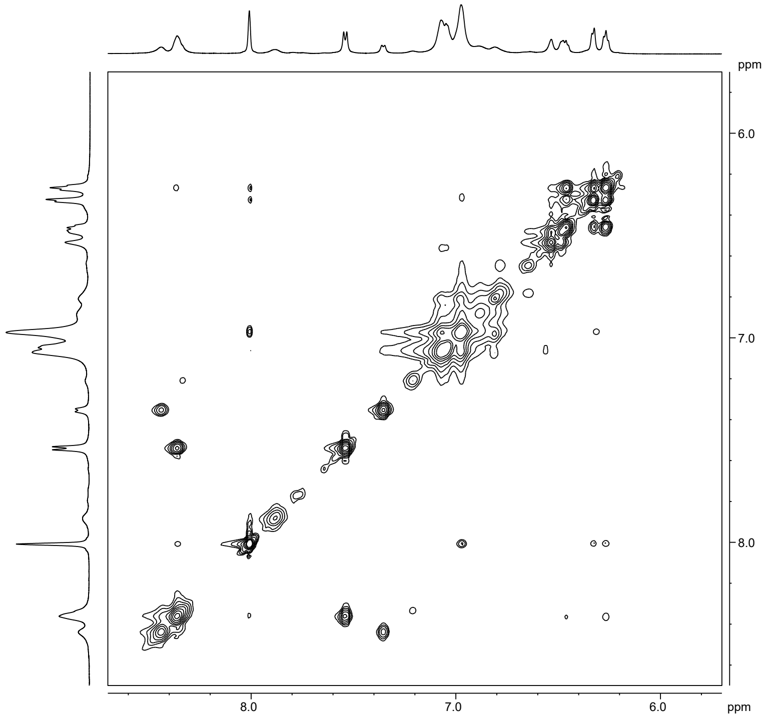

^1H NMR of 4.0 mM peptide **1b** in D_2O at 600 MHz and 298 K

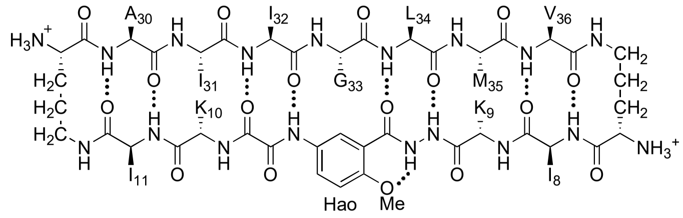

peptide **1b**

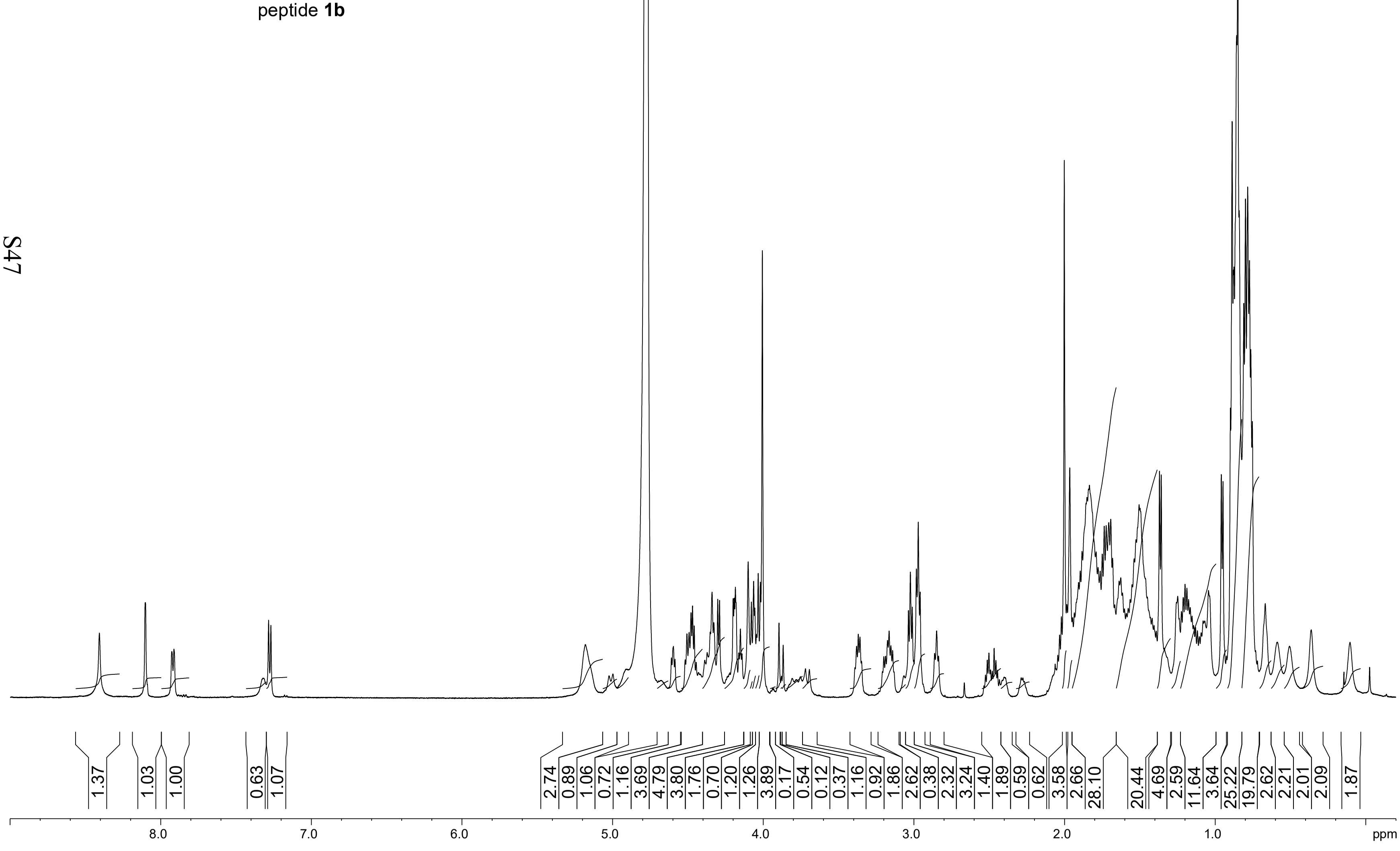

F2 - Acquisition Parameters
Date_ 20220225
Time 10.22
INSTRUM av600
PROBHD 5 mm CPBBO BB-
PULPROG zg30
TD 98074
SOLVENT D2O
NS 256
DS 2
SWH 6361.323 Hz
FIDRES 0.064862 Hz
AQ 7.7086163 sec
RG 10
DW 78.600 usec
DE 12.82 usec
TE 298.0 K
D1 0.10000000 sec
TD0 1

======== CHANNEL f1 ========
SFO1 600.1328866 MHz
NUC1 1H
P1 10.00 usec
PLW1 30.00000000 W

F2 - Processing parameters
SI 65536
SF 600.1300193 MHz
WDW EM
SSB 0
LB 0.30 Hz
GB 0
PC 1.00

^1H NMR of 4.0 mM peptide **1b** in D_2O at 600 MHz and 298 K

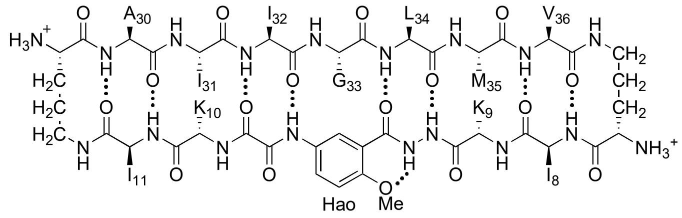

peptide **1b**

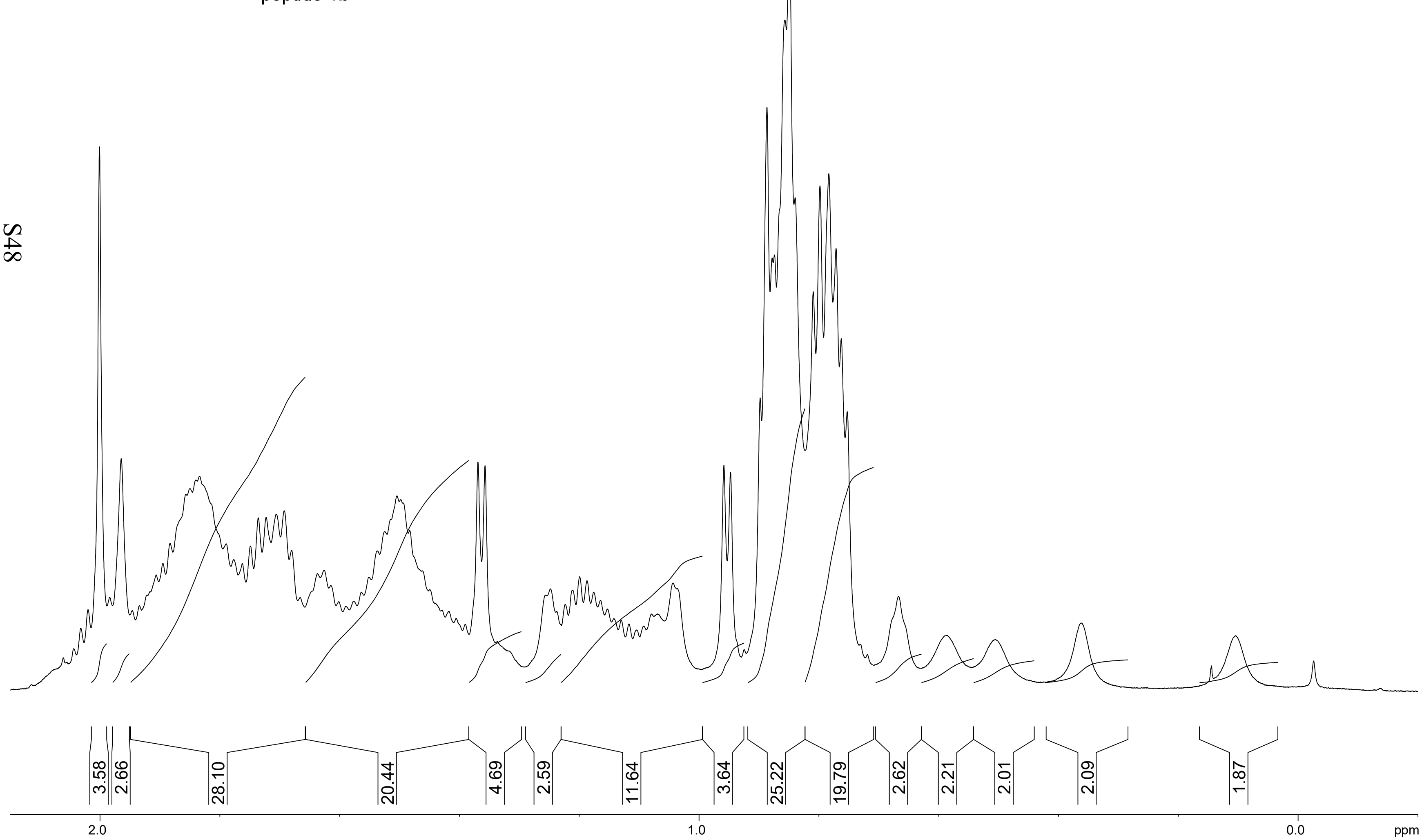

F2 - Acquisition Parameters
Date_ 20220225
Time 10.22
INSTRUM av600
PROBHD 5 mm CPBBO BB-
PULPROG zg30
TD 98074
SOLVENT D2O
NS 256
DS 2
SWH 6361.323 Hz
FIDRES 0.064862 Hz
AQ 7.7086163 sec
RG 10
DW 78.600 usec
DE 12.82 usec
TE 298.0 K
D1 0.10000000 sec
TD0 1

======== CHANNEL f1 ========
SFO1 600.1328866 MHz
NUC1 1H
P1 10.00 usec
PLW1 30.00000000 W

F2 - Processing parameters
SI 65536
SF 600.1300193 MHz
WDW EM
SSB 0
LB 0.30 Hz
GB 0
PC 1.00

^{1}H NMR of 4.0 mM peptide **1b** in D_2O at 600 MHz and 298 K

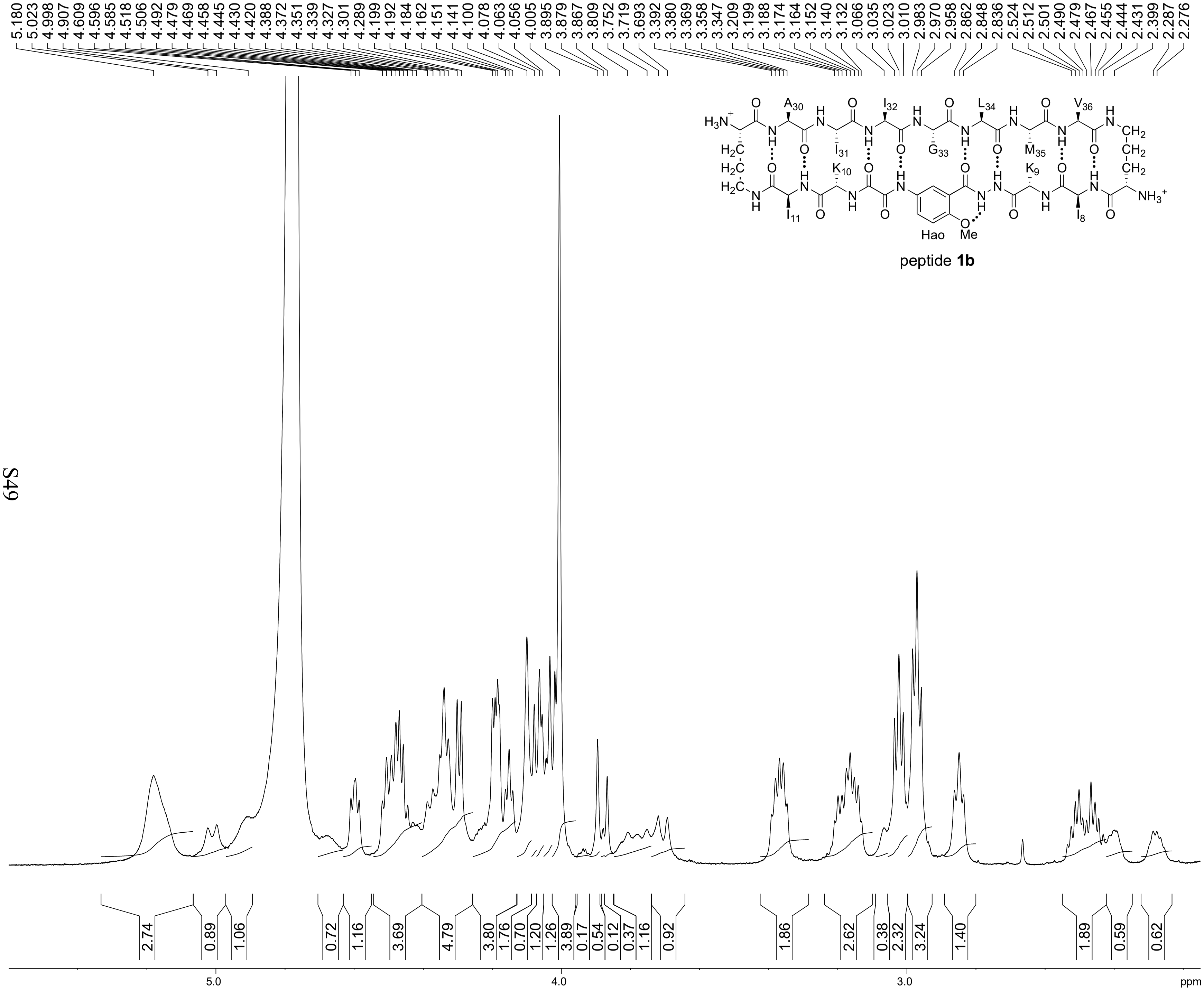

F2 - Acquisition Parameters
Date_ 20220225
Time 10.22
INSTRUM av600
PROBHD 5 mm CPBBO BB-
PULPROG zg30
TD 98074
SOLVENT D2O
NS 256
DS 2
SWH 6361.323 Hz
FIDRES 0.064862 Hz
AQ 7.7086163 sec
RG 10
DW 78.600 usec
DE 12.82 usec
TE 298.0 K
D1 0.10000000 sec
TD0 1

======== CHANNEL f1 ========
SFO1 600.1328866 MHz
NUC1 1H
P1 10.00 usec
PLW1 30.00000000 W

F2 - Processing parameters
SI 65536
SF 600.1300193 MHz
WDW EM
SSB 0
LB 0.30 Hz
GB 0
PC 1.00

^{1}H NMR of 4.0 mM peptide **1b** in D_2O at 600 MHz and 298 K

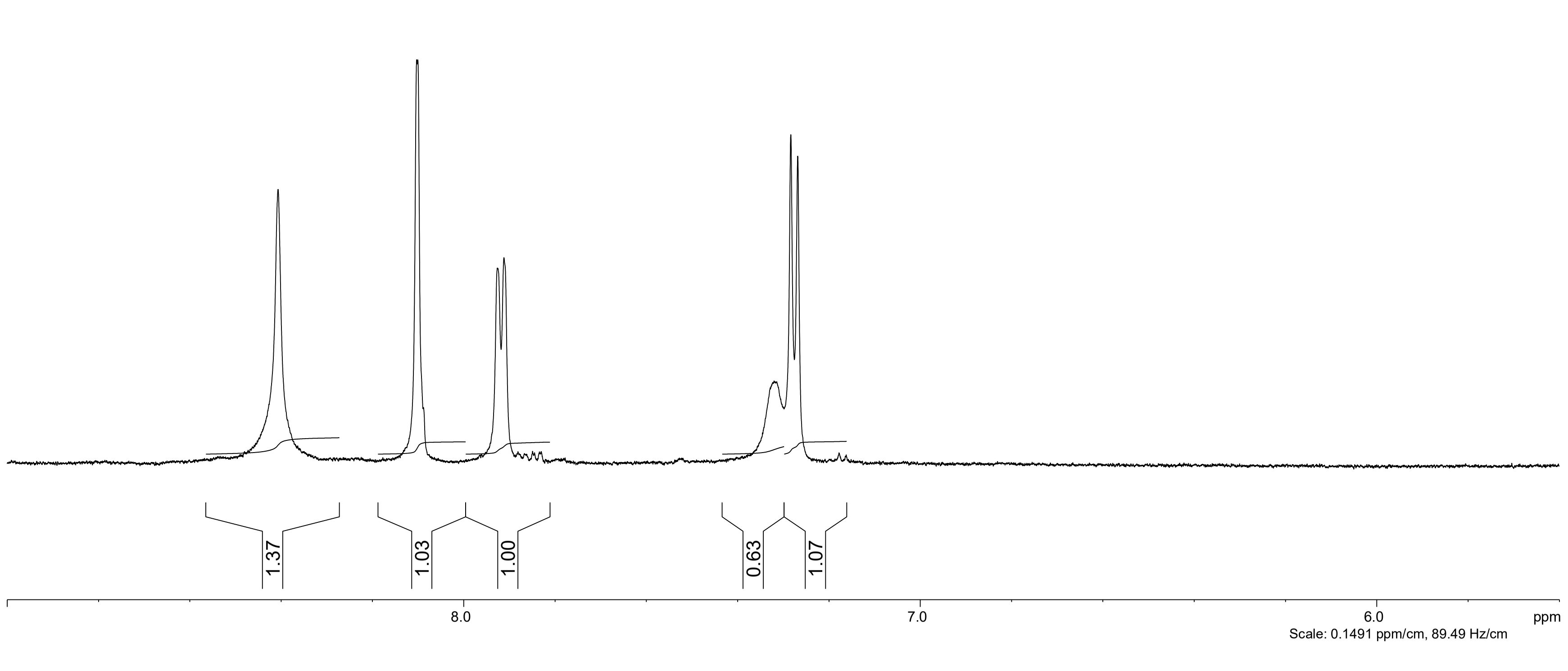

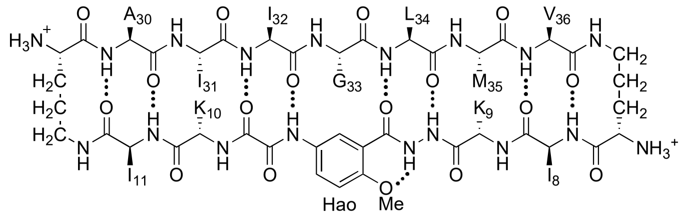

peptide **1b**

F2 - Acquisition Parameters
Date_ 20220225
Time 10.22
INSTRUM av600
PROBHD 5 mm CPBBO BB-
PULPROG zg30
TD 98074
SOLVENT D2O
NS 256
DS 2
SWH 6361.323 Hz
FIDRES 0.064862 Hz
AQ 7.7086163 sec
RG 10
DW 78.600 usec
DE 12.82 usec
TE 298.0 K
D1 0.10000000 sec
TD0 1

======== CHANNEL f1 ========
SFO1 600.1328866 MHz
NUC1 1H
P1 10.00 usec
PLW1 30.00000000 W

F2 - Processing parameters
SI 65536
SF 600.1300193 MHz
WDW EM
SSB 0
LB 0.30 Hz
GB 0
PC 1.00

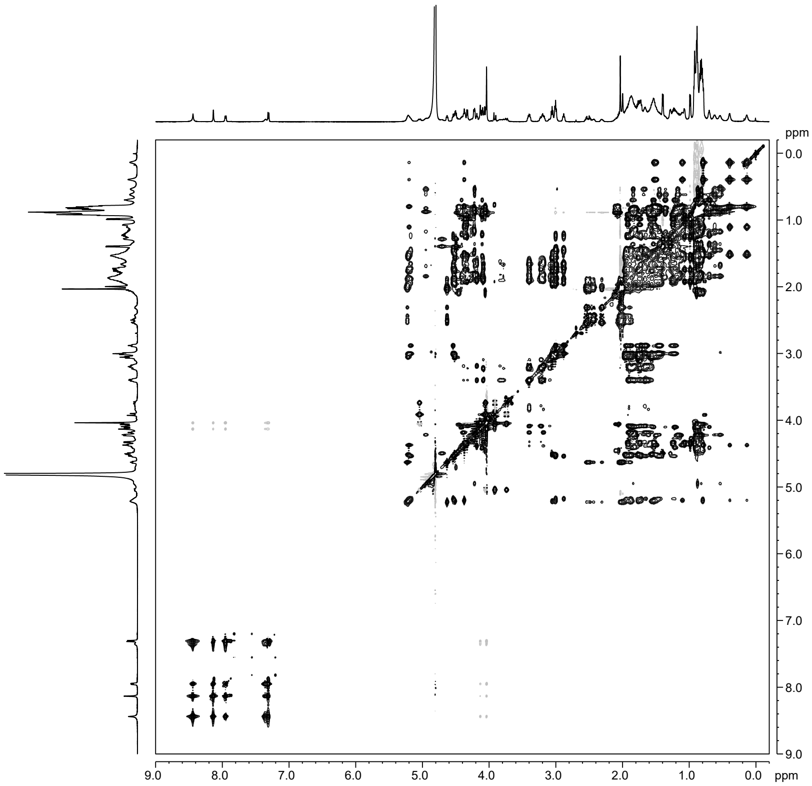

4.0 mM peptide **1b**
in D_2O, 150-ms mixing time, 298 K
600 MHz TOCSY spectrum
presaturation suppression of the HOD peak

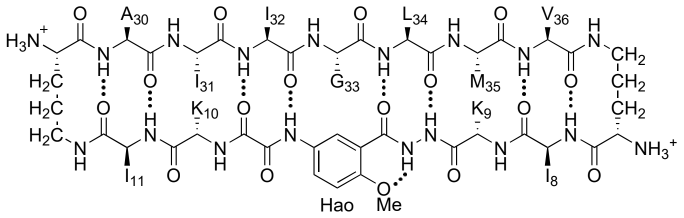

peptide **1b**

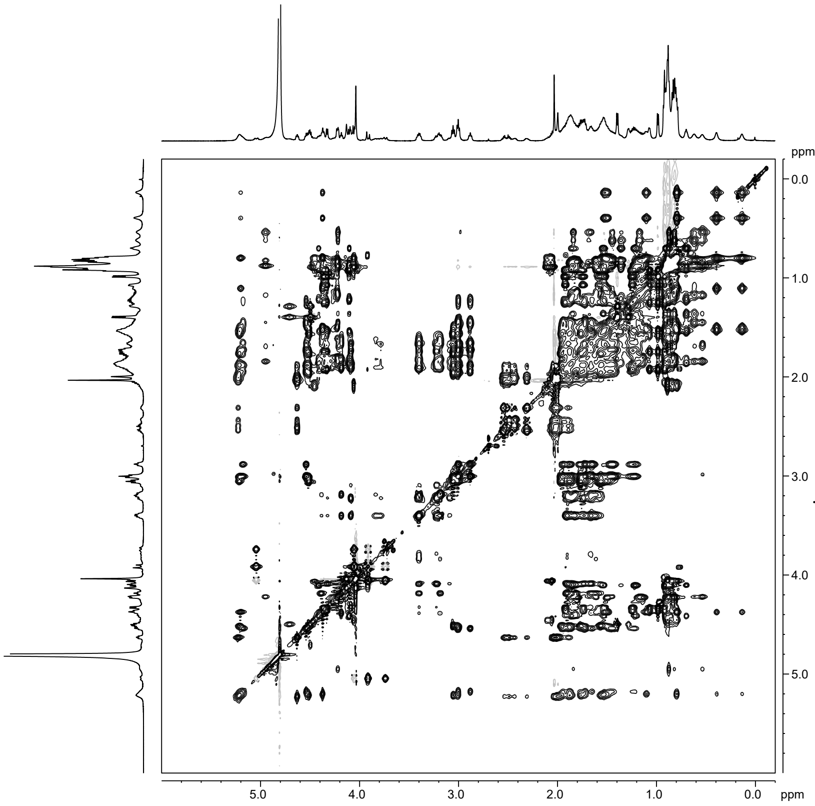

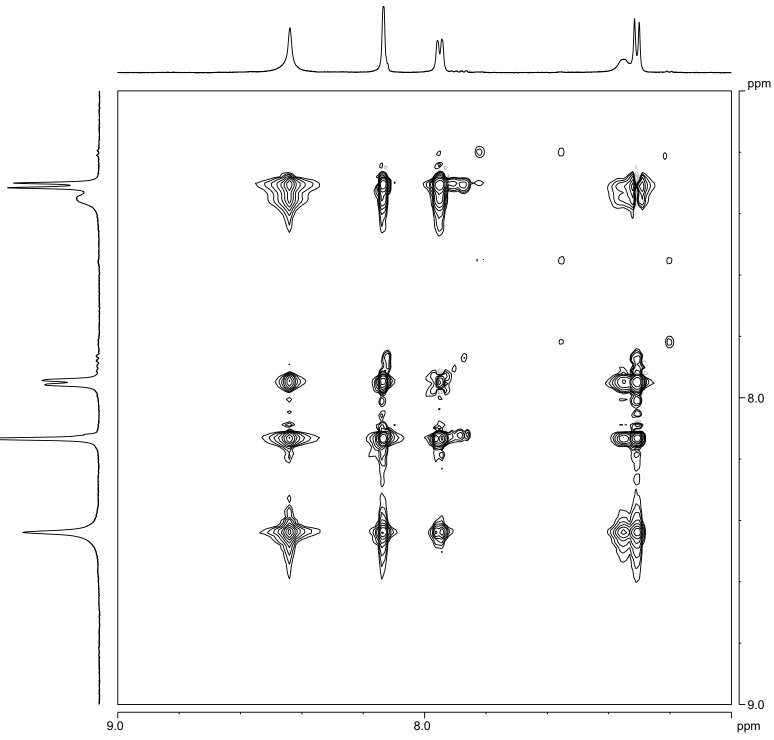

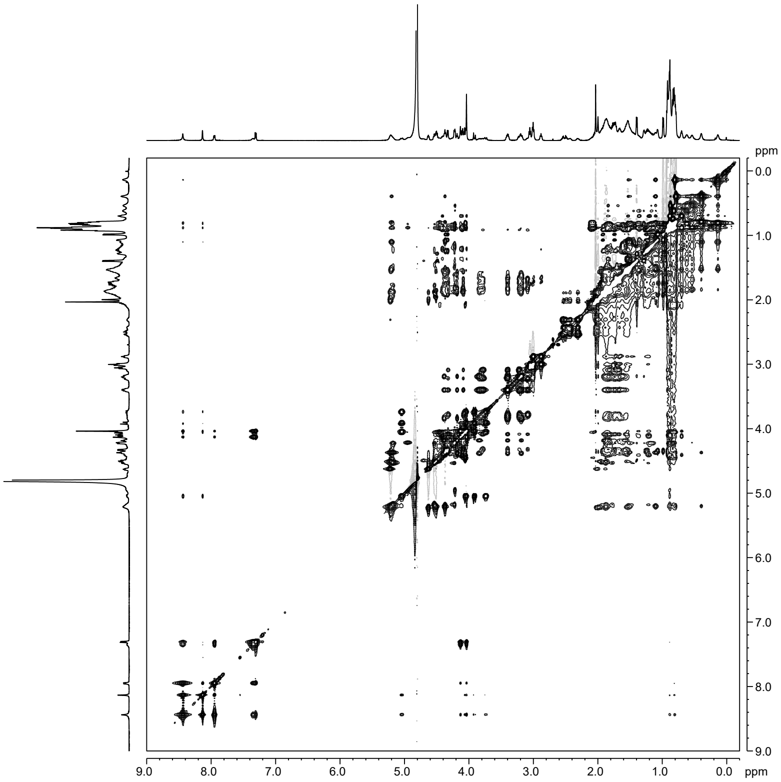

4.0 mM peptide **1b**
in D_2O, 200-ms mixing time, 298 K
600 MHz NOESY spectrum
presaturation suppression of the HOD peak

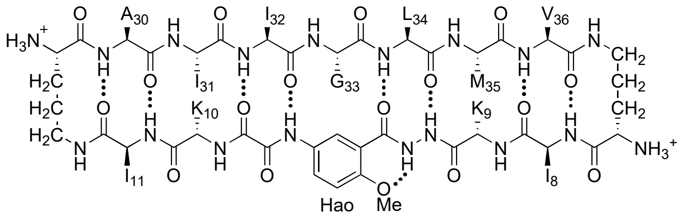

peptide **1b**

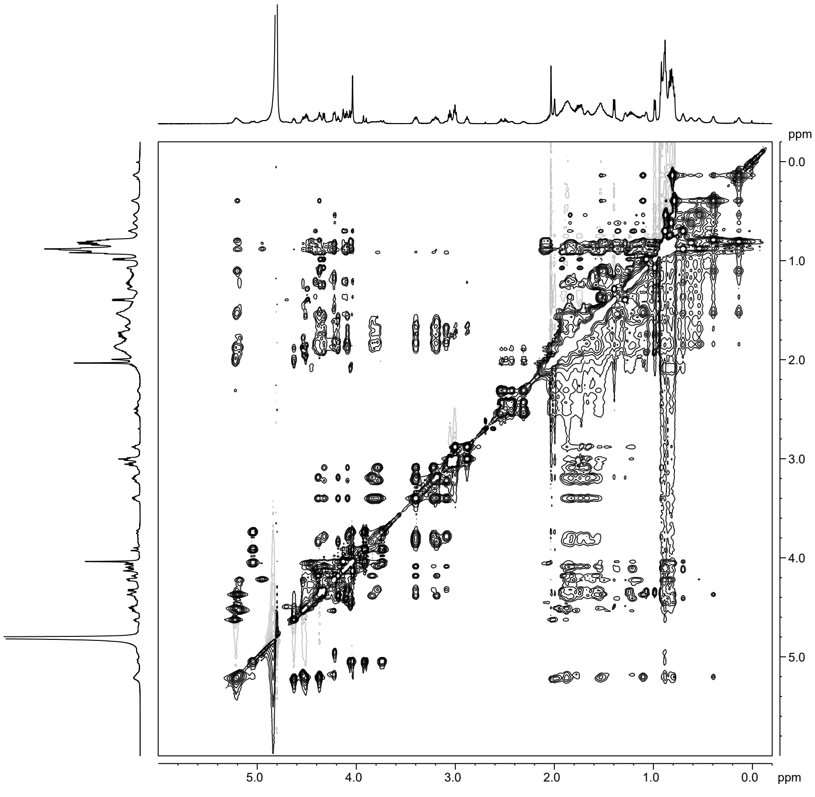

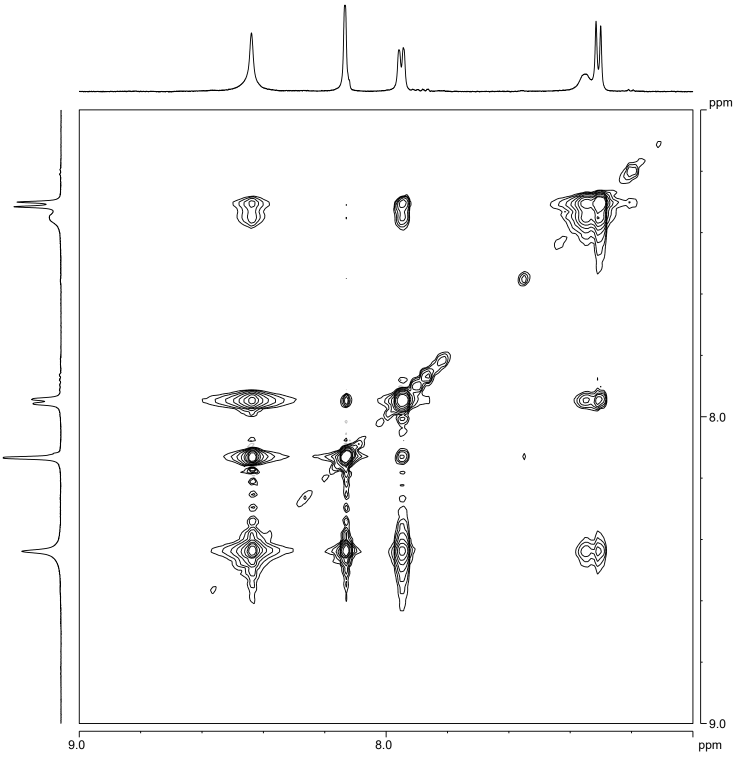

^{1}H NMR of 4.0 mM peptide **1b** + 4.0 mM peptide *ent*-**1b** in D_2O at 600 MHz and 298 K

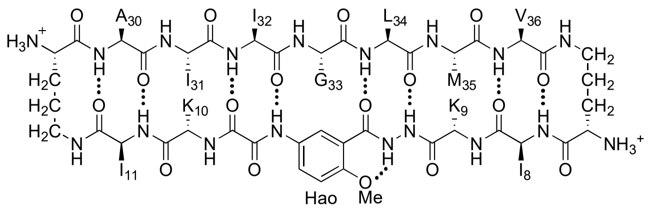

peptide **1b**

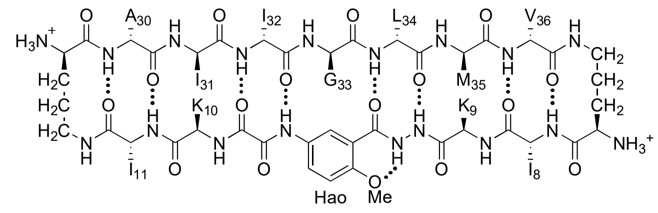

ent-**1b**

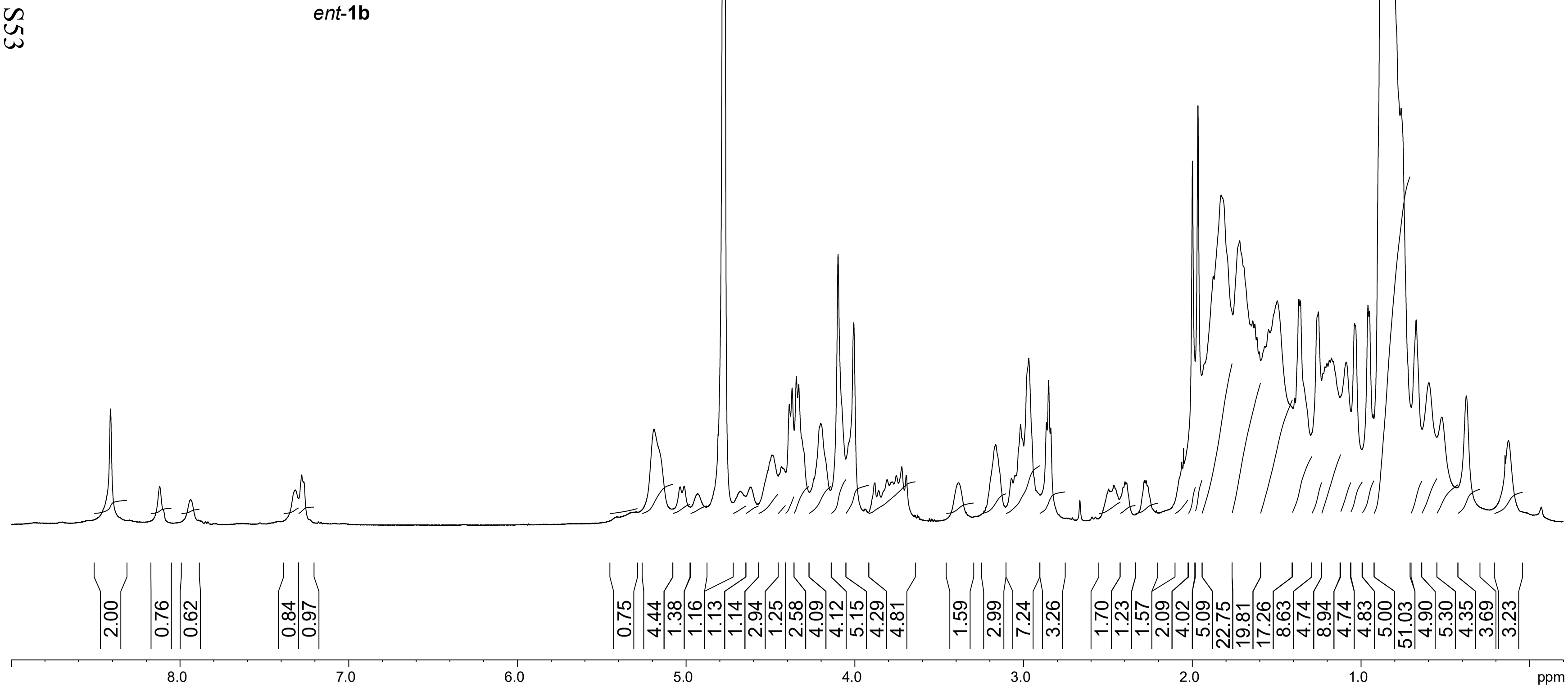

F2 - Acquisition Parameters
Date_ 20220206
Time 23.06
INSTRUM av600
PROBHD 5 mm CPBBO BB-
PULPROG zg30
TD 98074
SOLVENT D2O
NS 512
DS 2
SWH 6487.889 Hz
FIDRES 0.066153 Hz
AQ 7.5582361 sec
RG 10
DW 77.067 usec
DE 13.04 usec
TE 298.0 K
D1 0.10000000 sec
TD0 1

======== CHANNEL f1 ========
SFO1 600.1327096 MHz
NUC1 1H
P1 10.06 usec
PLW1 30.00000000 W

F2 - Processing parameters
SI 65536
SF 600.1300183 MHz
WDW EM
SSB 0
LB 0.30 Hz
GB 0
PC 1.00

^{1}H NMR of 4.0 mM peptide **1b** + 4.0 mM peptide *ent*-**1b** in D_2O at 600 MHz and 298 K

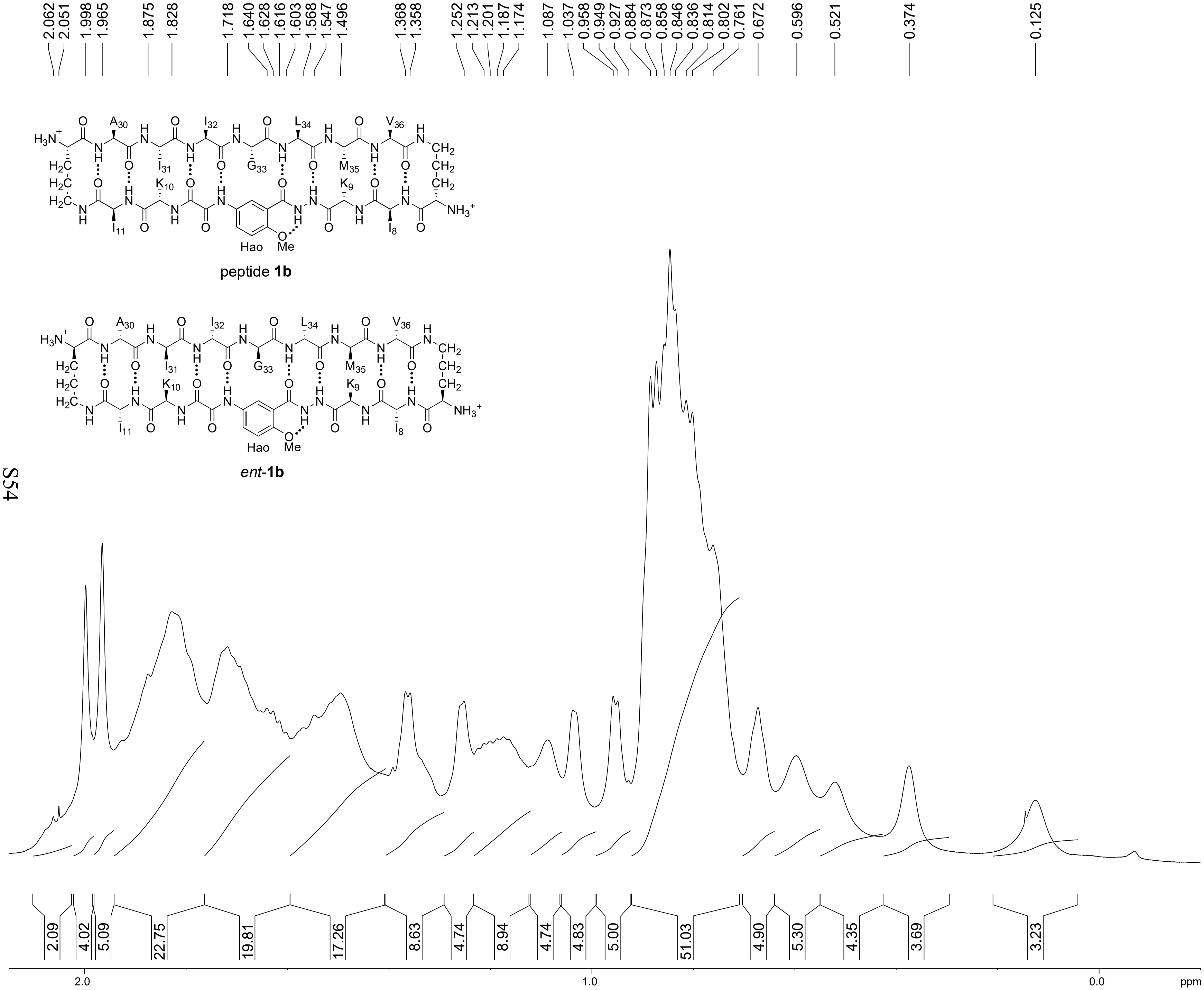

peptide **1b**

ent-**1b**

F2 - Acquisition Parameters
Date_ 20220206
Time 23.06
INSTRUM av600
PROBHD 5 mm CPBBO BB-
PULPROG zg30
TD 98074
SOLVENT D2O
NS 512
DS 2
SWH 6487.889 Hz
FIDRES 0.066153 Hz
AQ 7.5582361 sec
RG 10
DW 77.067 usec
DE 13.04 usec
TE 298.0 K
D1 0.10000000 sec
TD0 1

======== CHANNEL f1 ========
SFO1 600.1327096 MHz
NUC1 1H
P1 10.06 usec
PLW1 30.00000000 W

F2 - Processing parameters
SI 65536
SF 600.1300183 MHz
WDW EM
SSB 0
LB 0.30 Hz
GB 0
PC 1.00

^1H NMR of 4.0 mM peptide **1b** + 4.0 mM peptide *ent*-**1b** in D_2O at 600 MHz and 298 K

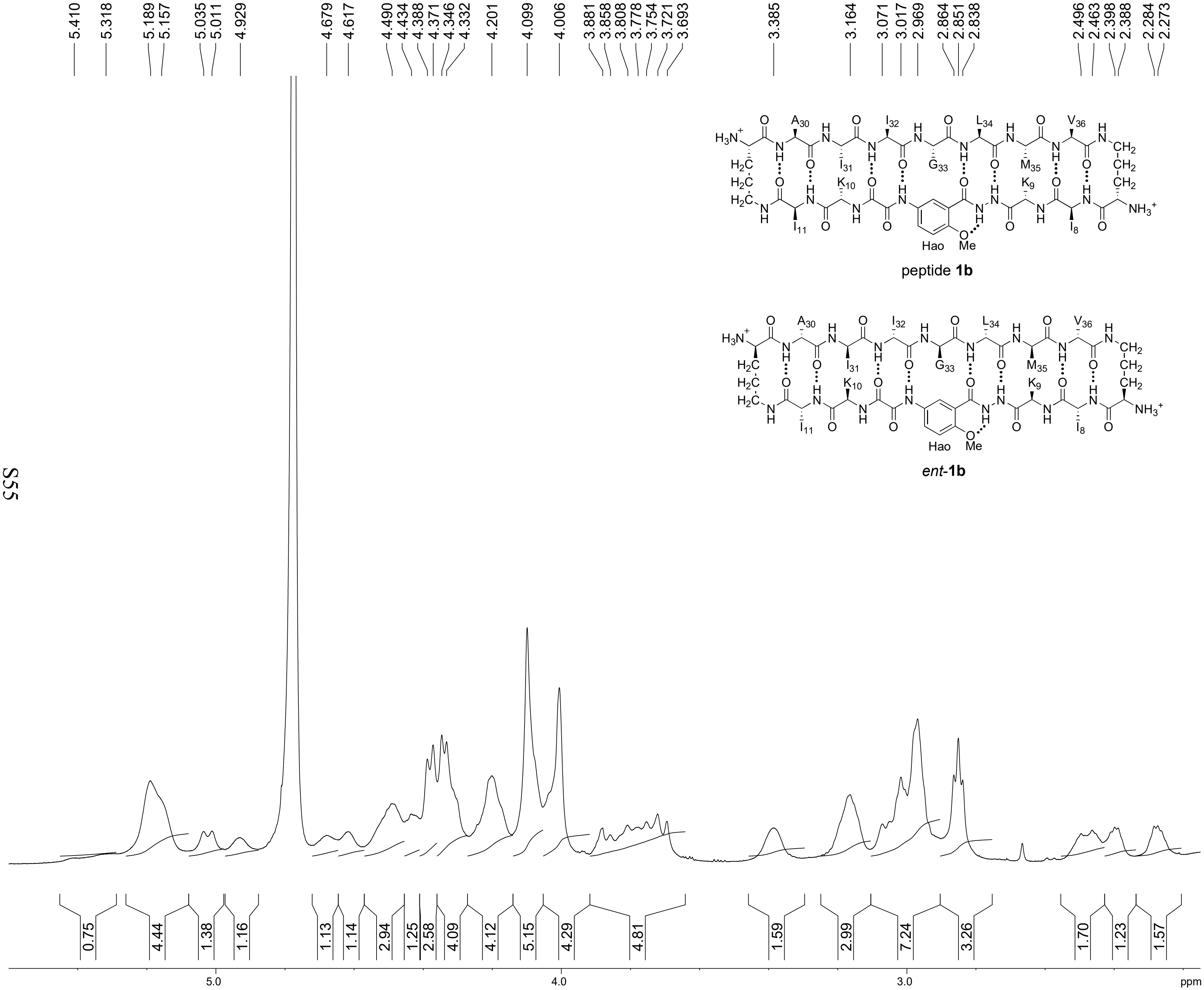

F2 - Acquisition Parameters
Date_ 20220206
Time 23.06
INSTRUM av600
PROBHD 5 mm CPBBO BB-
PULPROG zg30
TD 98074
SOLVENT D2O
NS 512
DS 2
SWH 6487.889 Hz
FIDRES 0.066153 Hz
AQ 7.5582361 sec
RG 10
DW 77.067 usec
DE 13.04 usec
TE 298.0 K
D1 0.10000000 sec
TD0 1

======== CHANNEL f1 ========
SFO1 600.1327096 MHz
NUC1 1H
P1 10.06 usec
PLW1 30.00000000 W

F2 - Processing parameters
SI 65536
SF 600.1300183 MHz
WDW EM
SSB 0
LB 0.30 Hz
GB 0
PC 1.00

^{1}H NMR of 4.0 mM peptide **1b** + 4.0 mM peptide *ent*-**1b** in D_2O at 600 MHz and 298 K

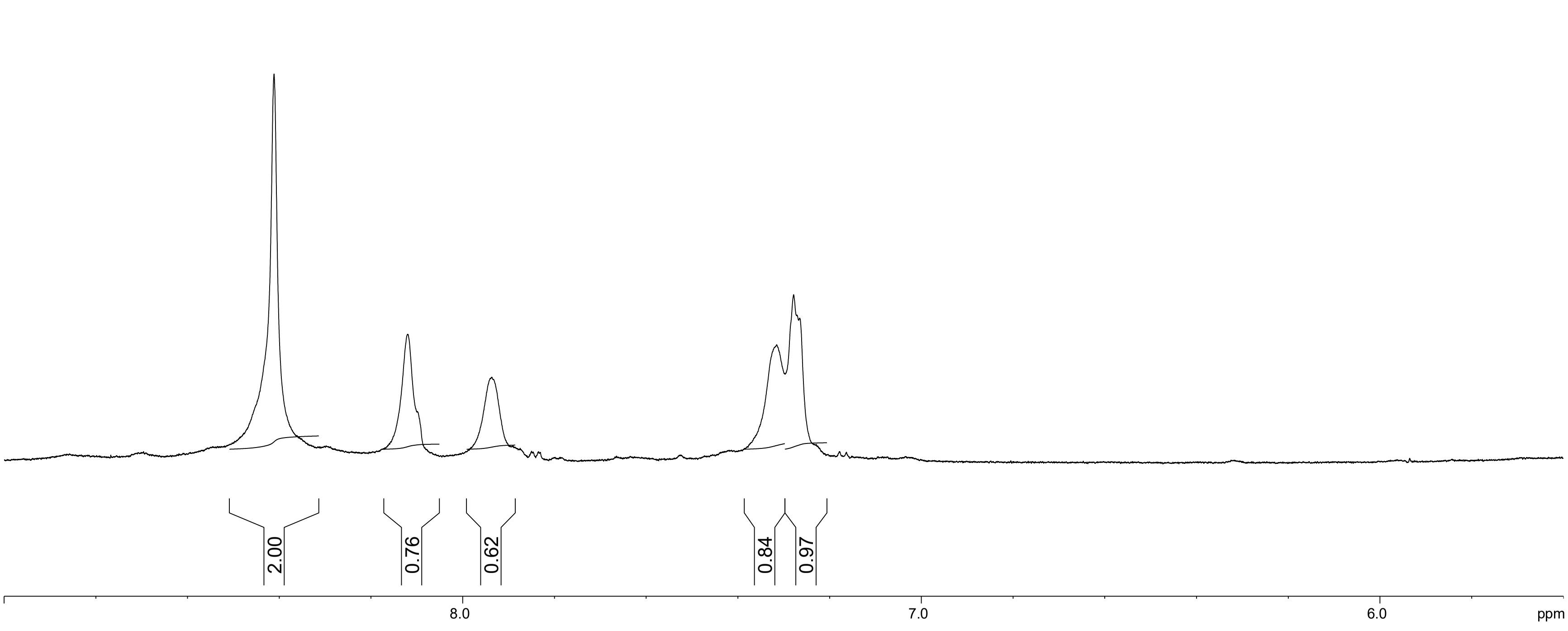

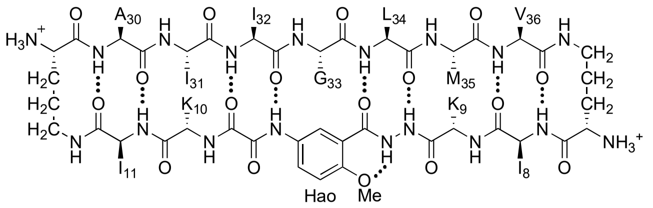

peptide **1b**

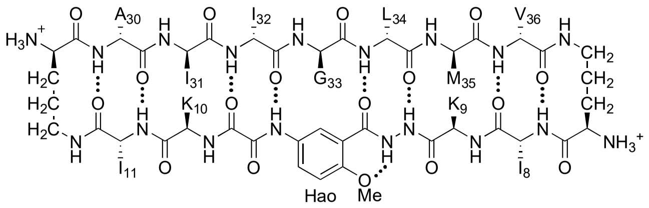

ent-**1b**

F2 - Acquisition Parameters
Date_ 20220206
Time 23.06
INSTRUM av600
PROBHD 5 mm CPBBO BB-
PULPROG zg30
TD 98074
SOLVENT D2O
NS 512
DS 2
SWH 6487.889 Hz
FIDRES 0.066153 Hz
AQ 7.5582361 sec
RG 10
DW 77.067 usec
DE 13.04 usec
TE 298.0 K
D1 0.10000000 sec
TD0 1

======== CHANNEL f1 ========
SFO1 600.1327096 MHz
NUC1 1H
P1 10.06 usec
PLW1 30.00000000 W

F2 - Processing parameters
SI 65536
SF 600.1300183 MHz
WDW EM
SSB 0
LB 0.30 Hz
GB 0
PC 1.00

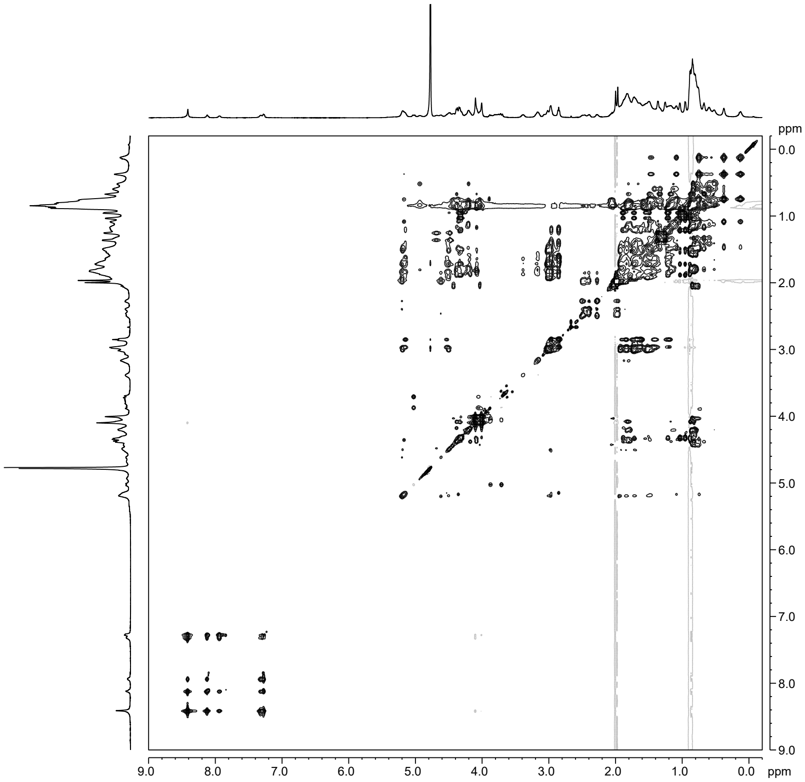

4.0 mM peptide **1b** + 4.0 mM *ent*-**1b**
in D_2O, 150-ms mixing time, 298 K
600 MHz TOCSY spectrum
presaturation suppression of the HOD peak

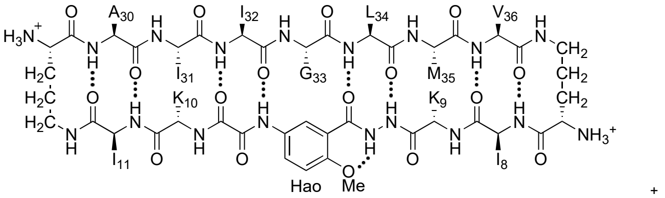

peptide **1b**

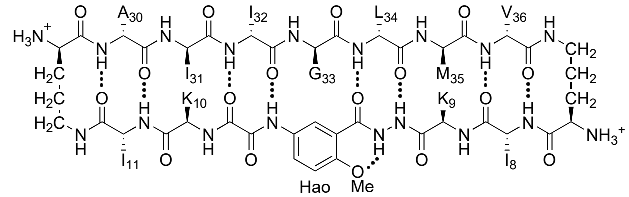

ent-**1b**

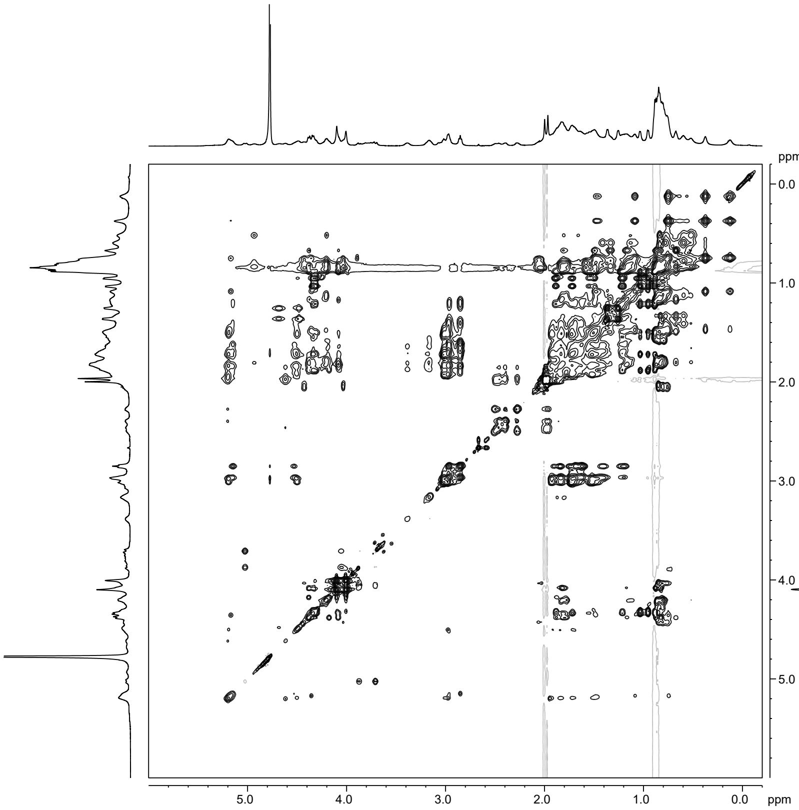

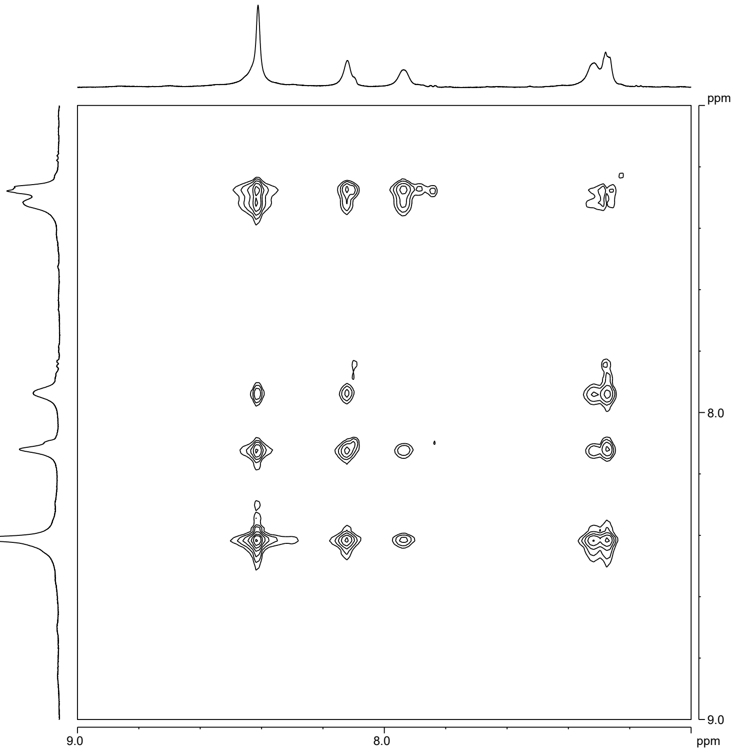

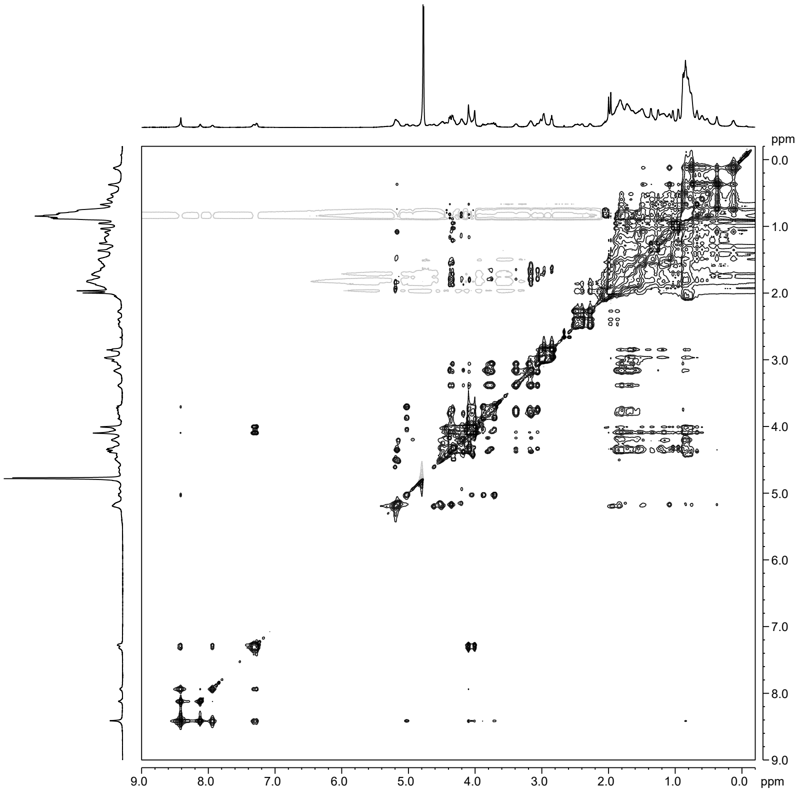

4.0 mM peptide **1b** + 4.0 mM *ent*-**1b**
in D_2O, 200-ms mixing time, 298 K
600 MHz NOESY spectrum
presaturation suppression of the HOD peak

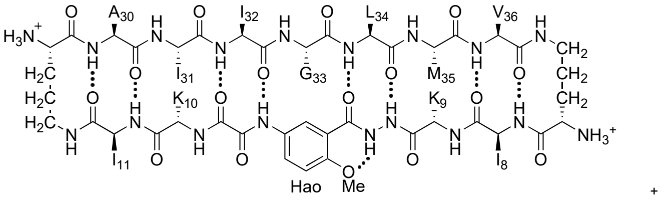

peptide **1b**

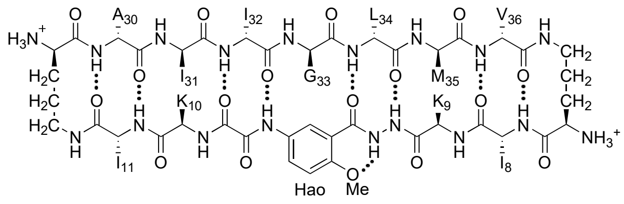

ent-**1b**

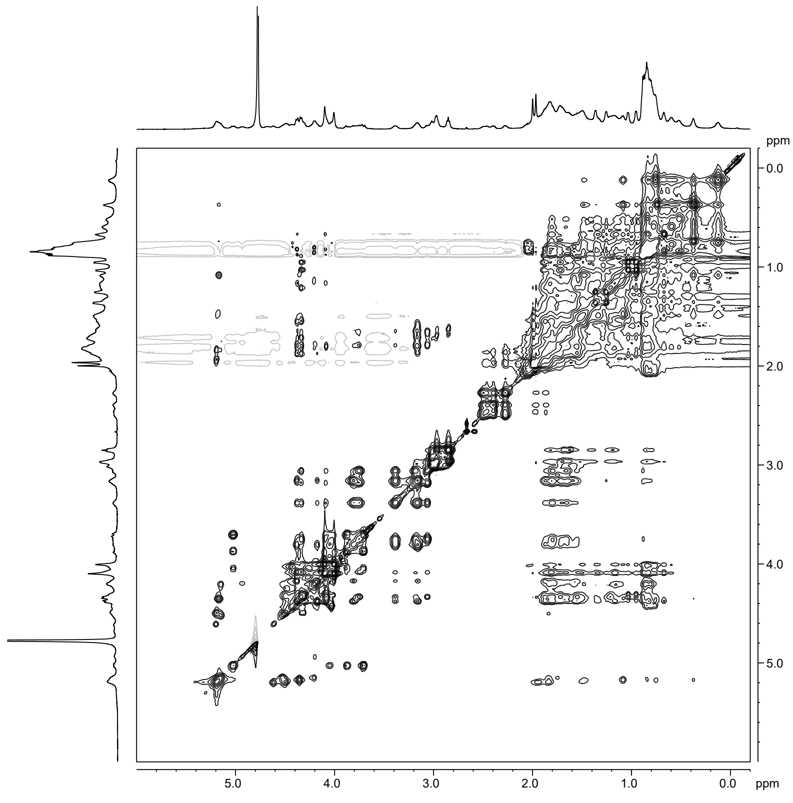

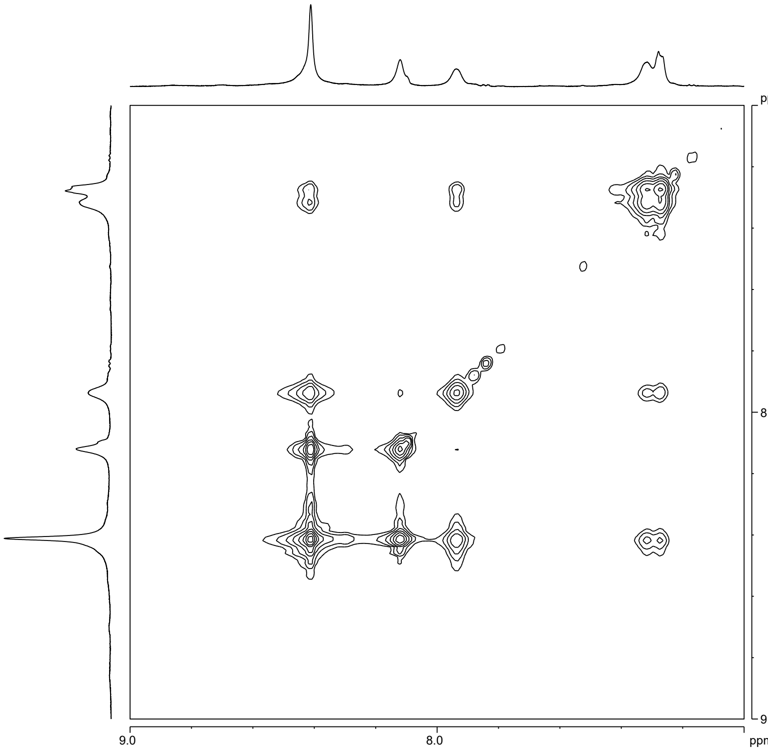

Characterization of peptide ***1a***

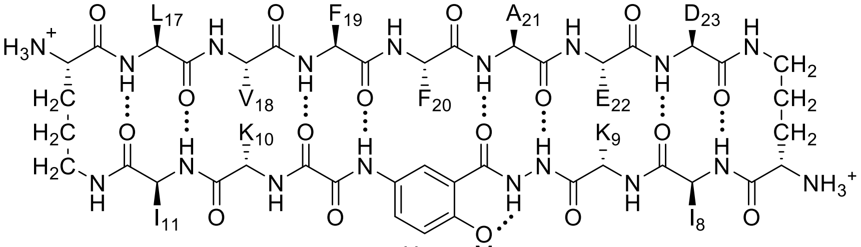

peptide **1a**

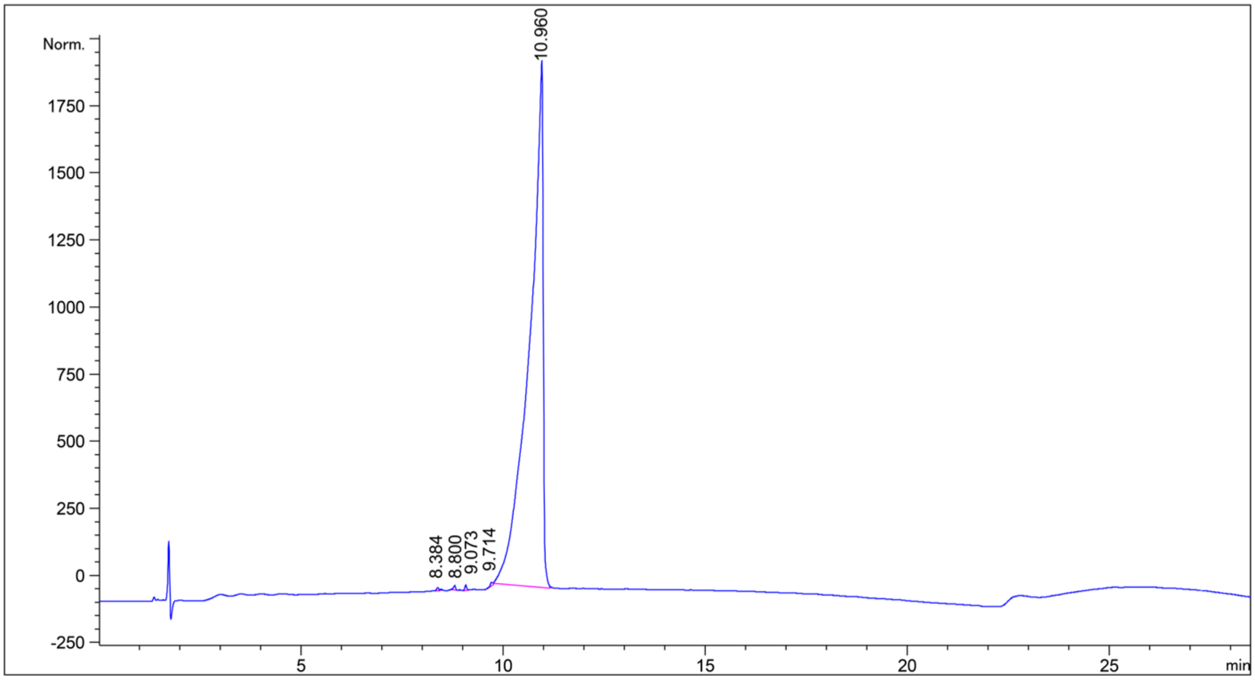

Signal 1: MWD1 A, Sig=214,4 Ref=off

Peak #	RetTime [min]	Type	Width [min]	Area [mAU*s]	Height [mAU]	Area %
1	8.384	MM	0.0569	40.10479	11.75607	0.0964
2	8.800	MM	0.0592	49.23368	13.86946	0.1183
3	9.073	MM	0.0552	59.23932	17.88090	0.1423
4	9.714	MM	0.0729	53.46276	12.22763	0.1285
5	10.960	MM	0.3892	4.14158e4	1773.62488	99.5145

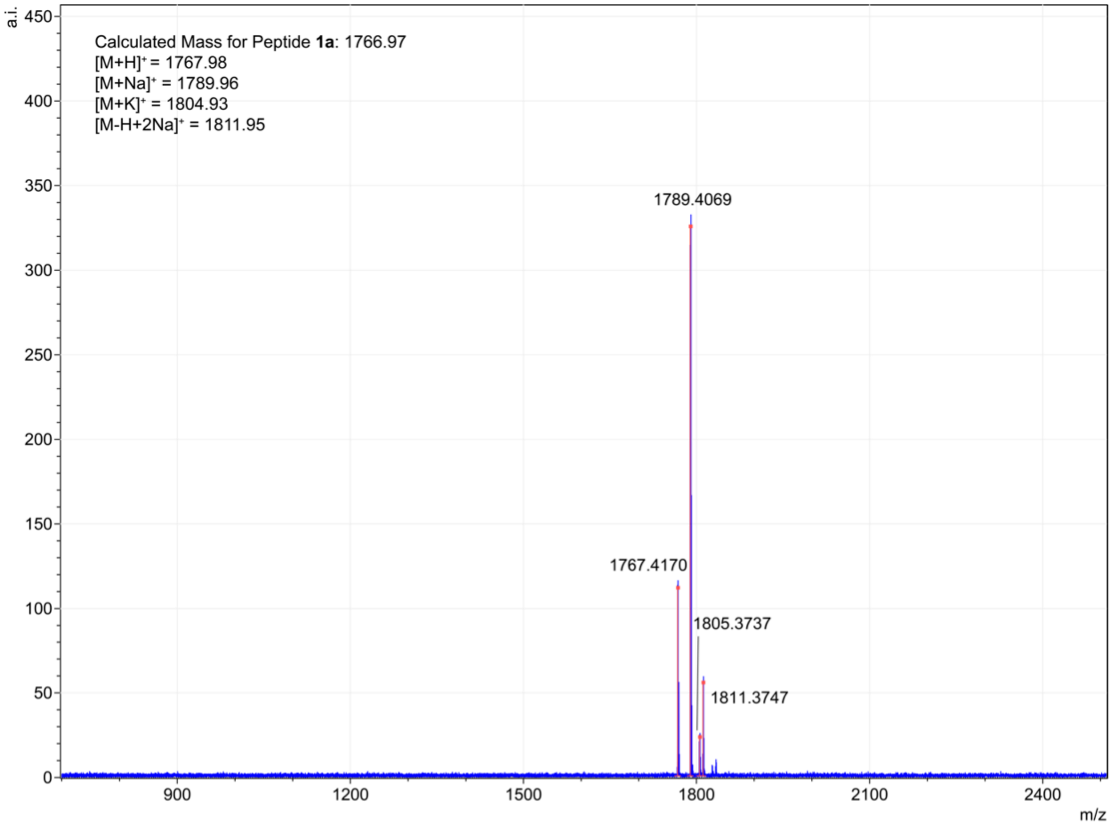

Calculated Mass for Peptide **1a**: 1766.97
[M+H]+ = 1767.98
[M+Na]+ = 1789.96
[M+K]+ = 1804.93
[M-H+2Na]+ = 1811.95
1789.4069
1767.4170
1805.3737
1811.3747
a.i.
m/z

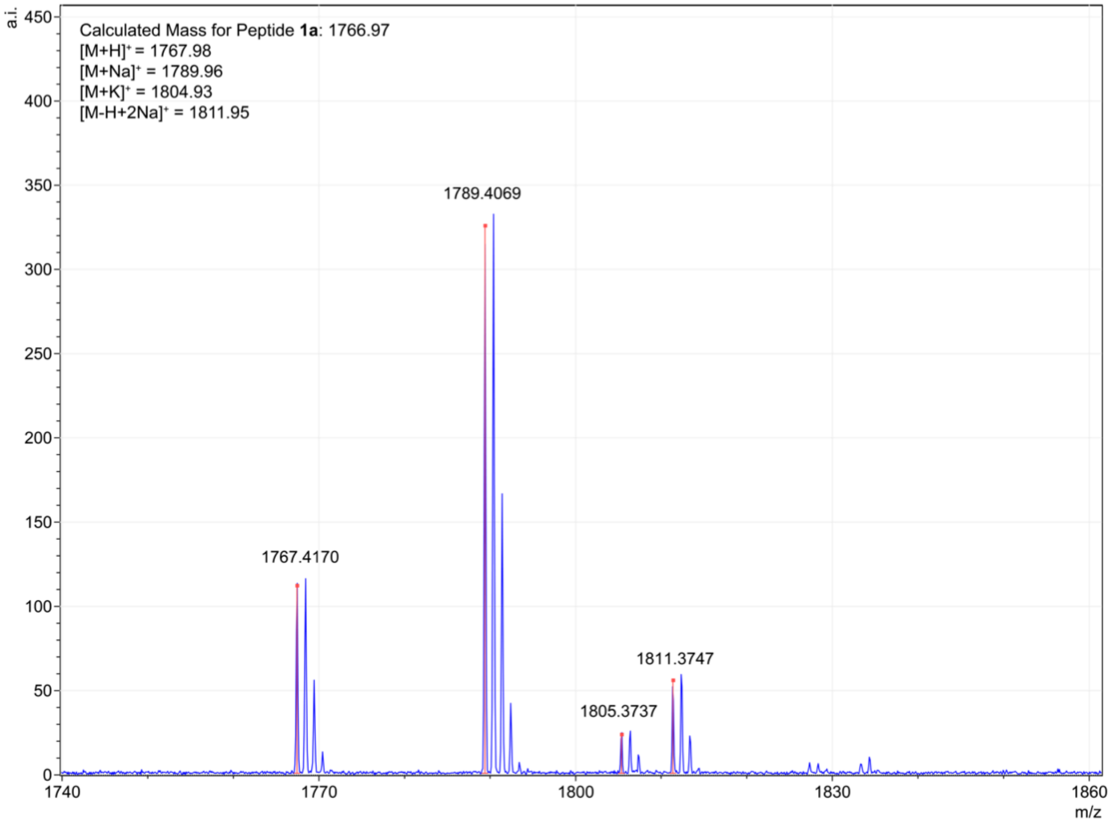

Calculated Mass for Peptide **1a**: 1766.97
[M+H]+ = 1767.98
[M+Na]+ = 1789.96
[M+K]+ = 1804.93
[M-H+2Na]+ = 1811.95
1789.4069
1767.4170
1811.3747
1805.3737
a.i.
m/z

Characterization of peptide ent-***1a***

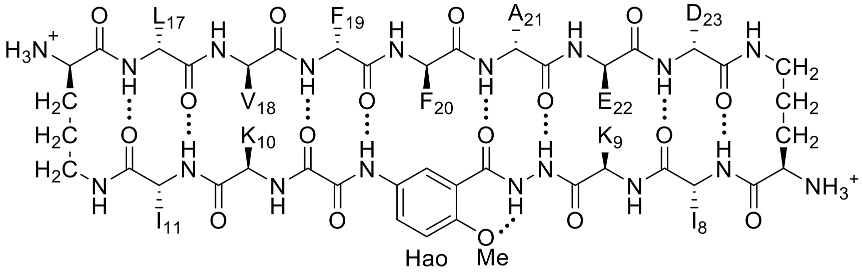

ent-**1a**

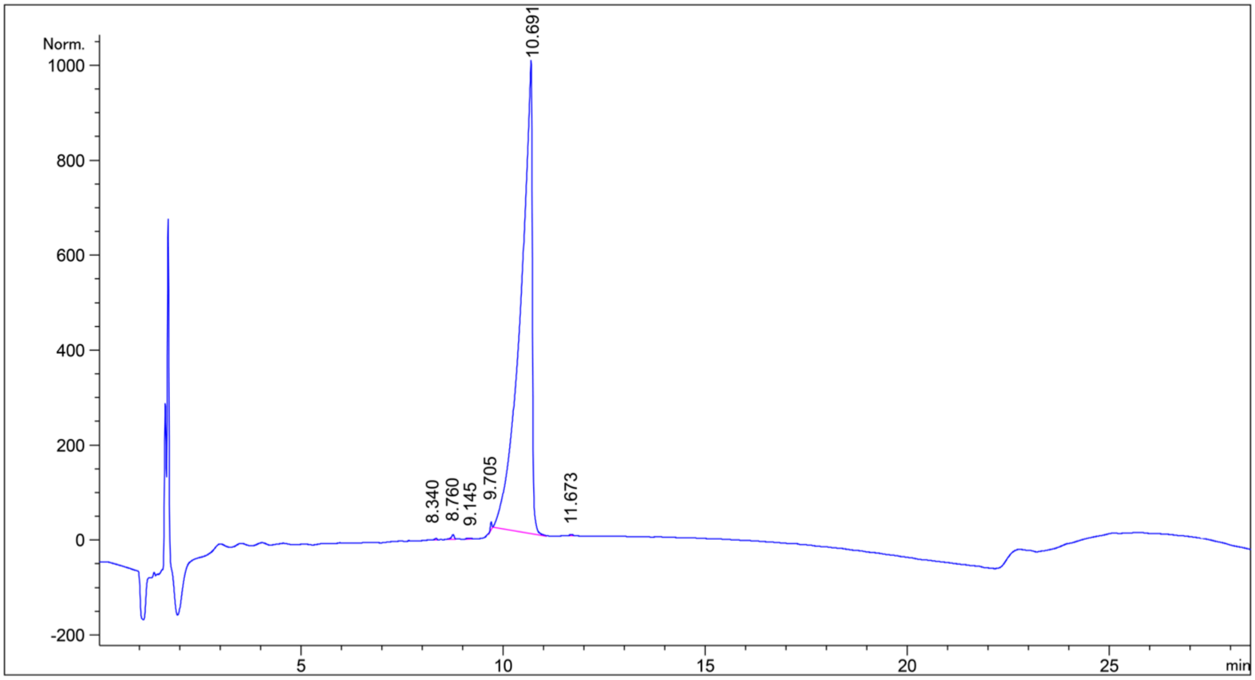

Signal 1: MWD1 A, Sig=214,4 Ref=off

Peak #	RetTime [min]	Type	Width [min]	Area [mAU*s]	Height [mAU]	Area %
1	8.340	MM	0.0577	11.30358	3.26538	0.0616
2	8.760	MM	0.0800	41.89858	8.72646	0.2282
3	9.145	MM	0.1498	17.51583	1.94829	0.0954
4	9.705	MM	0.0430	39.48652	15.31090	0.2150
5	10.691	MM	0.3378	1.82403e4	899.93854	99.3321
6	11.673	MM	0.0801	12.43770	2.58835	0.0677

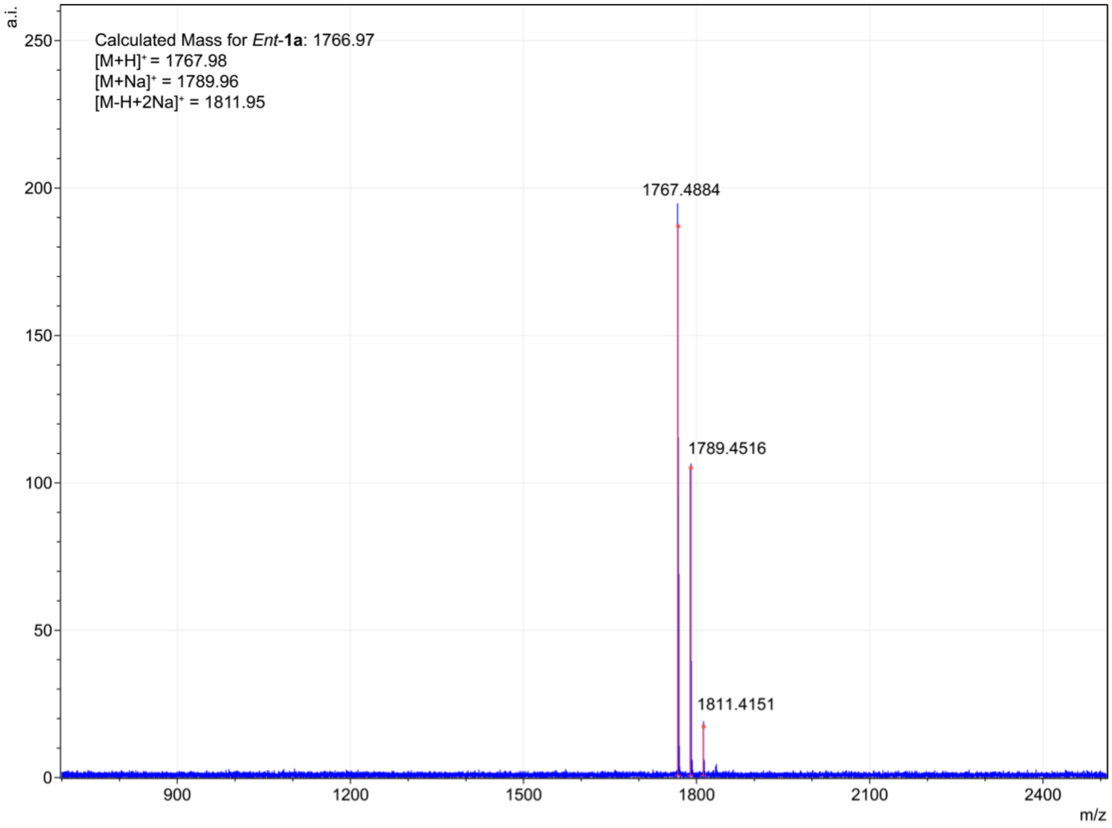
a.i.
Calculated Mass for *Ent*-**1a**: 1766.97
[M+H]+ = 1767.98
[M+Na]+ = 1789.96
[M-H+2Na]+ = 1811.95
1767.4884
1789.4516
1811.4151
250
200
150
100
50
0
900
1200
1500
1800
2100
2400
m/z

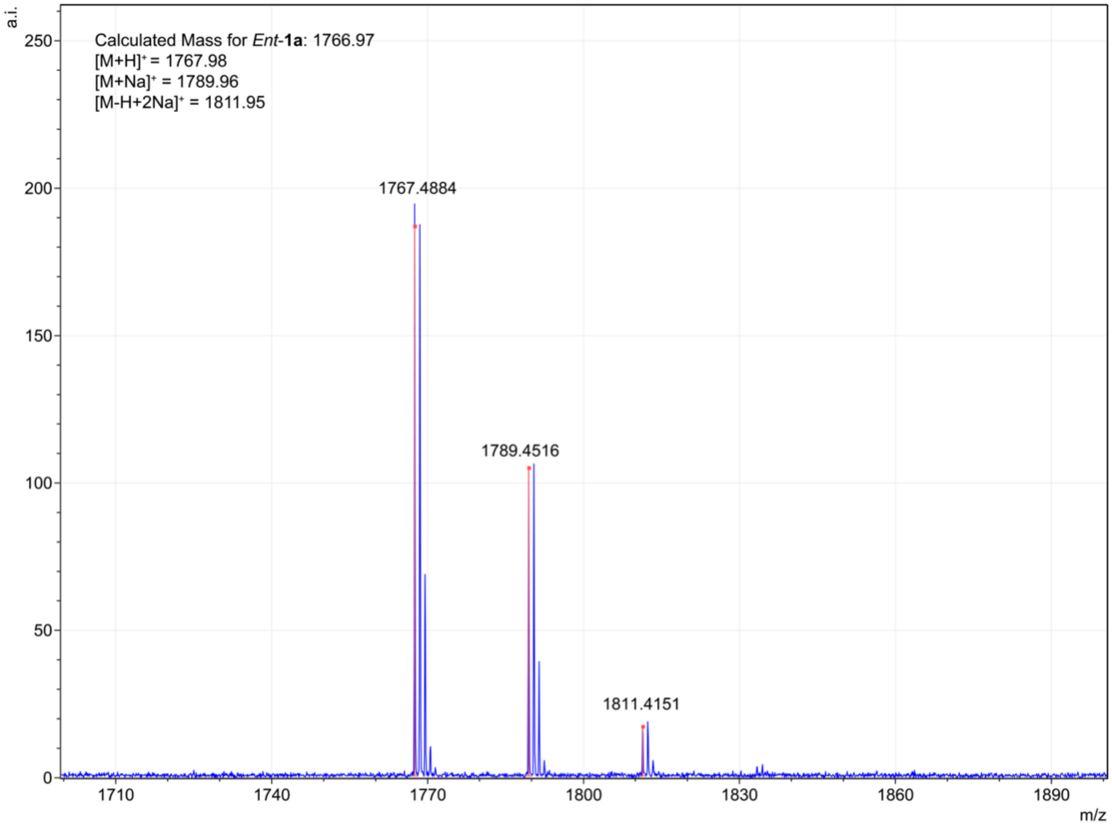
a.i.
Calculated Mass for *Ent*-**1a**: 1766.97
[M+H]+ = 1767.98
[M+Na]+ = 1789.96
[M-H+2Na]+ = 1811.95
1767.4884
1789.4516
1811.4151
250
200
150
100
50
0
1710
1740
1770
1800
1830
1860
1890
m/z

*Characterization of peptide **2a***

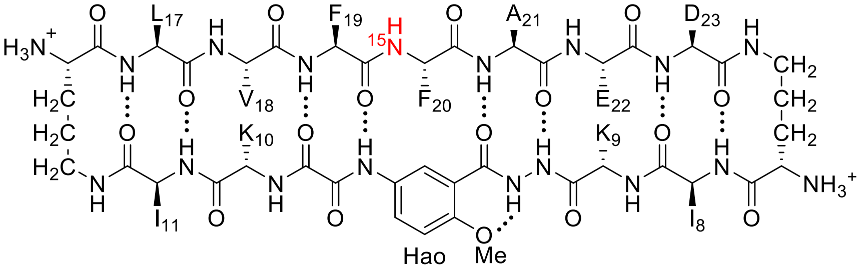

peptide **2a**

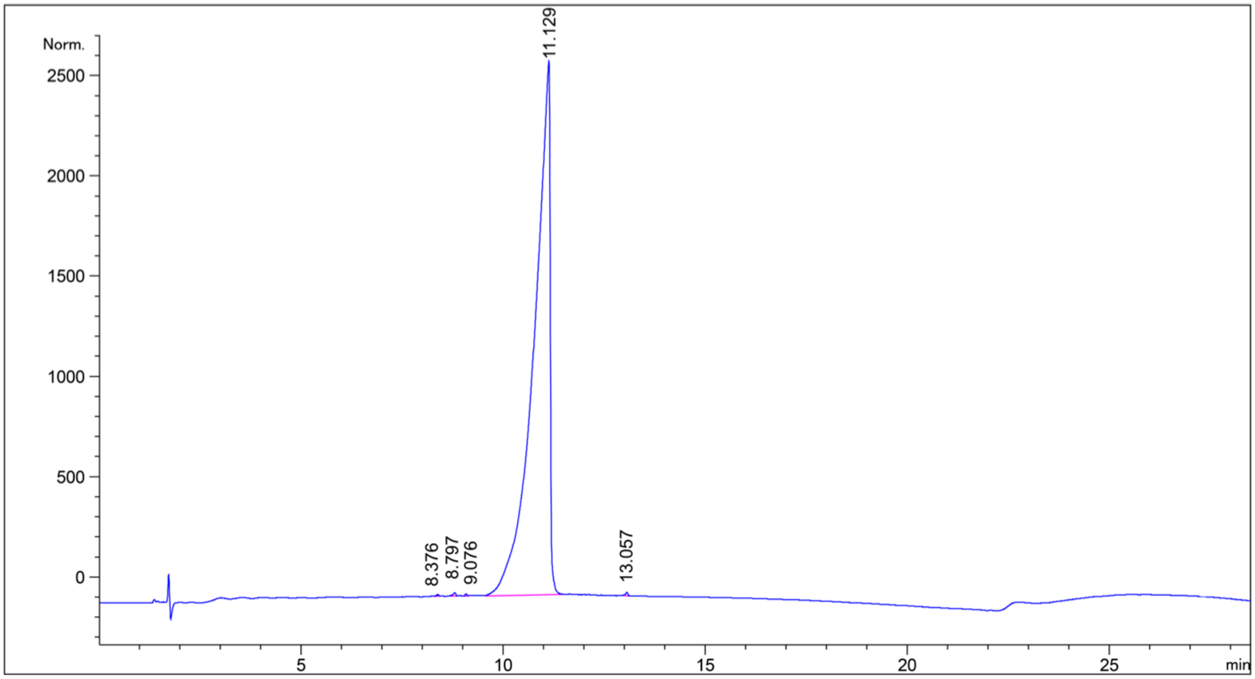

Signal 1: MWD1 A, Sig=214,4 Ref=off

Peak #	RetTime [min]	Type	Width [min]	Area [mAU*s]	Height [mAU]	Area %
1	8.376	MM	0.0707	34.15193	8.05459	0.0514
2	8.797	MM	0.0726	60.69348	13.94270	0.0914
3	9.076	MM	0.0597	32.12604	8.97329	0.0484
4	11.129	MM	0.4589	6.62052e4	2404.37671	99.7111
5	13.057	MM	0.0704	64.87210	15.35158	0.0977

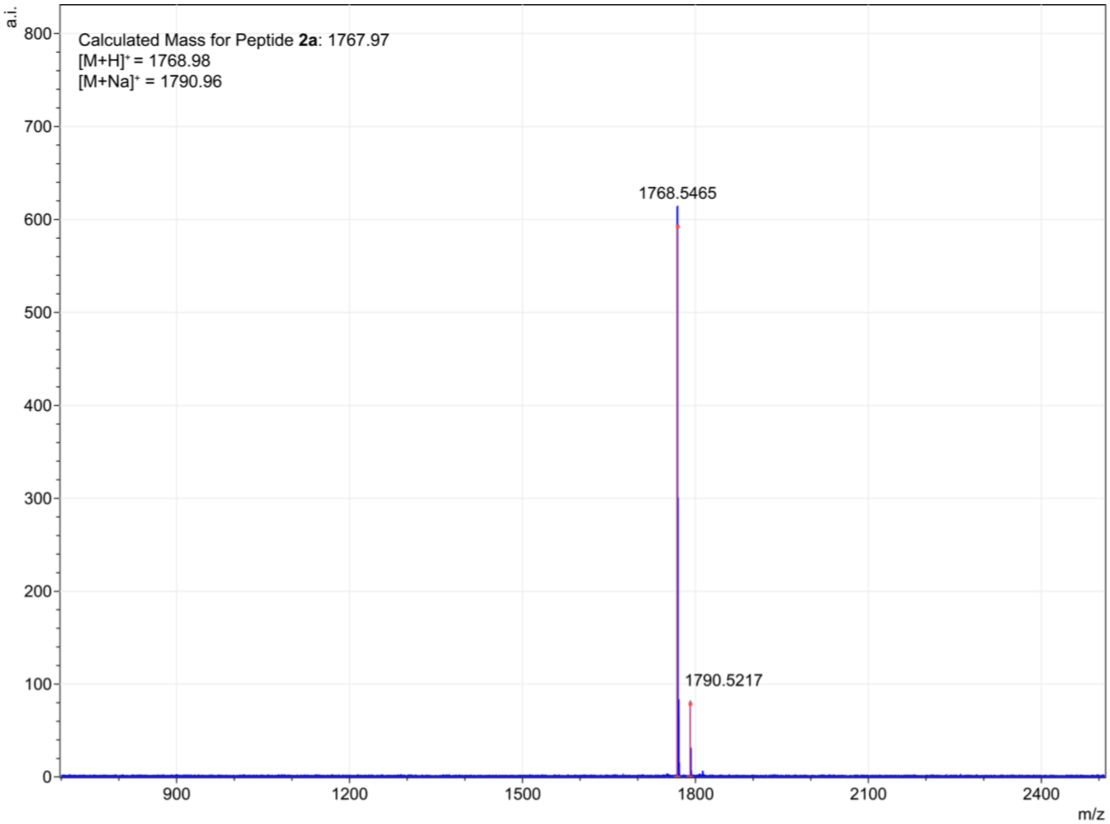
a.i.
Calculated Mass for Peptide **2a**: 1767.97
[M+H]+ = 1768.98
[M+Na]+ = 1790.96
1768.5465
1790.5217
800
700
600
500
400
300
200
100
0
900
1200
1500
1800
2100
2400
m/z

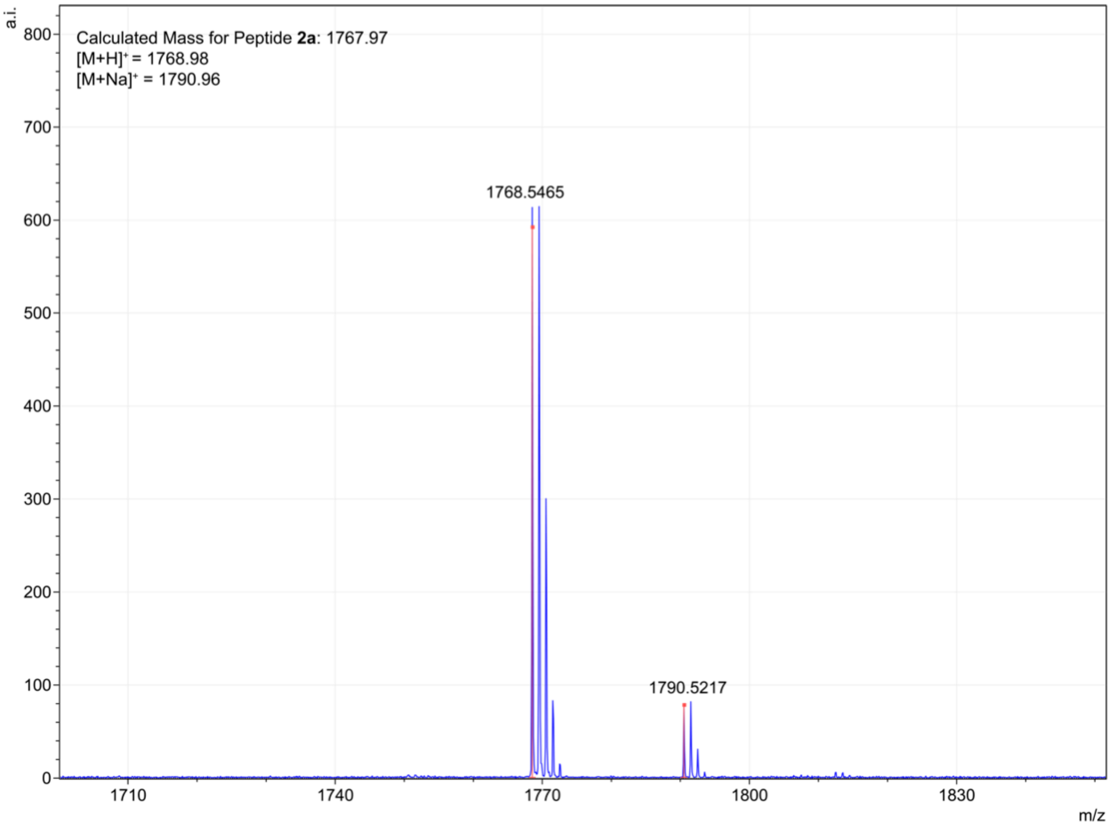
a.i.
Calculated Mass for Peptide **2a**: 1767.97
[M+H]+ = 1768.98
[M+Na]+ = 1790.96
1768.5465
1790.5217
800
700
600
500
400
300
200
100
0
1710
1740
1770
1800
1830
m/z

*Characterization of peptide **3a***

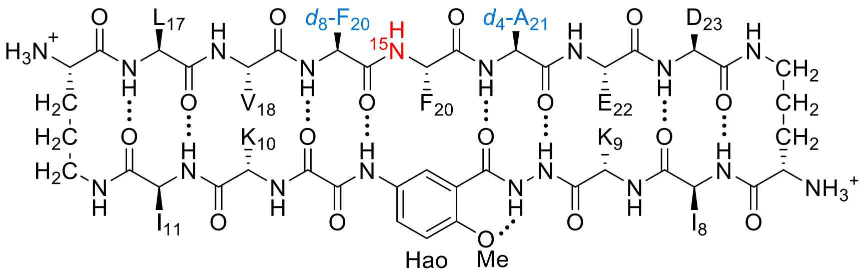

peptide **3a**

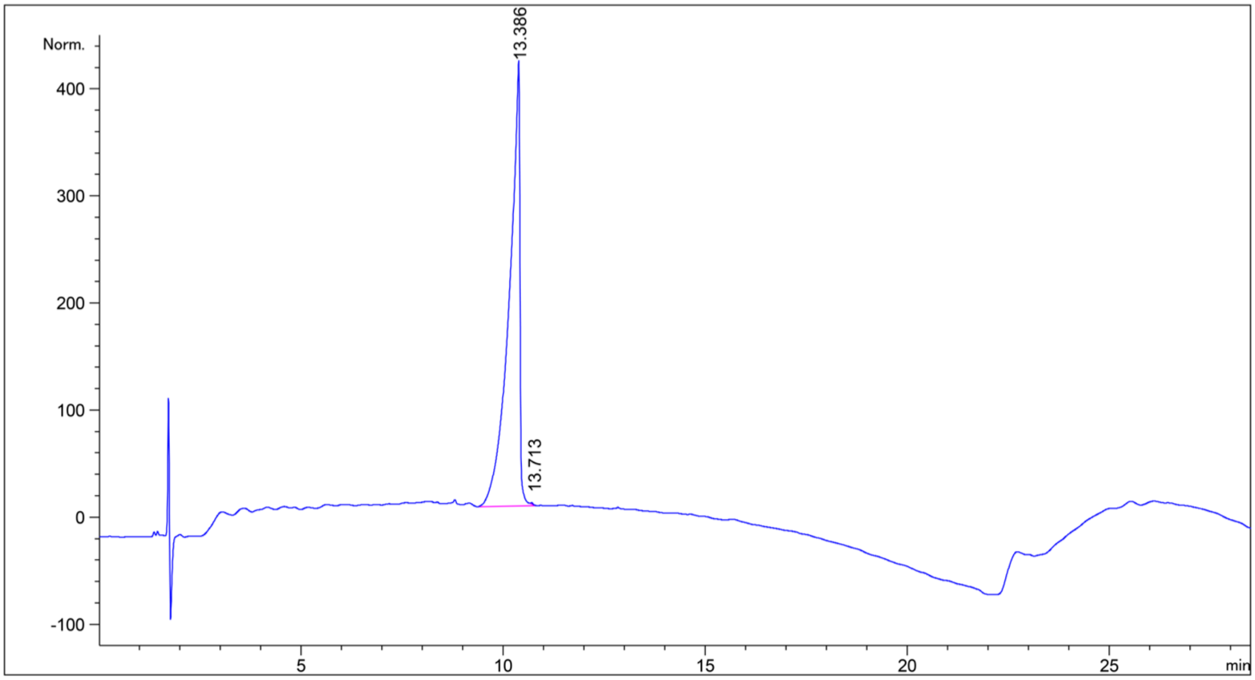

Signal 1: MWD1 A, Sig=214,4 Ref=off

Peak #	RetTime [min]	Type	Width [min]	Area [mAU*s]	Height [mAU]	Area %
1	10.386	MM R	0.3037	6846.85889	375.73801	99.9235
2	10.713	MM T	0.0610	5.23864	1.43016	0.0765

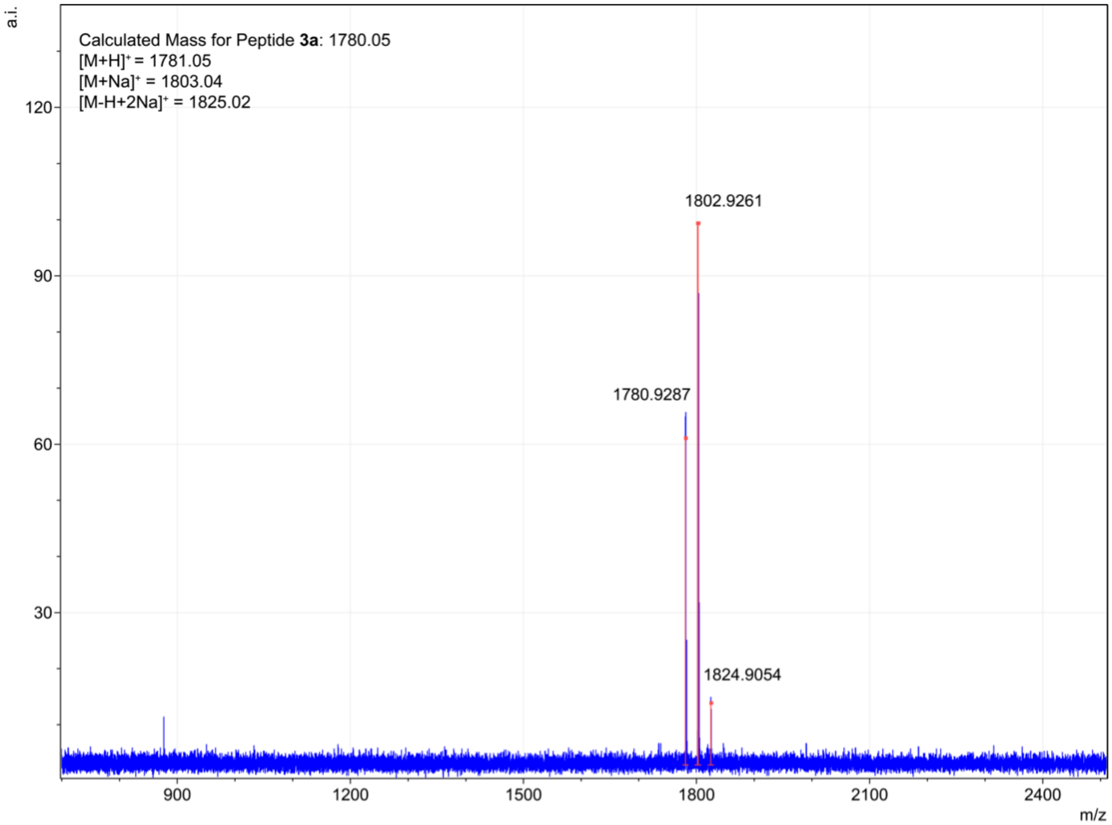
a.i.
Calculated Mass for Peptide **3a**: 1780.05
[M+H]+ = 1781.05
[M+Na]+ = 1803.04
[M-H+2Na]+ = 1825.02
1802.9261
1780.9287
1824.9054
120
90
60
30
900
1200
1500
1800
2100
2400
m/z

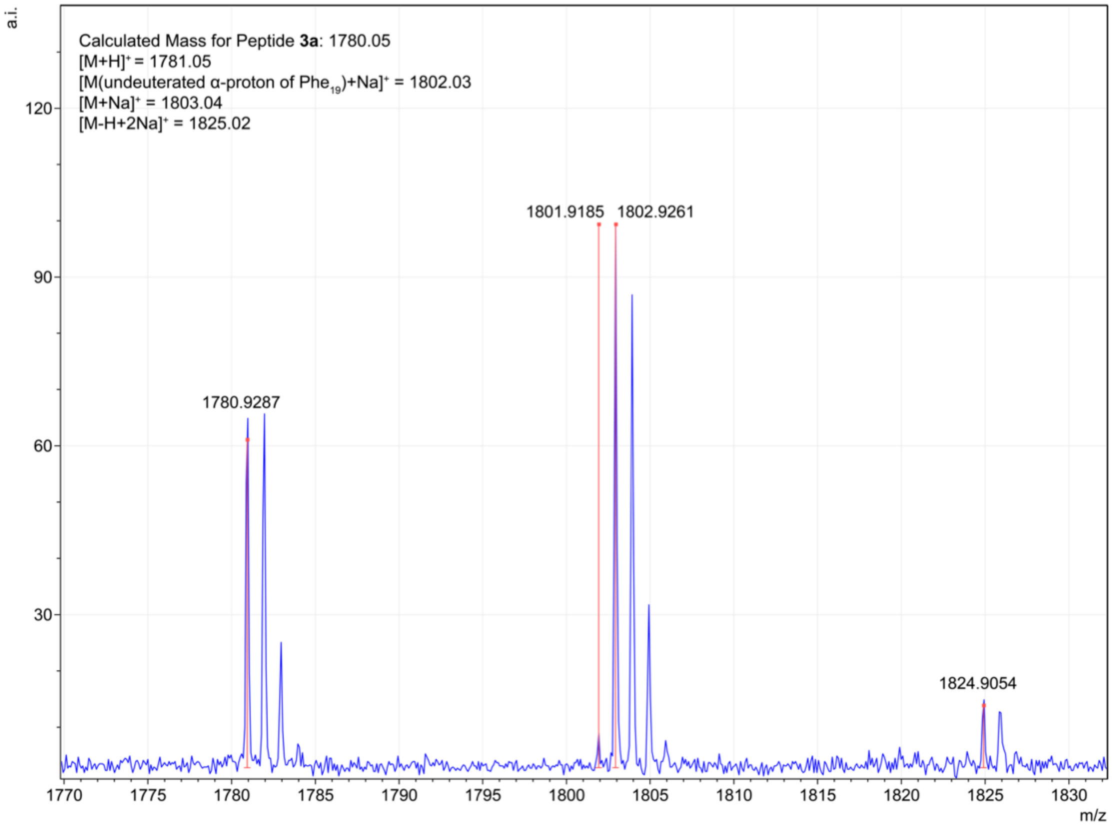
a.i.
Calculated Mass for Peptide **3a**: 1780.05
[M+H]+ = 1781.05
[M(undeuterated α-proton of Phe19)+Na]+ = 1802.03
[M+Na]+ = 1803.04
[M-H+2Na]+ = 1825.02
1801.9185
1802.9261
1780.9287
1824.9054
120
90
60
30
1770
1775
1780
1785
1790
1795
1800
1805
1810
1815
1820
1825
1830
m/z

*Characterization of peptide **1b***

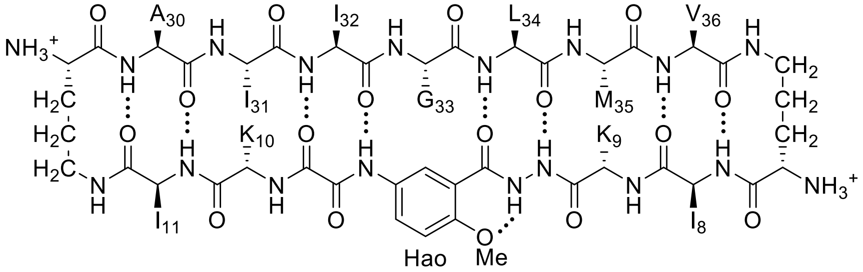

peptide **1b**

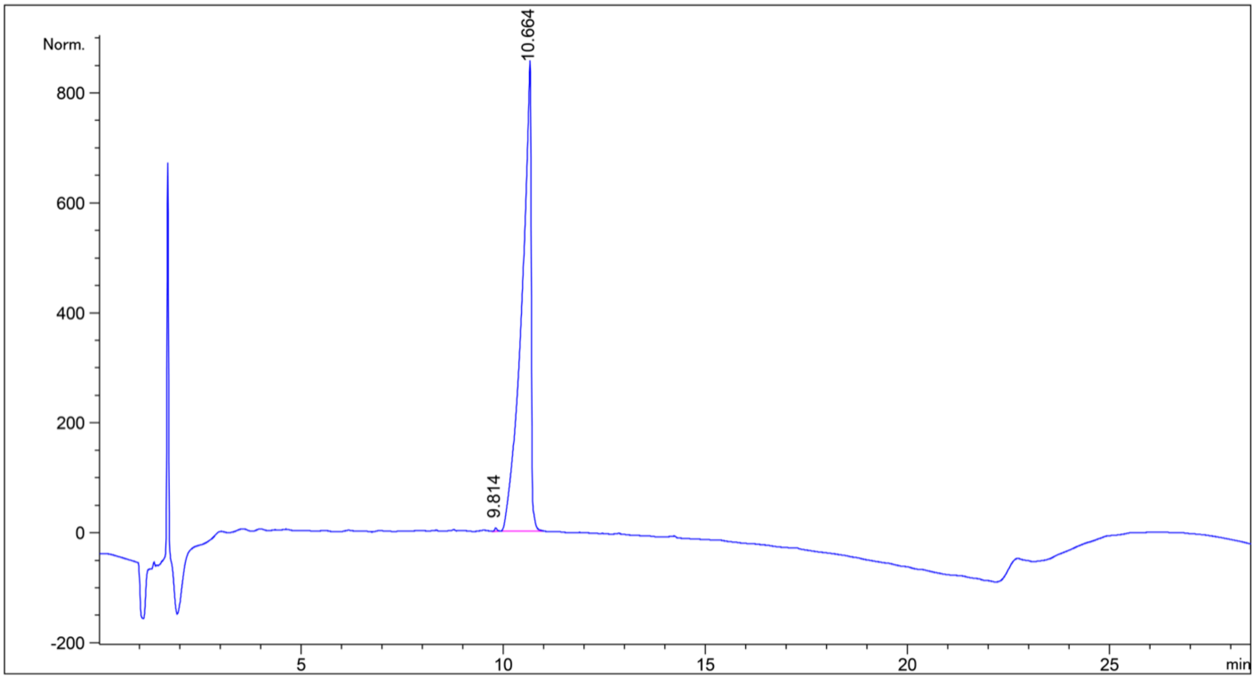

Signal 1: MWD1 A, Sig=214,4 Ref=off

Peak #	RetTime [min]	Type	Width [min]	Area [mAU*s]	Height [mAU]	Area %
1	9.814	MM	0.0699	23.07438	5.50104	0.1828
2	10.664	MM	0.2712	1.26018e4	774.44647	99.8172

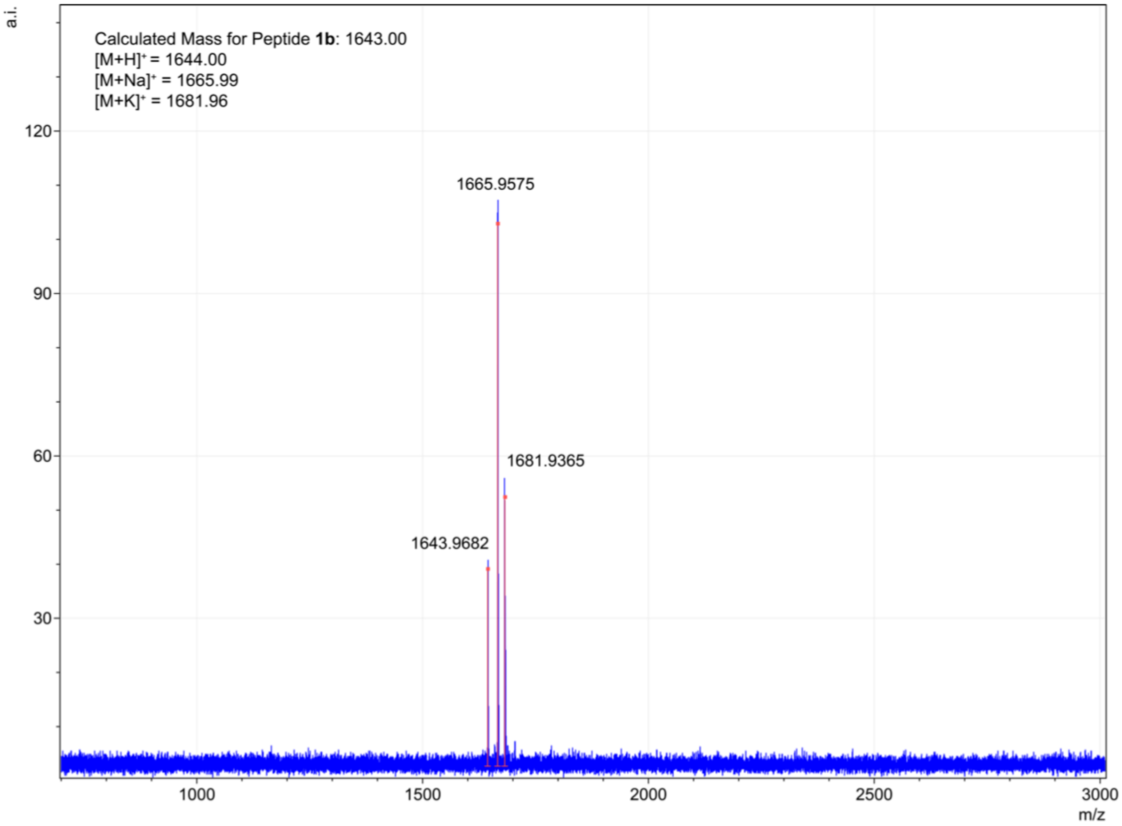

Calculated Mass for Peptide **1b**: 1643.00
[M+H]+ = 1644.00
[M+Na]+ = 1665.99
[M+K]+ = 1681.96
1665.9575
1681.9365
1643.9682
a.i.
m/z

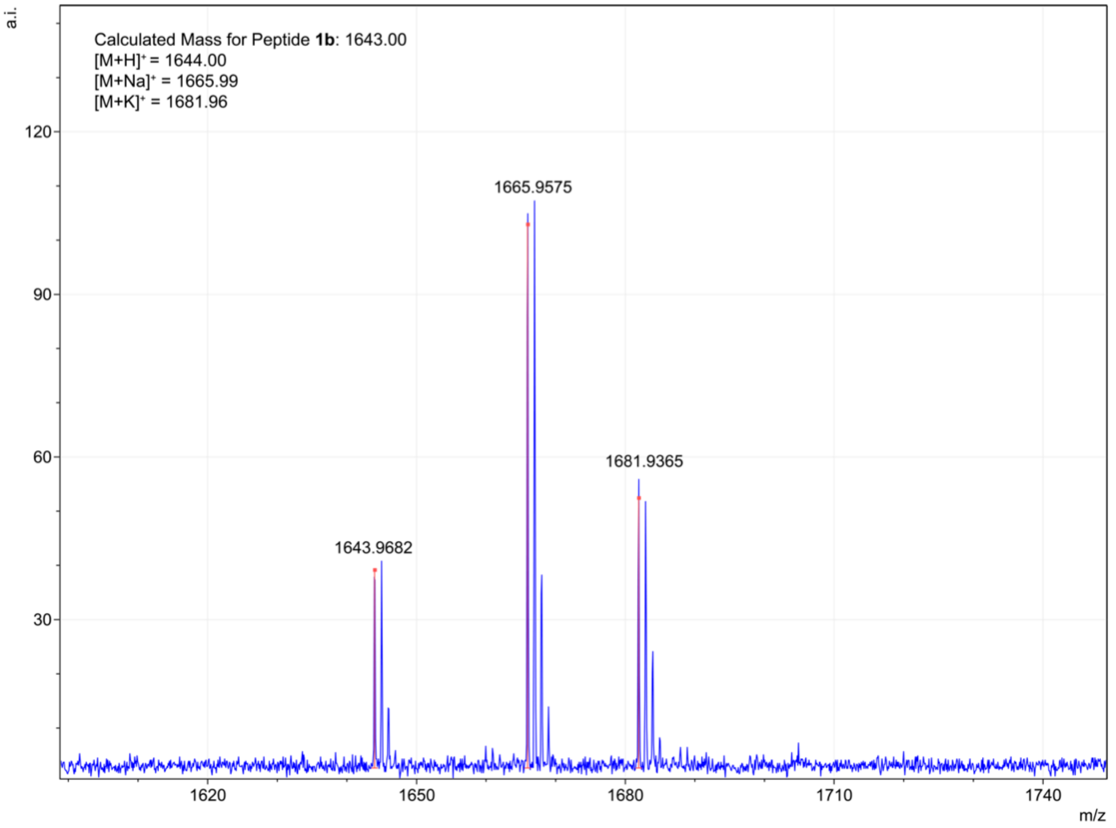

Calculated Mass for Peptide **1b**: 1643.00
[M+H]+ = 1644.00
[M+Na]+ = 1665.99
[M+K]+ = 1681.96
1665.9575
1681.9365
1643.9682
a.i.
m/z

Characterization of peptide ent-***1b***

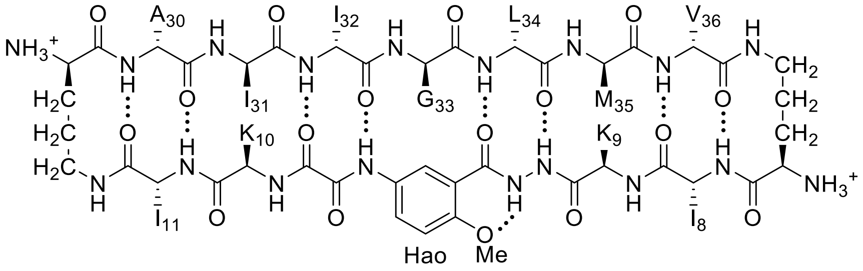

ent-**1b**

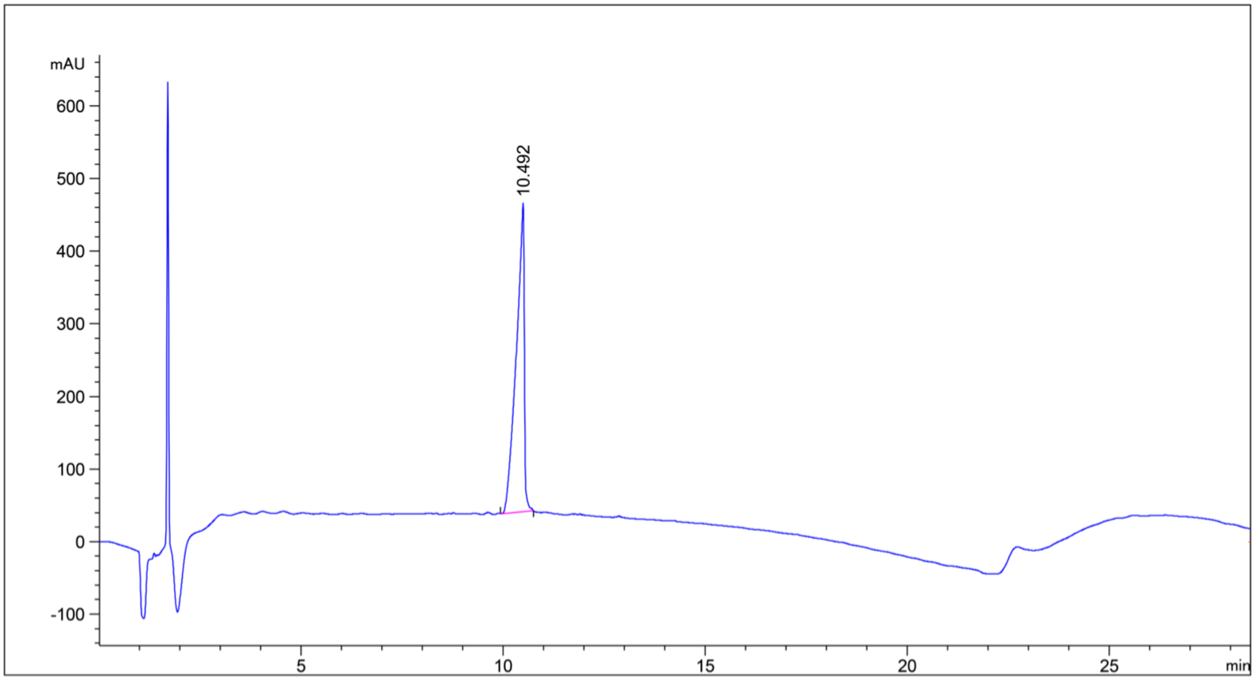

Signal 1: MWD1 A, Sig=214,4 Ref=off

Peak #	RetTime [min]	Type	Width [min]	Area [mAU*s]	Height [mAU]	Area %
1	10.492	MM	0.2310	5919.69141	427.04166	100.0000

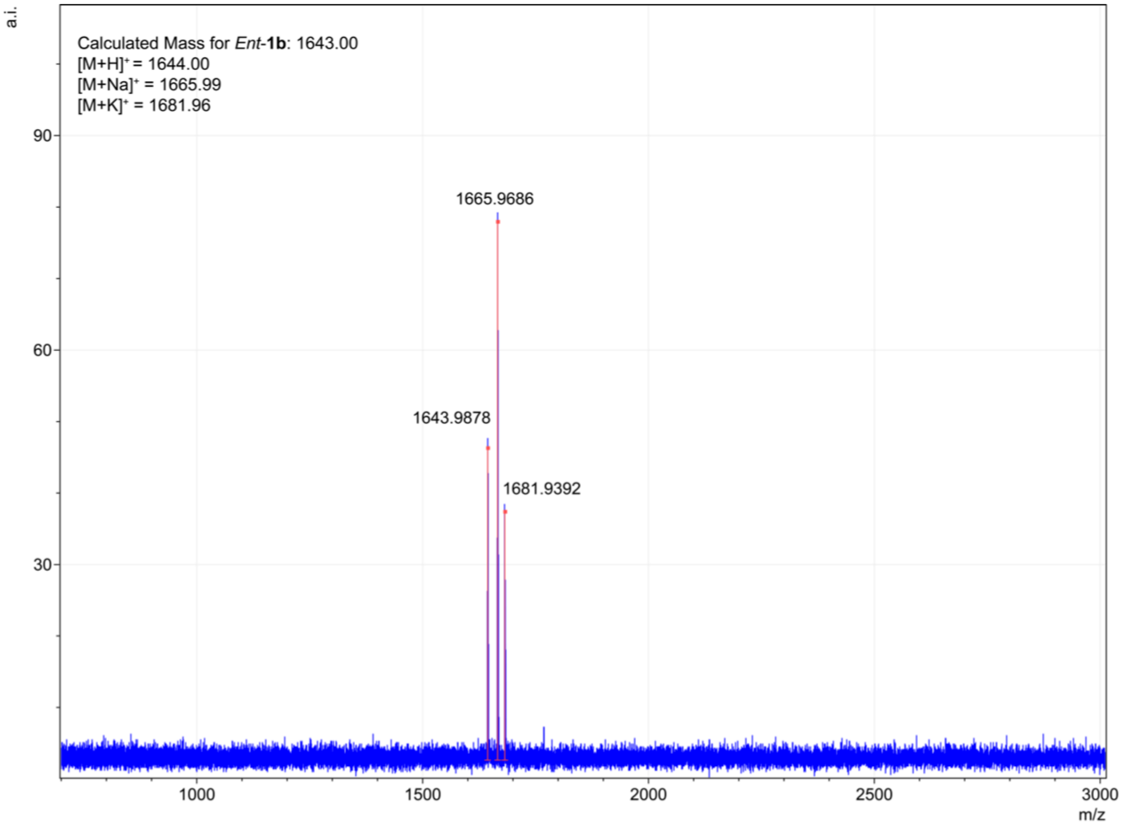
a.i.
Calculated Mass for *Ent*-**1b**: 1643.00
[M+H]+ = 1644.00
[M+Na]+ = 1665.99
[M+K]+ = 1681.96
90
60
30
1665.9686
1643.9878
1681.9392
1000
1500
2000
2500
3000
m/z

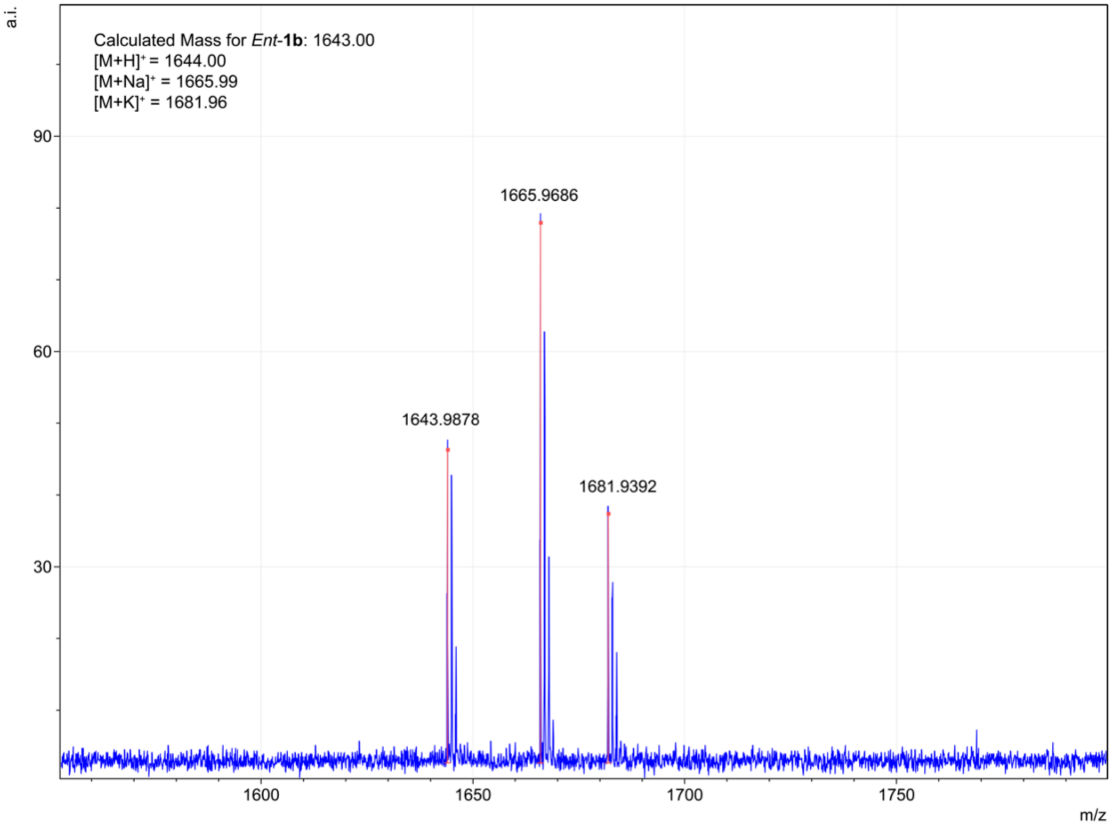
a.i.
Calculated Mass for *Ent*-**1b**: 1643.00
[M+H]+ = 1644.00
[M+Na]+ = 1665.99
[M+K]+ = 1681.96
90
60
30
1665.9686
1643.9878
1681.9392
1600
1650
1700
1750
m/z

*Characterization of peptide **2b***

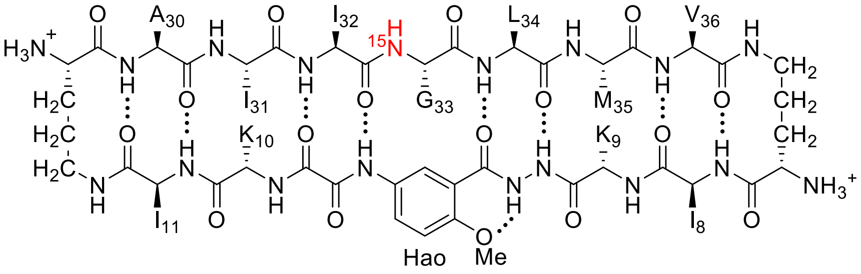

peptide **2b**

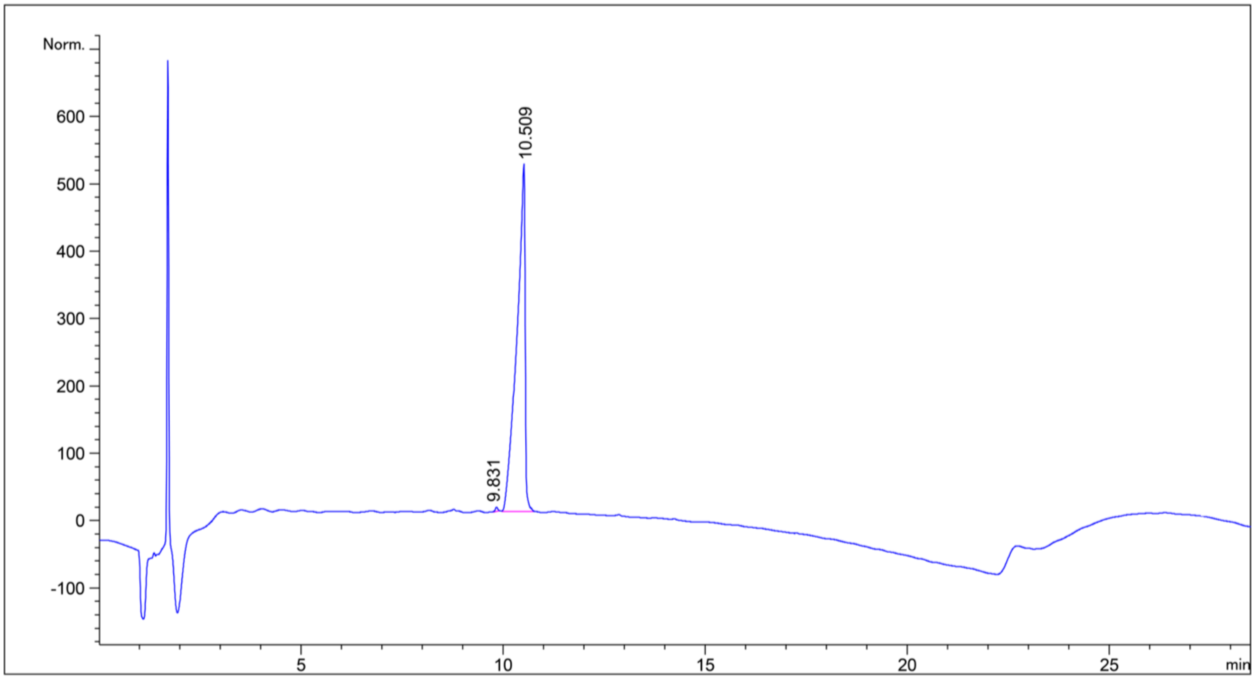


```
Signal 1: MWD1 A, Sig=214,4 Ref=off

Peak RetTime Type  Width     Area      Height     Area
  #   [min]        [min]   [mAU*s]     [mAU]        %
----|-------|----|-------|----------|----------|--------|
   1   9.831 MM    0.0704   25.07612    5.94035   0.3799
   2  10.509 MM    0.2349 6576.07031  466.65961  99.6201
```

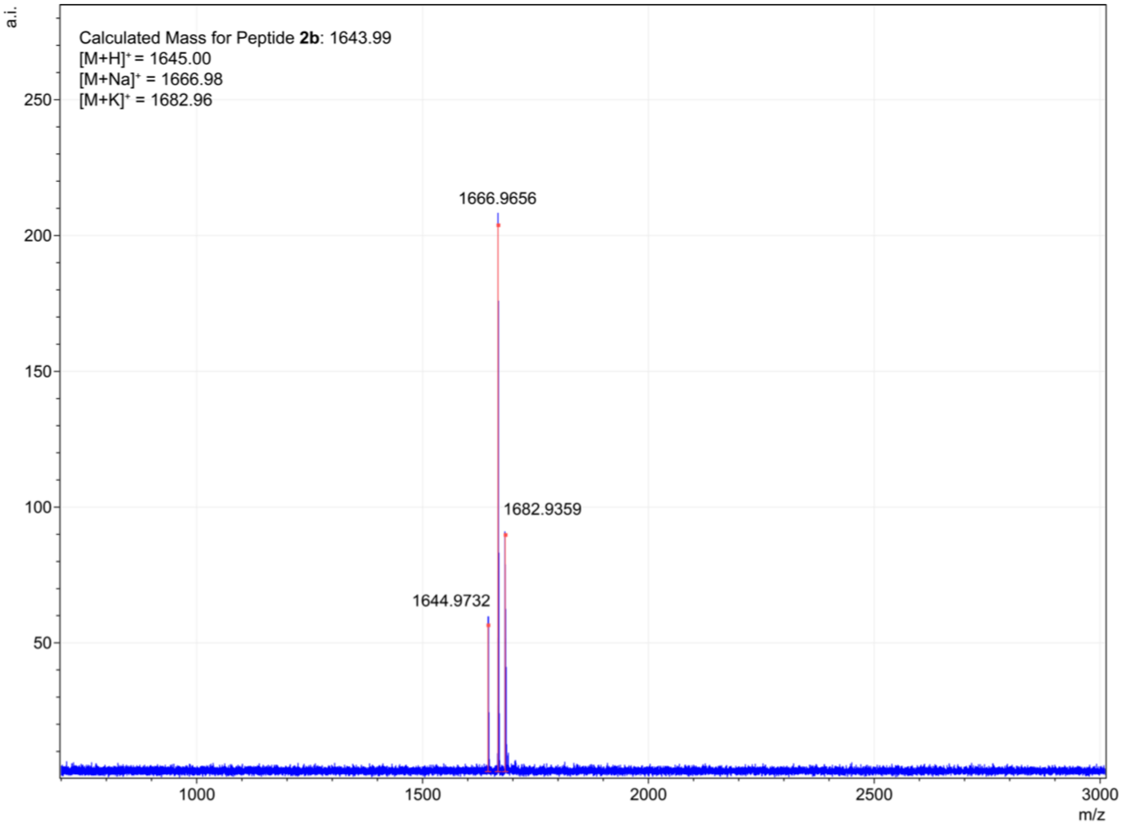

Calculated Mass for Peptide **2b**: 1643.99
[M+H]+ = 1645.00
[M+Na]+ = 1666.98
[M+K]+ = 1682.96
1666.9656
1682.9359
1644.9732
a.i.
250
200
150
100
50
1000
1500
2000
2500
3000
m/z

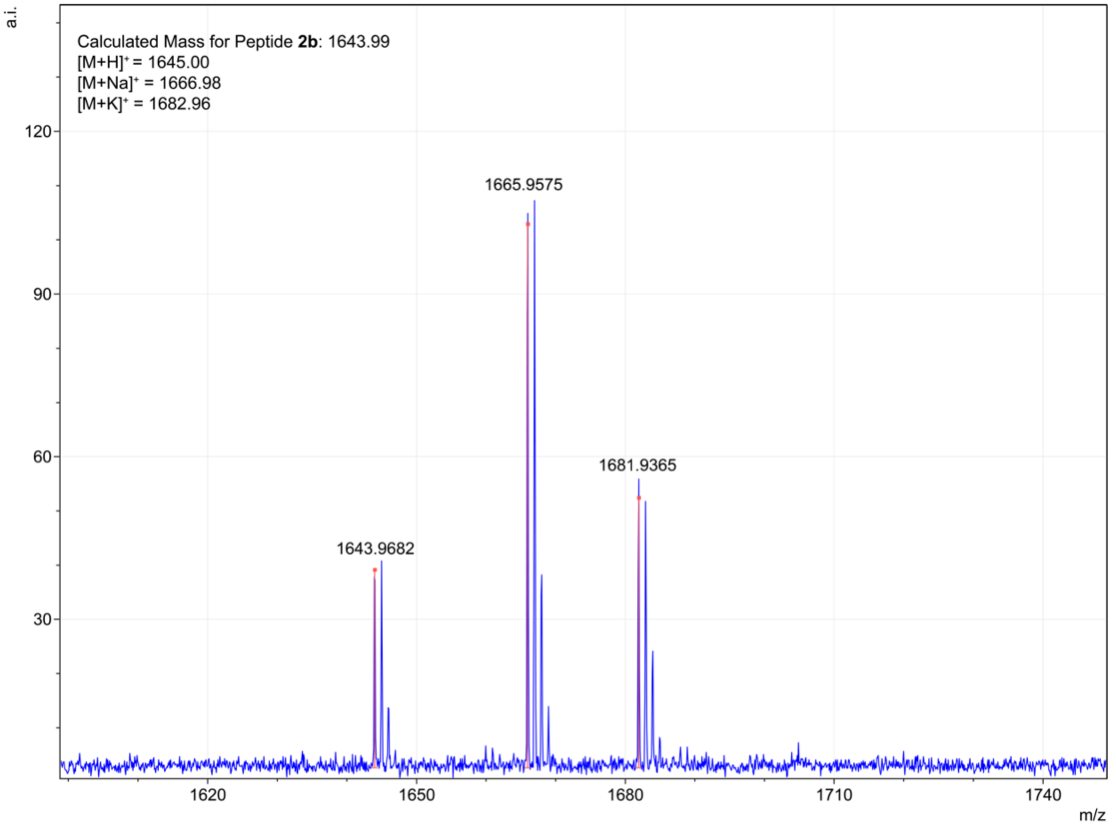

Calculated Mass for Peptide **2b**: 1643.99
[M+H]+ = 1645.00
[M+Na]+ = 1666.98
[M+K]+ = 1682.96
1665.9575
1681.9365
1643.9682
a.i.
120
90
60
30
1620
1650
1680
1710
1740
m/z
